# HNRNPA2/B1 is upregulated in endocrine-resistant LCC9 breast cancer cells and alters the miRNA transcriptome when overexpressed in MCF-7 cells

**DOI:** 10.1038/s41598-019-45636-8

**Published:** 2019-07-01

**Authors:** Carolyn M. Klinge, Kellianne M. Piell, Christine Schaner Tooley, Eric C. Rouchka

**Affiliations:** 1 Department of Biochemistry & Molecular Genetics, University of Louisville School of Medicine, Louisville, KY 40292 USA; 2 Department of Biochemistry, Jacobs School of Medicine and Biomedical Sciences, State University of New York at Buffalo, Buffalo, NY 14203 USA; 3 Bioinformatics and Biomedical Computing Laboratory, Department of Computer Engineering and Computer Science, University of Louisville, Louisville, KY 40292 USA

**Keywords:** miRNAs, Breast cancer

## Abstract

MicroRNAs are dysregulated in breast cancer. Heterogeneous Nuclear Ribonucleoprotein A2/B1 (HNRNPA2/B1) is a reader of the N(6)-methyladenosine (m6A) mark in primary-miRNAs (pri-miRNAs) and promotes DROSHA processing to precursor-miRNAs (pre-miRNAs). We examined the expression of writers, readers, and erasers of m6A and report that HNRNPA2/B1 expression is higher in tamoxifen-resistant LCC9 breast cancer cells as compared to parental, tamoxifen-sensitive MCF-7 cells. To examine how increased expression of HNRNPA2/B1 affects miRNA expression, HNRNPA2/B1 was transiently overexpressed (~5.4-fold) in MCF-7 cells for whole genome miRNA profiling (miRNA-seq). 148 and 88 miRNAs were up- and down-regulated, respectively, 48 h after transfection and 177 and 172 up- and down-regulated, respectively, 72 h after transfection. MetaCore Enrichment analysis identified progesterone receptor action and transforming growth factor β (TGFβ) signaling via miRNA in breast cancer as pathways downstream of the upregulated miRNAs and TGFβ signaling via SMADs and Notch signaling as pathways of the downregulated miRNAs. GO biological processes for mRNA targets of HNRNPA2/B1-regulated miRNAs included response to estradiol and cell-substrate adhesion. qPCR confirmed HNRNPA2B1 downregulation of miR-29a-3p, miR-29b-3p, and miR-222 and upregulation of miR-1266-5p, miR-1268a, miR-671-3p. Transient overexpression of HNRNPA2/B1 reduced MCF-7 sensitivity to 4-hydroxytamoxifen and fulvestrant, suggesting a role for HNRNPA2/B1 in endocrine-resistance.

## Introduction

The majority of breast tumors (70%) express estrogen receptor α (ERα) which is successfully targeted by adjuvant therapies that increase overall survival^1^. The current standard adjuvant treatments for patients with ERα+ breast cancer either inhibit ERα activity, *e.g*., tamoxifen (TAM) for premenopausal women, or block the conversion of androgens to estrogens by aromatase inhibitors (AIs), *e.g*., letrozole, in postmenopausal women^2^. Unfortunately, endocrine therapies are limited by the development of acquired endocrine resistance in ~30–40% of initially responsive patients that can occur up to 30 years after primary therapy^3,4^. A variety of mechanisms have been implicated in TAM-resistance (TAM-R)^5,6^, including altered microRNA (miRNA) and long noncoding RNA (lncRNA) expression^7–9^. Most miRNAs are transcribed, by RNA polymerase II, either as introns of host genes or as independent genes called primary (pri)-miRNAs^10^. Pri-miRNAs are processed by the DROSHA-DGCR8 microprocessor complex to precursor (pre)-miRNAs prior to nuclear export^11^. In the cytoplasm, the double stranded pre-miRNA is unwound by the DICER-TRBP complex to incorporate one strand of the miRNA (called the guide strand) into the RNA induced silencing (RISC) complex containing the catalytic Argonaut proteins, *e.g*., AGO2^12^. By basepairing with nucleotides in the 3′UTR of target genes within RISC, miRNAs can act as either oncomiRs by reducing protein levels of tumor suppressors or as tumor suppressors by decreasing oncogenic proteins in breast tumors^7^. The processing of pri-miRNA transcripts is regulated in part by post-transcriptional modifications (PTMs) of pri-miRNA^13^.

Next-generation sequencing (NGS) and mass spectrometry identified N(6)-methyladenosine (m6A) as the most common modification of mRNA and lncRNAs^14,15^. m6A plays a role in pre-mRNA processing, alternative splicing, nuclear export, stability, and translation^16,17^ by acting as a ‘conformational marker’ that induces sequence-dependent outcomes in RNA remodeling^18^. A recent report also identified higher m6A in selected pri-miRNA sequences that corresponded with increased levels of the corresponding mature miRNA in MDA-MB-231 triple negative breast cancer (TNBC) cells^13^.

m6A methylation is added by the RNA methyltransferase complex (WTAP, METTL3, METTL14, VIRMA, and RBM15), removed by the dioxygenases FTO and ALKBH5, and recognized by a variety of ‘readers’, including YTHDF1, YTHDF2, and HNRNPA2/B1^19–21^. METTL3 methylation of m6A on pri-miRNAs^13^ and RNA-dependent interaction of HNRNPA2/B1 with DGCR8, a component of the DROSHA complex, stimulate processing of selected pri-miRNA-m6A to precursor miRNA (pre-miRNA)^22^. HNRNPA2/B1 transcript expression is upregulated in breast tissue of postmenopausal parous women^23^, but its role in the protective effect of early pregnancy on postmenopausal ERα+ breast cancer is unknown^24^. HNRNPA2/B1 protein expression was higher in breast tumors compared to normal breast and knockdown of HNRNPA2/B1 inhibited the proliferation of MCF-7 and MDA-MB-231 breast cancer cells by causing S phase arrest and apoptosis^25^.

HNRNPA2 and HNRNPB1 are two splice isoforms transcribed from the same locus but are traditionally treated as a single protein^26^. HNRNPB1 is a lower abundance (~5%) N-terminal splice variant of the more highly expressed HNRNPA2 isoform and contains an additional 12 aa encoded by exon 2^27^. HNRNPA2/B1 share the remaining protein structure including an RNA-binding domain containing two RNA recognition motifs (RRMs) separated by a 15 aa linker and a C-terminal Gly-rich, low complexity region with a prion-like domain (PrLD), RGG box, and Py-motif including M9 nuclear localization signal^28^. In addition to its recognition of m6A in pri-miRNA and role in RNA splicing and processing^29^, HNRNPA2/B1 is involved in DNA repair^30^ and genome stability^31^.

In MCF-7 ERα+ breast cancer cells, enhanced cross-linking immunoprecipitation (eCLIP) using antibodies specific to HNRNPB1 alone or HNRNPA2/B1 in combination identified 1,472 transcripts bound by both HNRNPB1 and HNRNPA2/B1, 899 transcripts uniquely bound by HNRNPB1, and 479 transcripts uniquely bound by HNRNPA2/B1^32^. HNRNPB1 binding sites revealed a preference for 5′-AGGAAGG-3′ *versus* 5′-UGGGGA-3′ for HNRNPA2/B1^32^. HNRNPA2/B1 binding peaks were primarily in chromatin samples, consistent with HNRNPA2/B1 binding to nascent transcripts^32^.

Here we identified HNRNPA2/B1 expression to be higher in LCC9 and LY2 endocrine-resistant cells compared to parental MCF-7 luminal A breast cancer cells. We used miRNA-seq to identify differences in miRNA transcripts in MCF-7 cells when HNRNPA2/B1 is overexpressed and evaluated the pathways and mRNA targets associated with each misregulated miRNA for relevance to breast cancer and endocrine resistance. Progesterone receptor (PR) action in breast cancer and TGFβ signaling via miRNA in breast cancer were identified as pathways downstream of the upregulated miRNAs, and TGFβ signaling via SMADs and activation of Notch signaling were identified as pathways downstream of the downregulated miRNAs. TGFβ signaling, response to estradiol, and cell-substrate adhesion were pathways associated with mRNA targets of the identified miRNAs. Accordingly, overexpression of HNRNPA2/B1 in MCF-7 cells reduced their sensitivity to 4-hydroxytamoxifen and fulvestrant, indicating that increased HNRNPA2/B1 plays a role in tamoxifen and fulvestrant resistant cell proliferation.

## Results and Discussion

### Expression of RNA writers, readers, and erasers in breast cancer cells

**Figure 1:**
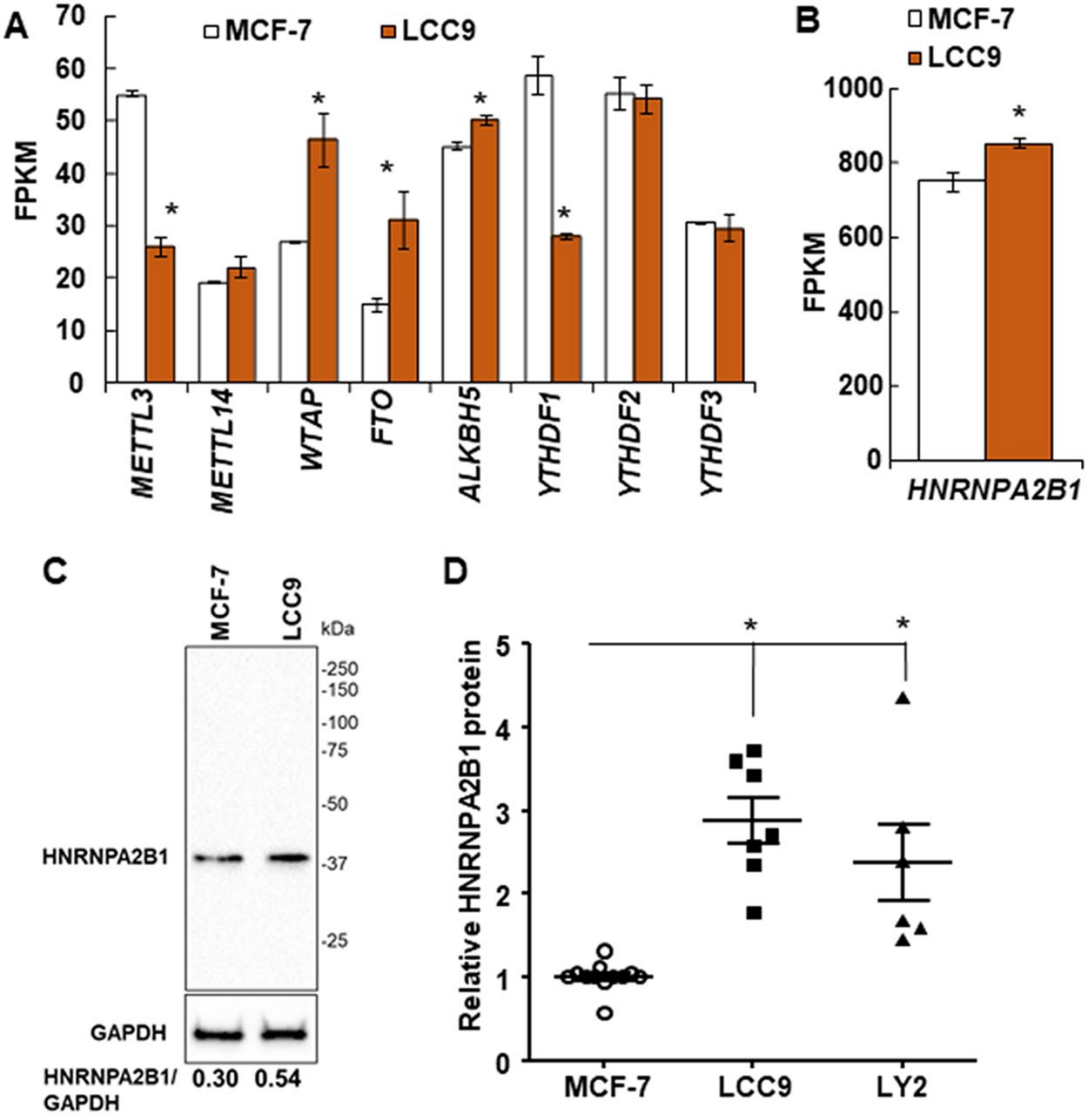
Expression of the genes encoding the readers, writers, and erasers of reversible m6A RNA modification. (**A,B**) Data are from a previous RNA-seq experiment in MCF-7 and LCC9 cells (GEO GSE81620). Data are the average of three replicate experiments +/− SEM. with FPKM = fragments Per Kilobase of transcript per Million mapped reads. *P < 0.05 in a two-tailed student’s t test. (**C**) Representative western blot of HNRNPA2B1 protein expression in WCE from MCF-7 and LCC9 cells. The blot was stripped and reprobed for GAPDH. The numerical values are HNRNPA2B1/GAPDH in these blots. The full-length blot of GAPDH is shown in Supplementary Fig. 1C. (**D**) Summary of relative HNRNPA2B1 protein expression in LCC9 and LY2 cells compared to MCF-7 parental cells. P < 0.05, One way ANOVA followed by Tukey’s test.

TAM/fulvestrant-resistant LCC9 breast cancer cells have higher levels of expression of diverse miRNAs compared with parental, TAM-sensitive MCF-7 cells^33^. To determine if there are differences in the expression of the genes encoding the readers, writers, and erasers of reversible m6A RNA modification^19^ between MCF-7 and LCC9 cells, we examined the steady state transcript levels of m6A writers (*WTAP, METTL3*, and *METTL14*), readers (*YTHDF1, YTHDF2, YTHDF3*, and *HNRNPA2/B1*) and erasers (*FTO* and *ALKBH5*) in RNA-seq data from our previous RNA-seq study, GEO accession number GSE81620^34^ (Fig. 1A). The expression of *METTL3* and *YTHDF1* transcripts was lower in LCC9 than MCF-7 cells whereas *WTAP, FTO*, *ALKBH5*, and *HNRNPA2/B1* were higher in LCC9 than MCF-7 cells. The possible role of the expression of *METTL3, YTHDF1, WTAP, FTO*, and *HNRNPA2/B1* transcripts in human breast tumors on overall survival was examined using the online tool Kaplan-Meier Plotter^35^. There was no association of overall survival (OS) for breast cancer patients based on primary tumor expression of *METTL3, YTHDF1*, or *WTAP* (Supplementary Fig. 1). Low expression of *FTO* was associated with lower OS (Supplementary Fig. 2A). However, higher FTO nuclear staining was reported in ER-/PR-/HER2+ breast tumors^36^. Patients with ER-/PR-/HER2+ breast tumors have ~40% lower disease-free survival compared to women with luminal A breast tumors^37^. *HNRNPA2/B1* transcript expression was higher than any of the other genes examined in the m6A pathway (Fig. 1B). HNRNPA2/B1 protein expression was also ~2.6-fold higher in LCC9 and LY2 cells than MCF-7 cells (Fig. 1C,D, Supplementary Fig. 3). Kaplan-Meier (K-M) survival analysis showed that higher expression of *HNRNPA2/B1* is associated with lower OS to ~150 months (Supplementary Fig. 2B). After ~220 months, the black line denoting high HNRNPA2B expression indicates reduced survival for those 3 patients in the K-M plot (Supplementary Fig. 2B). More data are needed to better understand whether low HNRNPA2B1 in the primary tumor predicts reduced OS after ~220 months. Thus, because of the high expression of *HNRNPA2B1* at the transcript and protein levels in LCC9 endocrine-resistant cells, its association with lower survival, and its role in increasing pri-miRNA processing^22^, we selected HNRNPA2B1 for further study.

### miRNA-seq analysis of HNRNPA2/B1-regulated miRNAs in MCF-7 cells

**Figure 2:**
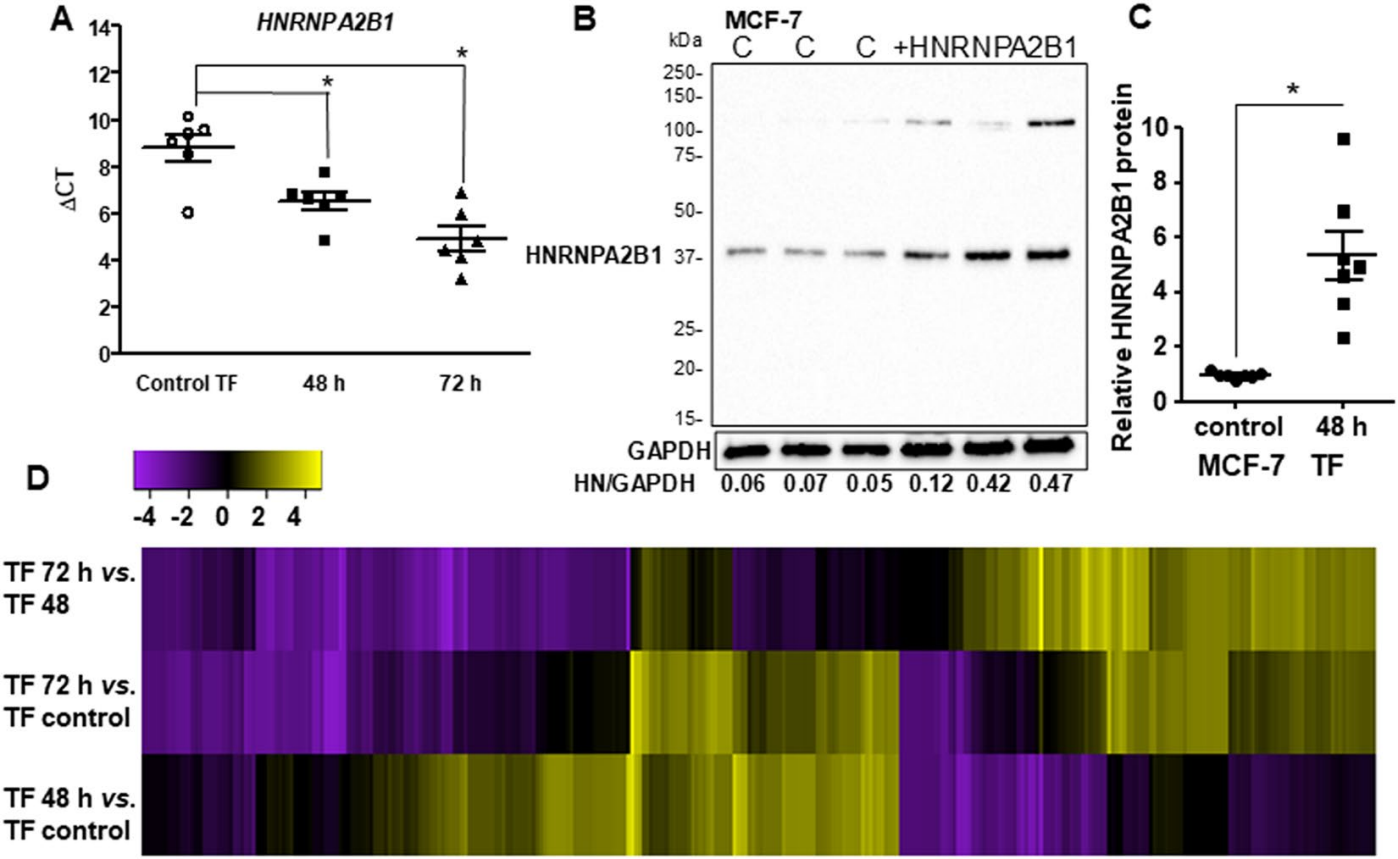
HNRNPA2B1 overexpression in MCF-7 cells. (**A**) The ΔCT values for HNRNPA2B1 normalized to 18 S of each of the six samples used for RNA se. MCF-7 cells were transfected with pCDNA3 control or pCDNA-3-HNRNPA2B1. Each point is the mean of triplicate determinations within one qPCR run of these samples. *p < 0.05, One way ANOVA followed by Tukey’s test. (**B**) Western blot for HNRNPA2B1 in MCF-7 cells control-transfected (**C**) and transfected with HNRNPA2B1 for 48 h. The blot was stripped and reprobed for GAPDH. Values are the HNRNPA2B1/GAPDH in this blot. The full-length blot of GAPDH is shown in Supplementary Fig. 1D. (**C**) Summary of relative HNRNPA2B1 protein expression in MCF-7 cells transfected for 48 h vs. control, n = mean ± std of 7 biological replicates. P < 0.0004, two-tailed student’s t-test. (**D**) The heat map represents the miRNAs having a fold-change of ±4. Yellow is upregulated and purple is downregulated (scale at top). Genes were clustered based on similar expression profiles.

Based on our observation of higher HNRNPA2/B1 in LCC9 compared to MCF-7 cells, we hypothesized that the overexpression of HNRNPA2/B1 in LCC9 cells promotes processing of pri-miRNAs resulting in increased pre- and mature miRNAs that act on targets and pathways to promote endocrine resistance. We note that HNRNPA2/B1 upregulated miR-99a, miR-125b, and miR-149 in MDA-MB-231 TNBC cells^22^, and we reported higher levels of miR-125b and miR-149, but not miR-99a, in LY2 endocrine resistant breast cancer cells as compared to MCF-7 cells in an earlier study^38^. To evaluate the effect of increased HNRNPA2/B1 on mature miRNA expression in breast cancer, MCF-7 cells were transiently transfected with a control vector for 48 h or an expression vector for HNRNPA2/B1 for 48 or 72 h (Fig. 2A). A limitation of this analysis was that a 72 h control-transfected group was not included. We did not detect differences in control gene (*GAPDH*) expression between 48 and 72 h control-transfected samples (Supplementary Fig. 3E). However, complete RNA transcriptome analysis of the 72 h control-transfected MCF-7 cells would have been a better control for the 72 h HNRNPA2/B1-transfected cells.

The transfection resulted in average ~5 fold increase in HNRNPA2/B1 protein expression (Fig. 2B,C). miRNA was isolated from six replicate experiments 48 or 72 h after HNRNPA2/B1 transfection for global changes in the miRNA transcriptome (miRome). Supplementary Table 1 shows a summary of the sequence analysis of the samples. A heatmap shows the relative consistency of miRNA expression changes in the replicate samples within each comparison and the changes between time after HNRNPA2/B1 transfection (Supplementary Fig. 4).

Three pairwise comparisons were evaluated: 48 h *versus* control, 72 h *versus* control, and 72 h *versus* 48 h. In total, 795 miRNAs were differentially expressed (p ≤ 0.05). 210 (110 up and 100 down) common to both time points, 236 (148 up and 88 down) uniquely at 48 h, and 349 (177 up and 172 down) uniquely at 72 h (Table 1). The identities and values of differentially expressed miRs are shown in Supplementary Tables 2–7 for all comparisons. Note that several miRs are listed twice, due to their coding from multiple gene locations. A heatmap for differentially expressed miRs passing a fold change (FC) threshold of ±4 (Log2FC ±2) in one or more of the comparisons is shown in Fig. 2D.

**Table 1:** Comparison of the number of differentially expressed miRNAs using a p-value cutoff of ≤0.05.

Comparison time transfected with HNRNPA2/B1	Total DE miRNAs	Upregulated miRNAs	Downregulated miRNA
48 h vs. control	236	148	88
72 h vs. control	349	177	172
72 h vs. 48 h	433	204	229

### miRNAs upregulated in HNRNPA2/B1-transfected MCF-7 cells

**Figure 3:**
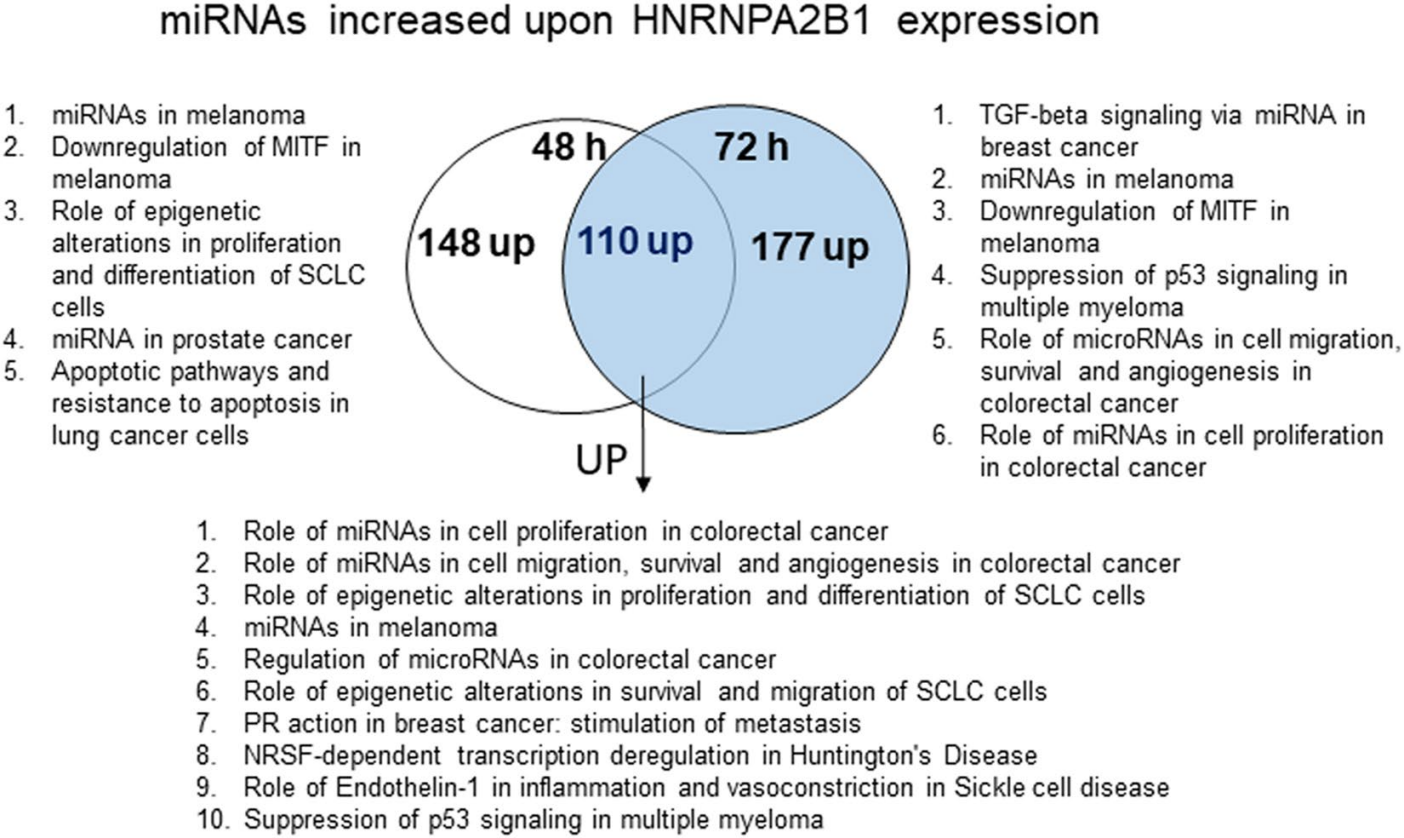
Venn diagram depicting the number of different and common miRNAs identified as upregulated after transient HNRNPA2B1 overexpression in MCF-7 cells after 48 or 72 h. MetaCore Enrichment by Pathway Maps analysis of DE miRNAs upregulated after 48 h (left, # 1–5) and 72 h (right, 1–6) (both versus control) and those identified in common at 48 and 72 h (below, #1–10).

Based on previous reports that HNRNPA2B1 increases processing of pri-miRNA to pre-miRNA and mature miRNAs^13,22^, we hypothesized that HNRNPA2/B1 overexpression would increase levels of miRNAs regulated by m6A in the respective pri-miRNA. We focus only on the miRNAs whose expression was significantly increased in response to HNRNPA2B1 transfection (Fig. 3, Tables 2, 3, Supplementary Table 8). Figure 3 shows that 148 and 177 miRNAs were uniquely increased at 48 and 72 h after HNRNPA2B1 transfection while 110 miRNAs were increased at both time points.

**Table 2:** Fourteen miRNAs were upregulated ≥2.0-fold by transient overexpression of HNRNPA2/B1 in MCF-7 cells at 48 and 72 h.

miRNA	logFC 48 h	logFC 72 h	Comments on role in breast or other cancers	Possible cellular role
miR-1266-3p	2.77	2.94	High breast tumor levels are a prognostic factor for recurrence-metastasis^61^	oncomiR
miR-2861	2.55	2.42	Higher expression in fulvestrant-resistant MCF-7 cells^43^. Increased in papillary thyroid carcinomas (PTC) with lymph node metastasis^74^.	Endocrine-resistance oncomiR
miR-4426	3.63	3.63	Downregulated in HER2-overexpressing MCF-7 cells^75^.	
miR-4667-5p	2.25	2.25	Upregulated in HER2-overexpressing MCF-7 cells^75^	
miR-4739	3.03	3.03	Increased by si-CTNNB1 (β-catenin) in gastric cancer (GC) cells, implying a tumor suppressive function in GC^76^.	Tumor suppressor
miR-6087	2.15	2.03	Downregulated in Adriamycin-resistant MCF-7 cells^77^.	
miR-6088	2.63	2.42	Increased by the natural sweetener steviol in HCT-116 cells^78^.	
miR-6762-5p	2.95	2.41	No references found	
miR-6771-5p	2.49	2.26	No references found	
miR-6801-3p	3.19	3.03	No references found	
miR-6803-5p	2.45	2.00	No references found	
miR-6805-3p	2.79	2.40	Increased by 1 nM progestin R5020 in T47D, BT474, and ZR-75-1 BCa cells, but its role was not examined^79^	
miR-7107-5p	2.13	3.03	Higher breast tumor expression levels associated with acquired resistance to chemotherapy^63^.	oncomiR chemo-resistance
miR-762	3.23	2.41	promotes BrCa cell proliferation & invasion by targeting IRF7^80^. Directly targets tumor suppressor MEN1 in ovarian cancer and promotes metastasis^81^.	oncomiR

miRNAs are sorted by name. The log fold change (logFC) is the average of 6 biological replicate samples and all are statistically significant as indicated by the p ≤ 0.05. The apparent cellular role is based on publications cited related to breast or other cancers as indicated as found in PubMed and Google Scholar.

Fourteen miRNAs were increased by ≥2.0-log fold at both 48 and 72 h (Table 2). Of the six miRNAs on which publications were found, four (miR-1266, miR-2861, miR-7107-5p, and miR-762) have oncogenic, endocrine- or chemo-resistance activities in breast cancer (Table 2). Sixty miRNAs were increased at 48 h, but not 72 h (Table 3, Supplementary Table 8). Of the twenty miRNAs with publications, seven had reported oncomiR functions and six had tumor suppressor functions. HNRNPA2/B1 transfection increased miR-222-5p, which is increased in TAM-R MCF-7 cells, and its role in TAM-R and targets, including *ESR1* (ERα) and cell cycle genes, *e.g*., *CDKN1B* (P27/KIP1) have been reviewed^7,39^. While miR-595 has no established role in breast cancer, it has tumor promoter roles in papillary thyroid carcinoma (PTC)^40^ and human glioblastoma (GBM) cells^41^. However, miR-595 acts as a tumor suppressor in ovarian cancer^42^; thus, its role in breast cancer remains to be determined.

**Table 3:** Sixty miRNAs were upregulated ≥2.0-fold by transient overexpression of HNRNPA2/B1 in MCF-7 cells at 48 h, but not at 72 h.

miRNA	logFC	Comments on role in breast or other cancers	Possible cellular role
miR-1233-3p	2.21	Increased in serum of renal cell carcinoma (RCC) patients^82^; represses *GDF15*^83^	oncomiR
miR-1279	2.55	No references found	
miR-1538	2.13	Identified in serum of neuroblastoma patients^84^.	
miR-212-5p	2.15	overexpressed in drug-resistant breast tumors and DOX-resistant MCF-7 cells, targets *PTEN*^85^	oncomiR
miR-217	1.39	Expression correlated with ER+ in breast tumors^86^; higher in TNBC tumors than ER+ tumors, correlated with tumor grade and cancer stage and targeted *DACH1*^87^; overexpression in MCF-7 cells reduced TAM-sensitivity, reduced E-cadherin, increased invasion and *SNAI1* (Snail), and downregulated PTEN^88^; targets *PPARGC1A* (PGC-1α) in breast cancer cells^89^; miR-217 inhibitor blocks docetaxel- or cisplatin-induced apoptosis in MCF-7 cells^90^; acts as a tumor suppressor by targeting *KLF5* in TNBC cells^91^. Directly targets *SIRT1*^92^.	oncomiR TAM-R Tumor suppressor
miR-222-5p	2.59	Increased in TAM-R cells^54^; Roles in BCa and TAM-R reviewed^7,34,39^; high levels are associated with breast tumor stage^93^. Directly targets *SSSCA1* (P27)^94^.	oncomiR TAM-R
miR-302c-3p	2.60	Expression correlated with HER2+ in breast tumors^86^ and development of breast cancer^95^; Directly targets *ESR1* (ERα)^96^, *CCND1* (Cyclin D1)^97^; *BCRP*^98^; *MEKK1*^99^	oncomiR
miR-3129-3p	2.57	Downregulated in epithelial ovarian cancer (EOC) cell lines and overexpression by lentiviral transduction inhibited EOC cell proliferation *in vitro* and tumor xenograft growth *in vivo*^100^.	Tumor suppressor in EOC
miR-3132	2.13	Upregulated in A549, HUVEC and THP-1 cells infected with a hantavirus (Prospect Hill virus)^101^	
miR-3135a	2.60	No references relevant to human cancer were found	
miR-3168	2.60	Upregulated by 2 nM paclitaxel treatment in HepG2 cells and thought to play a role in paclitaxel resistance^102^.	
miR-3195	2.42	Related to metastases^103^	
miR-3610	2.58	Upregulated in BCa tissues^104^.	
miR-3619-3p	2.20	High expression in MCF-7 cells and acts as a tumor suppressor by directly targeting *PLD* (phospholipase D)^105^; higher expression correlated with tumor relapse in small cell carcinoma of the esophagus^106^	tumor suppressor
miR-3655	3.26	Upregulated in metastatic melanoma^107^.	
miR-3674	2.40	No references relevant to cancer were found	
miR-3919	2.60	No references relevant to cancer were found	
miR-3923	2.13	Downregulated in primary breast tumors with lymph node metastasis^108^.	tumor suppressor
miR-3938	2.57	No references relevant to cancer were found	
miR-3944-5p	2.00	Upregulated by hypoxia in AC16 cardiomyocytes^109^.	
miR-3960	2.24	No references found re. experimental evidence for miR-3960 in cancer.	
miR-410-5p	2.00	Located in the DLK1-DIO3 genomic region 14q32 that has 2 maternally and 3 paternally expressed genes, 2 lncRNAs, and 53 miRNAs^110^; high expression favorable in gastric, ovarian, and lung cancer^110^; miR-410-3p is downregulated in breast tumors and acts as a tumor suppressor by targeting *SNAI1*^111^; Suppresses cell growth, migration, and invasion and enhances apoptosis in MCF-7 and T47D cells; directly targets *ERLIN2*^112^; Directly targets *STAT3*^113^.	tumor suppressor
miR-4459	2.38	Upregulated in exosomes derived from chemoresistant ovarian cancer cells^114^. Identified as specific for ERBB2 breast tumors^115^.	
miR-4463	2.60	Increased in serum of PCOS patients^116^	
miR-4524b-5p	3.29	Increased in salivary glands from Sjögren syndrome patients^117^.	
miR-4532	2.07	Increased expression in MCF-7 CSC-mammospheres- spheroid culture^118^; Higher in fulvestrant-resistant MCF-7 cells^43^	oncomiR TAM-R
miR-4634	3.86	Expression similar in serum from BC patients vs controls^119^.	
miR-4653-5p	3.86	None found, but miR-4653-3p was decreased in recurrent/metastatic lesions compared to the matched ER+/PR+ primary breast tumors^120^	
miR-4657	2.13	Downregulated in metformin-treated cholangiocarcinoma tumor cell lines^121^.	
miR-4665-5p	2.60	Upregulated by mechanical compression of cancer-associated fibroblasts (CAFs) from invasive ductal carcinomas^122^.	
miR-4679	2.13	Upregulated in VEGF-overexpressing K562 leukemia cells^123^.	
miR-4701-3p	2.21	Downregulated in fulvestrant-resistant MCF-7 cells^43^. Upregulated in plasma of PTC patients^124^	
miR-4717-5p	2.60	miR-4717-3p was downregulated in the blood of 6 breast cancer patients^125^.	
miR-4723-3p	2.60	Downregulated in prostate tumors and acts as a tumor suppressor by targeting *ABL1*^126^.	tumor suppressor in PCA
miR-4739	2.60	Increased by si-CTNNB1 (β-catenin) in gastric cancer (GC) cells, implying a tumor suppressive function in GC^76^.	
miR-4750-5p	3.45	Computational studies identified a binding site for miR-4750-5p in *TBC1D17* that has a role in breast cancer, but direct interaction was not verified^127^.	
miR-4752	2.60	No references found	
miR-4755-3p	3.24	No references relevant to breast or other cancers was found	
miR-4763-5p	2.60	Increased in blood from multiple myeloma patients^128^.	
miR-4787-5p	2.98	Downregulated in human pancreatic ductal adenocarcinomas^129^. Upregulated in plasma as a specific biomarker of lung squamous cell carcinoma^130^.	
miR-4800-3p	4.20	Upregulated in MDA-MB-231 and Hs578T TNBC cells compared to MCF-7 and SK-BR-3 cells^131^.	
miR-500b-3p	2.45	Higher in blood from patients with synovial sarcoma^132^ and prostate cancer (PCA)^133^ than controls.	
miR-507	2.57	Apparent tumor suppressor: lower in breast tumors and cell lines than non-neoplastic tissue and cells and directly targets *FLT1*^134^.	tumor suppressor in BC
miR-5188	2.13	Downregulated in siHER2-transfected BT474 cells^75^	
miR-548f-3p	2.35	Upregulated in metformin-treated cholangiocarcinoma tumor cell lines^121^.	
miR-548g-3p	2.39	Directly targets the stem loop A promoter element within the 5′UTR of dengue virus and represses replication^135^.	
miR-5572	2.13	Identified in minor salivary glands of Sjögren’s syndrome patients, in Jurkat cells, and in immortalized human salivary gland cell line pHSG^136^	
miR-5581-5p	2.21	Upregulated in vulvar squamous cell carcinomas^137^	
miR-587	2.60	Higher expression in chemoresistant colorectal tumors and modulates drug resistance by directly targeting PPP2R1B in colorectal cancer cells^138^.	oncomiR in colo-rectal cancer
miR-595	2.66	Commonly overexpressed in endocrine cancers, including PTC^40^; tumor promoter in human glioblastoma (GBM) cells by directly targeting *SOX7*^41^. Downregulated in ovarian cancer tissues and directly targets *ABCB1*^42^.	oncomiR and tumor suppressor
miR-6075	2.75	Increased expression in pancreato-biliary cancer^139^	
miR-6501-5p	2.41	No references found	
miR-6515-3p	2.18	Increased in blood from vitiligo patients^140^	
miR-6723-5p	2.26	Increased by xanthohumol (a hop plant extract that reduces cell viability) treatment of U87 MG glioma cells^141^.	
miR-6741-3p	2.60	Upregulated in the blood of Systemic Lupus Erythematous patients with class IV lupus nephritis^142^.	
miR-6762-5p	2.95	Identified as a potential target of hsa-circ-0036722, but not experimentally validated^143^	
miR-6773-5p	2.53	Downregulated by cyclosporine A treatment of HK‐2 immortalized proximal tubule epithelial cells^144^.	
miR-6836-3p	2.21	Upregulated in MDA-MB-231 and Hs578T TNBC cells compared to MCF-7 and SK-BR-3 cells^131^.	
miR-6882-5p	2.13	Identified as a biomarker for pancreatic ductal adenocarcinoma^145^	
miR-6886-3p	2.58	Downregulated by the lncRNA HULC in hepatocellular carcinoma (HCC) and directly targets *USP22*^146^.	
miR-7109-5p	2.31	No references found	
miR-8079	2.13	No references found	

The miRNAs are sorted by name. The logFC is the average of 6 biological replicate samples and all are statistically significant as indicated. The apparent cellular role is based on publications cited related to breast or other cancers as found in PubMed and Google Scholar. We found published information on 14 of these 60 miRNAs with 8 having oncomiR and 7 had tumor suppressor roles in breast or other cancer.

Fifty-one miRNAs were increased at 72 h, but were not increased at 48 h (Supplementary Table 8). Of the 17 miRNAs with published information relevant to cancer, 3 miRNAs (miR-4763-3p, miR-4787-5p, and miR-4800-3p) were reported to be higher in fulvestrant-resistant MCF-7 *versus* TAM-resistant MCF-7 cells^43^.

**Figure 4:**
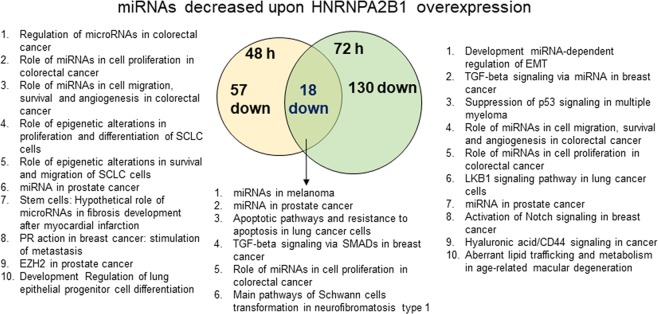
Venn diagram depicting the number of different and common miRNAs identified as downregulated after transient HNRNPA2B1 overexpression in MCF-7 cells after 48 or 72 h. MetaCore Enrichment by Pathway Maps analysis of DE downregulated miRNAs after 48 h and 72 h (both versus control) identified common and unique pathways putatively regulated by the DE miRNAs.

MetaCore analysis was performed on all three groups of upregulated miRNAs (common to 48 and 72 h, unique to 48 h, and unique to 72 h (Fig. 3). Pathways identified included “PR action in breast cancer: stimulation of metastasis” (involving downregulation of miR-29a-3p) and “TGFβ signaling via miRNA in breast cancer” (involving downregulation of miR-21-5p, miR-200a-3p, miR-200a-5p, miR-200c-3p, miR-200c-5p, miR-200b-3p, upregulation of miR-181a-5p) (Fig. 4). miR-200 family members are downregulated in breast cancer and in TAM-R breast cancer cell lines and tumors (reviewed in^7,44^). The decrease in miR-200 family members would be expected to relieve repression of ZEB1/2 leading to repression of E-cadherin and EMT, an indicator of breast cancer progression and metastasis^45^. The GO processes identified included “Cellular response to estrogen stimulus” (upregulation of miR-574-5p and miR-466) (Supplementary Fig. 6, Supplementary Table 2). Increased serum of miR-574-5p is a marker of breast cancer^46^.

### miRNAs downregulated in HNRNPA2/B1-transfected MCF-7 cells

Unexpectedly, we identified 88, 172, and 100 miRNAs downregulated at 48 h, 72 h, and both time points, respectively (Supplementary Fig. 5). This is the first identification of miRNAs downregulated in response to HNRNPA2/B1 overexpression. Of course, this could be a direct or indirect effect. Another HNRNP family member, HNRNPA1, can either promote or inhibit pri-miRNA processing, resulting in increased mature miR-18a^47^ and reduced let-7a-1 in HeLa cells^48^. We did not detect any significant increase in miR-18a nor a decrease in let-7a-1 in HNRNPA2/B1-transfected MCF-7 cells, implying that these two HNRNPs have different targets in different cells.

We focused on those downregulated ≥2.0-log fold: 57 at 48 h, 130 at 72 h, and 18 in common (Fig. 4, Supplementary Table 7). Nine of the 18 common downregulated miRNAs had roles in breast cancer, including miR-221-3p and miR-222-3p (both target *ESR1*) and miR-515-5p and miR-516-5p, which are increased in TAM-R MCF-7 cells (Table 4). Of the 57 miRNAs downregulated at 48 h (Table 5), 26 have reported roles in breast cancer. Some have roles in TAM-R. let-7i and miR-489 are downregulated in TAM-resistant breast cancer cells and miR-101, miR-221, miR-222, and miR-515 are upregulated in TAM-resistant MCF-7 and other breast cancer cells^7,39,49^. Both miR-29a-3p and miR-29b-3p which reduce TAM-resistant MCF-7 cell (LCC9 and LY2 cell lines) proliferation, migration, and colony formation^33^ were downregulated by HNRNPA2/B1 overexpression.

**Table 4:** Eighteen miRNAs were downregulated by transient overexpression of HNRNPA2/B1 in MCF-7 cells at 48 and 72 h.

miRNA	logFC	Comments on role in breast or other cancers	Possible Cellular role
miR-1283	−2.27	*ATF1* rs11169571 variant C, associated with increased colorectal cancer risk, inhibits binding of miR-1283^147^. Downregulated in plasma from stage IV and stage III melanoma patients^148^. Overexpressed in malignant Spitz lesions from melanoma tumors^149^	
miR-221-3p	−1.16	High expression in breast tumors vs. normal breast^150^. Higher expression in TAM-resistant MCF-7 cells^54^ and higher in MDA-MB-231 cells than MCF-7 cells^151^. Targets *ESR1*^152^, *BBC3* (PUMA)^153^, TRPS1^154^, *BIM*^155^, *NOTCH3*^156^, *TNFAIP3* (A20)^157^, PARP1^158^, and is involved in EMT^159^	oncomiR
miR-222-3p	−0.96	High expression associates with high tumor stage, Ki-67 staining, luminal B, and HER2 amplification in breast tumors^93^. Increased in TAM-R BCa cells and targets *ESR1*, *ERBB3* (reviewed in^7^).	oncomiR
miR-224-5p	−2.86	Higher expression in TNBC than in ER+ /PR+/HER2 breast tumors^160^. High expression in TNBC is associated with lower OS^161^. Downregulated in aromatase-resistant BCa cells^162^. *SMAD4* is a direct target^163^.	
miR-4458	−2.34	Deregulated in fulvestrant-resistant MCF-7 cells^43^	
miR-4724-3p	−2.63	No reports for breast or other cancers.	
miR-4738-5p	−2.37	No reports for breast or other cancers.	
miR-486-5p	−1.24	Downregulated in exosomes in serum from BCa patients with recurrence^59^.	Tumor suppressor
miR-489-5p	−1.67	Reduced in BCa tumors^164^. Metastasis suppressor^165^. reduced in TAM-resistant MCF-7 cells^54^.	Tumor suppressor
miR-5008-3p	−2.63	No reports for breast or other cancers.	
miR-511-5p	−2.37	No reports for breast or other solid cancers.	
miR-515-5p	−3.57	Suppressed by E_2_ in MCF-7 cells^166^, E_2_-ERα-downregulated and TAM- ERα-upregulated; lower in ER- breast tumors and downregulates oncogenic genes in the WNT-signaling pathway^167^.	Tumor suppressor
miR-516a-5p	−2.31	Increased expression in TAM-R MCF-7 cells^168^; miR-516a-5p targets *MARK4*, a regulator of the cytoskeleton and cell motility, in BCa^169^.	Tumor suppressor
miR-518c-3p	−3.39	No reports for breast or other cancers, but upregulated by E_2_ in MCF-7 cells^170^.	
miR-518d-5p	−2.37	No reports for BCa. Downregulated by CircRNA8924 that acts as a competitive endogenous RNA of miR-51d-5p and miR-519-5p in cervical cancer^171^.	
miR-520c-5p	−2.37	miR-520c is oncogenic in TNBC^172^	Oncogenic in TNBC
miR-526a	−2.37	Transient overexpression of miR-526a mimics stimulated MCF-7 cell proliferation^173^.	
miR-6850-3p	−2.05	No reports for breast or other cancers.	

miRNAs are sorted by name. LogFC is the average of 6 biological replicate samples and all are statistically significant as indicated by the p ≤ 0.05. The apparent cellular role is based on publications cited related to breast or other cancers as indicated as found in PubMed and Google Scholar.

**Table 5:** Fifty-seven miRNAs were downregulated by transient overexpression of HNRNPA2/B1 in MCF-7 cells at 48 h.

miRNA	logFC	Comments on role in breast or other cancers is information on breast cancer not identified in PubMed	Apparent Cellular role
let-7f-2-3p	−0.75	Low let-7f-2 predicts colon cancer progression^174^; upregulated in renal cancer^175^.	
let-7i-3p	−0.76	TAM-sensitivity of ZR-75-1 BCa cells was increased by let-7i transfection^176^.	
miR-100-5p	−2.90	Down-regulated in CSC generated from MDA-MB-231 TNBC cells^177^; miR-100 inhibits CSC in basal-like breast cancer and low miR-100 is a negative prognostic factor^178^	
miR-101-3p	−0.61	miR-101 transcripts on different chromosomes play diverse roles in the diagnosis, prognosis, and clinical outcome of BC^179^. miR-101-1, closely linked to ER, PR, and HER2, is processed into miR-101-3p and miR-101-5p, while miR-101-2 lower in expression in BC tumors than normal breast tissue, only produces mature miR-101-3p^179^. *AMPK* is a verified direct target of miR-101-3p^180^. Overexpression of miR-101 promotes E_2_-independent growth and TAM-R of MCF-7 cells^181^.	Putative oncomiR in BCa (reviewed in^7^).Tumor suppressor in breast cancer^180^
miR-101-5p	−1.09	Downregulated in HCC tumors and is a “potential diagnostic marker” for HCC^182^	Putative tumor suppressor
miR-1251-5p	−1.36	No reports for breast or other cancers.	
miR-1323	−3.60	Higher expression in locally advanced esophageal squamous cell carcinoma tumors that are resistant to radiotherapy^183^. Upregulated in radiation-resistant A549 NSCLC cells^184^ and in radiation-resistant nasopharyngeal carcinoma cells^185^; High expression in cirrhosis-associated HCC correlated with “dismal survival and advanced staging”^186^	oncomiR
miR-134-3p	−2.37	Suppresses ovarian cancer stem cell biogenesis by directly targeting *RAB27A*^187^	Tumor suppressor
miR-135a-5p	−2.30	Reported to be highly expressed in breast tumors (n = 20)^188^; Upregulated by E_2_ in MCF-7 cells^189^; Directly targets *ESRRA* (ERRα)^190^ and *ELK1* and *ELK3* oncogenes^191^.	Tumor suppressor
miR-138-5p	−1.83	Upregulated in the circulation of patients with breast cancer, but there was no change observed in the tumor tissue^192^; Downregulated in breast tumors and lower expression was associated with advanced clinical tumor stage and metastatic disease^193^; directly targets VIM (vimentin) and inhibits cell invasion, migration, and proliferation in BCa cell lines^193^;	Tumor suppressor^193^
miR-145-5p	−1.85	Induced by p53 and directly targets *MYC*^194^ and *RPS6KB1* (P70S6K1)^195^. Overexpression abrogates the oncogenic activity of circZNF609 in BCa cells; further, circZNF609 and miR-145-5p are strongly negatively correlated in breast tumors^196^. linc01561 was a ceRNA of miR-145-5p in BCa cells^197^.	
miR-17-5p	−0.77	Higher expression in TNBC *versus* luminal A invasive breast ductal carcinoma^198^. Downregulates E_2_-ERα-regulated gene expression by downregulating coactivator *NCOA3* (AIB1, SRC-3) in MCF-7 cells^199^. Directly targets *HBP1* to promote invasion and migration of BCa cells^200^. Directly targets DR4 and DR5 to inhibit TRAIL-induced apoptosis in BCa cells^201^. Suppresses TNBC cell proliferation and invasion by targeting *ETV1*^202^. Downregulated in exosomes from BCa patients with recurrence^59^. Directly inhibits translation of *NCOA3* (AIB1) in BCa cells^199^	Tumor suppressor^203^; anti-metastatic function in basal breast tumors^204^; oncomiR in other cancers^205^
miR-187-3p	−0.91	Higher expression in sporadic BCa than in tumors from women with BRCA1 or BRCA2 mutations^206^. Down-regulated in colorectal, prostate, lung, RCC, and HCC^207^.	
miR-193a-3p	−0.82	Acts as a tumor suppressor by targeting *HIC2, HOXC9, PSEN1, LOXL4, ING5, c-KIT, PLAU, MCL, SRSF2*, and *WT1*^208^. Upregulated in fulvestrant-resistant MCF-7 cells^43^. miR-193a gene is silenced by methylation and directly targets GRB7 in ovarian cancer^209^. Only miR-193a-5p was downregulated in breast tumors whereas no difference was observed in the expression levels of miR-193-3p in BCa *versus* normal tissues^210^. Both miR193a-5p and miR-193a-3p repressed MCF-7 and MDA-MB-231 cell proliferation by different targets. miR-193a-3p suppressed cell growth by inhibiting *CCND1, PLAU*, and *SEPN1* and inhibited cell motility by suppressing *PLAU* expression	Tumor suppressor in many cancers.
miR-19a-3p	−0.70	Lower in MCF-7 than MDA-MB-231 cells^211^.	oncomiR in breast cancer cells^212^
miR-19b-3p	−0.82	Expression level was significantly down‐regulated in BCa vs normal breast^213^. Downregulated in SK-BR-3 cells resistant to the PI3K inhibitor saracatinib and miR-19b-3p directly targets *PIK3CA*^214^	Tumor suppressor
miR-20a-5p	−0.64	Higher in TNBC than luminal A invasive breast ductal cancer^198^. LncRNA *HOTAIR* is overexpressed in breast tumors and directly binds downregulates miR-20a-5p^215^. Directly targets *HMGA2*^215^ and *RUNX3*^216^.	Tumor suppressor
miR-26a-1-3p	−1.06	Expression correlates with ER+/PR+ in breast tumors^217^. Direct targets include *CCNE1, CDC2*, and *EZH2*^217^; *CHD1, GREB1* and *KPNA2*^218^, and *MCL1*^219^. Downregulated by E_2_ in MCF-7 cells^220^. Over-expression inhibited the growth of SKBR3 and BT474 cells^221^ and of MDA-MB-231, MDA-MB-468, and MCF-7 cells^222^. However, overexpression for ≥ 3 d results in aneuploidy in human BCa cells^223^.	
miR-29a-3p	−0.97	stimulates migration and invasion; Repressed by MYC, YYI, NFκB, CEBPA and stimulated by p53^224^.	OncomiR and tumor suppressor
miR-29b-3p	−0.71	Low expression in breast tumors correlates with reduced OS and DFS^225^. Lower expression in invasive ductal adenocarcinoma *versu*s lobular carcinomas and elevated in ER+ *versus* ER- breast tumors^58^.. Expression increased by GATA2 which suppresses *MMP9, ANGPTL4, VEGF*, and *LOX* promoting differentiation, blocking EMT to suppress metastasis^226^. Regulated by S100A7 acting as an oncogene in ER-negative and as a cancer-suppressor in ER-positive BCa cells, with miR-29b being the determining regulatory factor^227^.	Tumor suppressor
miR-3125	−3.38	No reports for BCa. Lower in glioblastoma tissues as a poor prognostic indicator^228^.	
miR-320e	−0.88	No reports for breast cancer. Significantly higher expression in stage III colon cancers from patients with recurrence and associated with poorer DFS^229^.	
miR-34b-5p	−2.20	Downregulated in breast tumors^166,230^.	Tumor suppressor
miR-34c-5p	−2.36	Downregulated in breast tumors^231^	Tumor suppressor
miR-3591-5p	−2.88	No reports for BCa. Expression increased after radiation of A549 NSCLC cells and Ubiquitin Specific Peptidase 33 (USP33) was a downstream target of miR-3591-5p^232^.	
miR-3663-5p	−2.96	No reports for BCa. Expression increased in human nonalcoholic fatty liver disease (NAFLD)^233^.	
miR-3912-3p	−0.72	No reports for breast or other cancers.	
miR-424-5p	−1.18	Increased in serum of BCa patients with resistance to dovitinib^234^. Low in basal-like breast tumors^235^.	Tumor suppressor
miR-4419a	−2.37	No reports for breast or other cancers.	
miR-4500	−2.08	No reports for BCa, but down regulated in colorectal cancer and downregulates HMGA2^236^.	
miR-4764-3p	−2.37	Downregulated in HER2-overexpressing MCF-7 cells^75^.	
miR-4767	−1.63	No reports for breast or other cancers.	
miR-4789-3p	−2.16	Identified as specific for basal breast tumors^115^.	
miR-4790-3p	−1.66	No reports for breast or other cancers.	
miR-4793-3p	−2.37	No reports for breast or other cancers.	
miR-488-5p	−2.83	Down regulated in breast tumors, but upregulated in plasma of patients with recurrent BCa^237^.	Tumor suppressor
miR-497-3p	−0.66	Higher in ER+ Breast tumors and directly targets *ERRA*^238^.	Tumor suppressor
miR-520g-3p	−2.63	miR-520g is higher in ER-/PR- breast tumors^86^. Plasma miR-520g expression levels were significantly higher in BC patients with lymph node metastatic disease^239^.	Oncogenic
miR-548ao-3p	−3.79	Specifically upregulated in TNBC tumors^240^	
miR-551b-3p	−1.99	Lower in breast tumors and characterized as a tumor suppressor^241^.	Tumor suppressor
miR-5584-5p	−2.63	No reports for breast or other cancers.	
miR-5681a	−2.07	No reports for breast or other cancers.	
miR-5682	−2.37	No reports for breast or other cancers.	
miR-5692a	−3.51	Overexpressed in HCC tumors and knockdown inhibited HCC cell growth and invasion^242^.	
miR-652-5p	−0.60	Lower in breast tumors versus adjacent normal tissue^243^. miR-652-3p levels were significantly lower in the serum of BCa patients than that in controls^244^.	Tumor suppressor
miR-659-5p	−1.39	Upregulated in rectal tumors from smokers^245^.	
miR-6716-3p	−1.61	No reports for breast or other cancers.	
miR-6733-3p	−1.74	No reports for breast or other cancers.	
miR-6794-3p	−2.57	No reports for breast or other cancers.	
miR-6795-3p	−2.90	No reports for breast or other cancers.	
miR-6834-5p	−2.37	No reports for breast or other cancers.	
miR-6878-5p	−2.37	No reports for breast or other cancers.	
miR-7975	−2.00	No reports for breast or other cancers.	
miR-934	−1.46	Upregulated in breast tumors diagnosed ≤ 5.2 years postpartum in Hispanic women^246^.	
miR-937-3p	−2.17	No reports for breast or other solid cancers.	
miR-944	−1.57	Increased by E_2_ in MCF-7 cells^189^. Higher in breast tumors and serum *versus* controls and targets *BNIP3*^247^. However, another study reported that miR-944 expression was repressed in breast tumors and cell lines^248^. miR-944 overexpression inhibited cell migration/invasion and directly targeted *SIAH1* and *PTP4A1*^248^. miR-944 also inhibits metastasis of gastric cancer cells by inhibiting EMT by targeting *MACC1*^249^.	Cisplatin-resistance Tumor suppressors
miR-98-3p	−0.78	Increased by E_2_ in MCF-7 cells^250^. Downregulated in fulvestrant-resistant MCF-7 cells^43^. Inhibition of endogenous miR-98 in 4T1 mouse BCa cells promoted cell proliferation, survival, tumor growth, invasion, and angiogenesis^251^. miR-98 directly targets ALK4, MMP11, and HMGA2^252^.	Tumor suppressor

miRNAs are sorted by name. LogFC is the average of 6 biological replicate samples and all are statistically significant as indicated by the p ≤ 0.05. The apparent cellular role is based on publications cited related to breast or other cancers as indicated as found in PubMed.

MetaCore pathway analysis identified “TGFβ signaling via SMADs in breast cancer” for the common 18 downregulated miRNAs, as well as “PR action in breast cancer: stimulation of metastasis” in the 48 h and “Activation of Notch signaling in breast cancer” in the 72 h downregulated miRNAs (Fig. 4). MetaCore enrichment analysis by GO processes identified “cellular response to estrogen stimulus (miR-206)” and “response to estrogen” (also miR-206) (Supplementary Fig. 7). E_2_, and the ER-selective agonist PPT suppressed miR-206 expression in MCF-7 cells^50^.

### Identification of experimentally validated gene targets of the miRNAs differentially expressed in MCF-7 cells transfected with HNRNPA2/B1

The differentially expressed miRNAs were searched against miRTarBase^51^ for experimentally validated gene targets. Table 6 shows the number of differentially expressed miRNAs and the number of validated gene targets for these miRNAs. Genes identified as targets of the DE miRNAs were used as an input into categoryCompare^52^ to identify enriched Gene Ontology Biological Processes (GO:BP)^52^.

**Table 6:** Identification of experimentally validated gene targets of the miRNAs differentially expressed in MCF-7 cells transfected with HNRNPA2/B1.

Comparison time transfected with HNRNPA2/B1	Total DE miRNAs	Validated Gene Targets from miRTarBase
48 h vs. control	236	7859
72 h vs. control	349	8914
72 h vs. 48 h	433	10305

**Figure 5:**
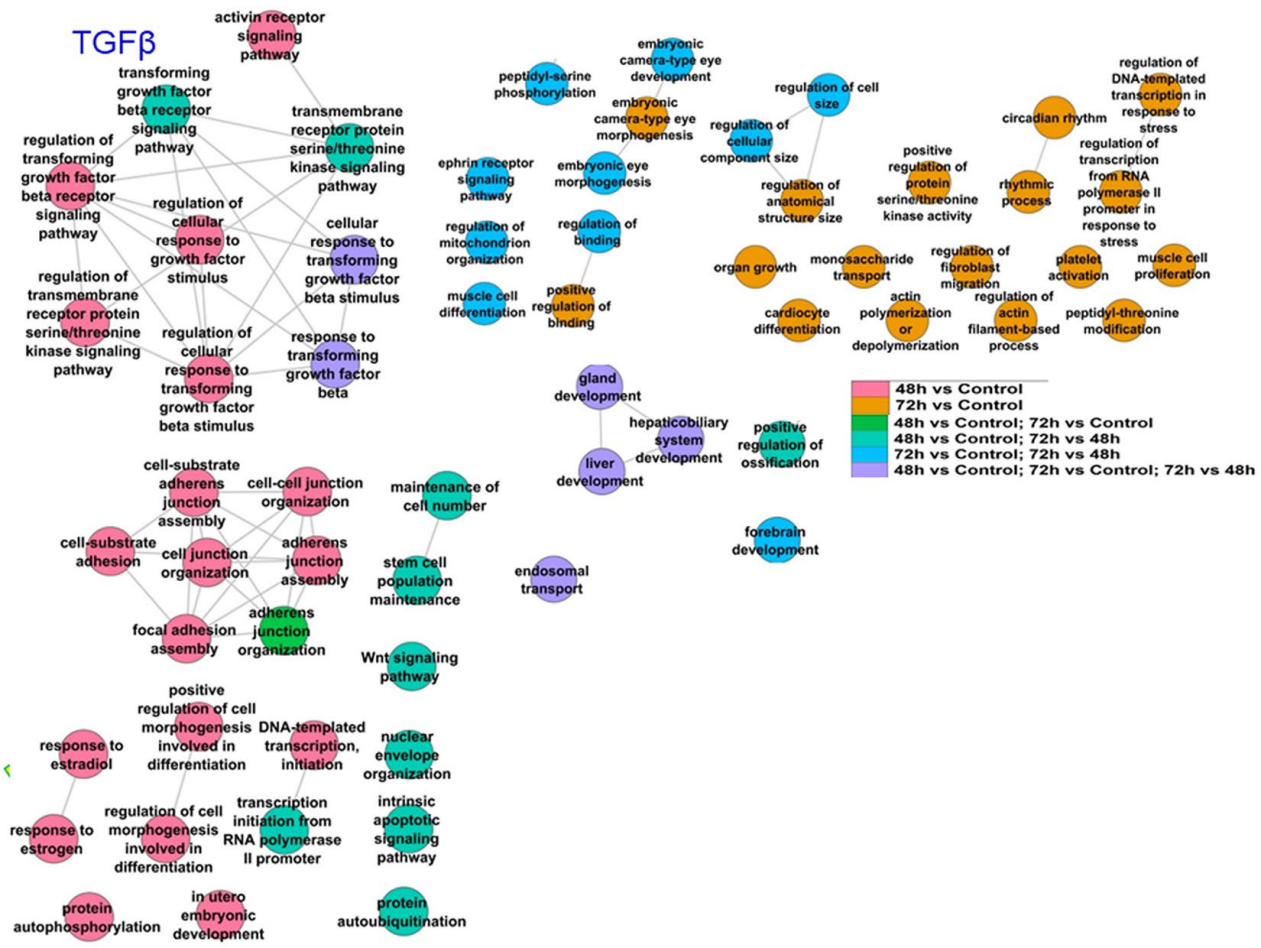
Enriched GO:BP (Gene Ontology: Biological Processes) for genes targeted by differentially expressed miRNAs at the indicated times. mRNA targets identified in miRTarBase as validated targets for the differentially expressed miRNAs at each time point were compared to control or the indicated comparison for GO:BP analysis in categoryCompare.

**Figure 6:**
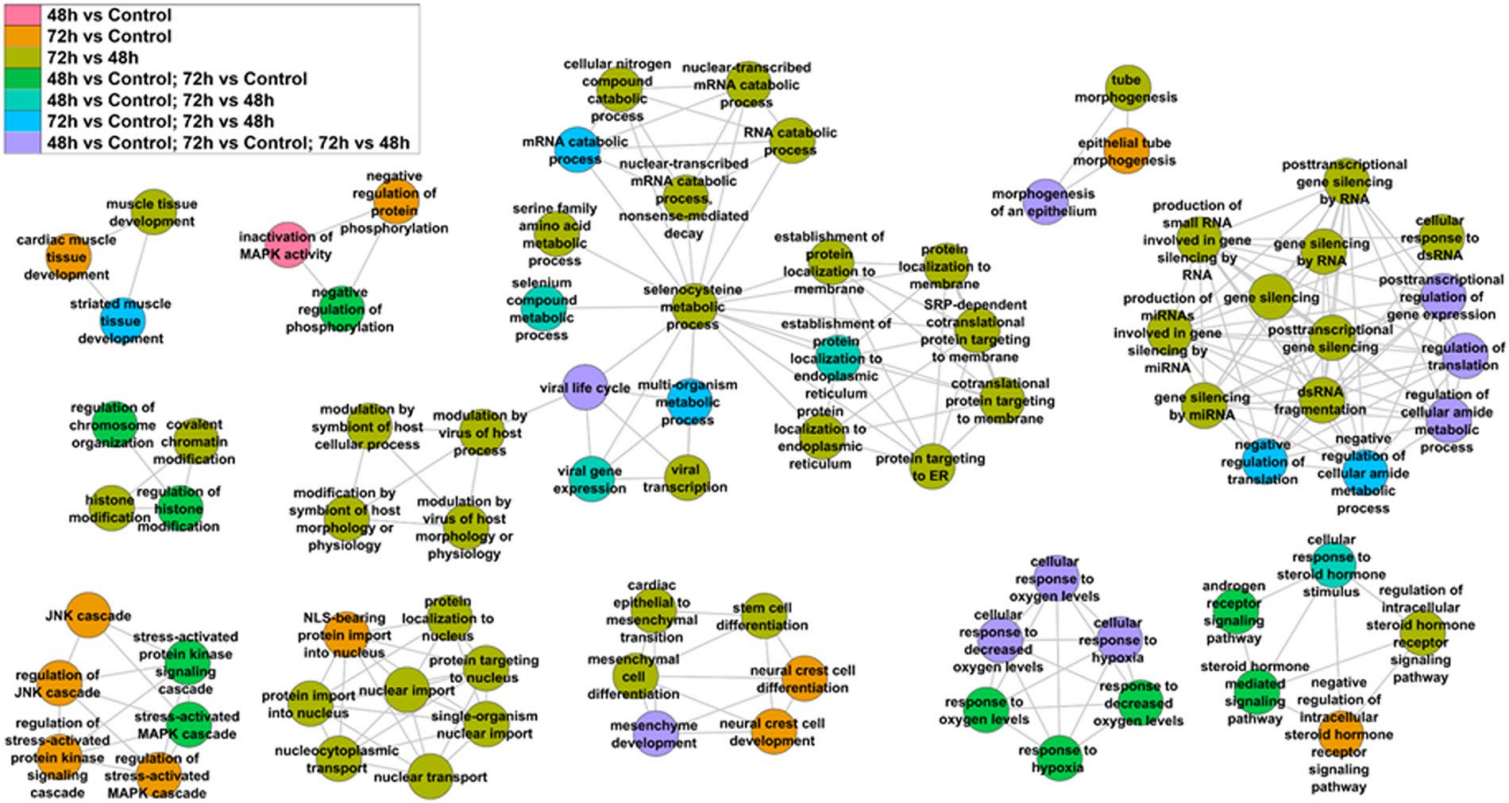
Enriched GO:BP (Gene Ontology: Biological Processes) for genes targeted by differentially expressed miRNAs at the indicated times. mRNA targets identified in miRTarBase as validated targets for the differentially expressed miRNAs at each time point were compared to control or the indicated comparison for GO:BP analysis in categoryCompare.

Processes putatively regulated by the HNRNPA2B1-regulated miRNAs in MCF-7 cells include TGFβ signaling (Fig. 5), which is protective in normal breast epithelium but acts as a tumor promoter after genetic and epigenetic changes involved in breast tumorigenesis accrue^45^. TGFβ induces EMT in breast cancer cells in a pathway involving tumor suppressor miR-34 family members and we observed miR-34c-5p was downregulated by HNRNPA2/B1 overexpression at 48 h (Supplementary Table 3). Other processes identified as downstream of HNRNPA2B1-regulated miRNAs included response to estrogen/estradiol, stem cell population maintenance, Wnt signaling, regulation of cell junction organization, cellular response to steroid hormone stimulus, JNK/MAPK cascade, and nuclear transport (Fig. 6). Future studies will address which targets in these pathways are downstream of HNRNPA2B1-regulation of miRNA expression and their role in endocrine-resistance.

### qPCR validation of HNRNPA2/B1-downregulated miRNA targets

**Figure 7:**
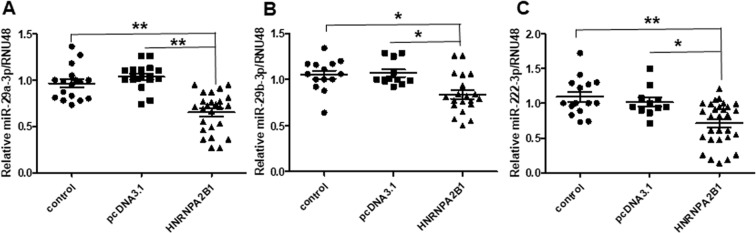
Regulation of miR-29a-3p, miR-29b-3p, and miR-222-3p by HNRNPA2B1. MCF-7 cells were either non-transfected (control), transfected with pcDNA3.1 parental vector, or an expression vector for HNRNPA2B1 for 48 h. qPCR for (**A)** miR-29a-3p; (**B)** miR-29b-3p, and (**C)** miR-222-3p. Each miRNA was normalized by RNU48. Values are relative expression normalized to control-transfected cells from 11 biological replicate experiments with multiple control and transfected wells in each experiment. Data were analyzed by two way ANOVA followed by Tukey’s *post hoc* test, *p < 0.05; **p < 0.01.

We selected miR-29a-3p, miR-29b-3p, and miR-222-3p for validation by qPCR based on their roles in breast cancer and responses to antiestrogen therapies^7,33,34,53–56^. Because 48 h HNRNPA2B1-transfected MCF-7 cells showed decreased expression of each of these miRNAs (Tables 4 and 5), we expected each miRNA to be decreased in new transient transfection experiments. Indeed, miR-29a-3p, miR-29b-3p, and miR-222-3p transcript expression was reduced by 48 h of HNRNPA2B1 transfection in MCF-7 cells, whereas transfection with the parental expression vector pcDNA3.1 had no significant effect (Fig. 7).

Both miR-29a and miR-29b are considered tumor suppressors in breast cancer and their repression results in in cancer stem cell expansion *in vitro*^57^. Progestins repress miR-29a and miR-29b in ER+/PR+ breast cancer cells^58^ and “PR action in breast cancer: stimulation of metastasis” was identified in MetaCore analysis. Patients with ductal carcinoma and elevated miR-29b levels had a significantly longer disease-free survival (DFS) and lower risk to relapse^58^. miR-29b-3p was downregulated in exosomes of patients with breast cancer recurrence, suggesting a role for miR-29b-3p in inhibition of breast cancer progression and recurrence^59^. Downregulation of miR-222-3p is associated with AI-resistance in long-term estrogen-deprived MCF-7 cells^60^. Since miR-222 represses TGFβ-stimulated breast cancer growth^56^, HNRNPA2B’s downregulation of miR-222-3p may facilitate TGFβ signaling as identified in the MetaCore analysis. Hence, downregulation of these three miRNAs by HNRNPA2B1 may be involved in development of a TAM-R phenotype and worse prognosis *in vivo*, although further experiments will be needed to determine the pathways and targets involved.

### qPCR validation of HNRNPA2/B1-upregulated miRNA targets

**Figure 8:**
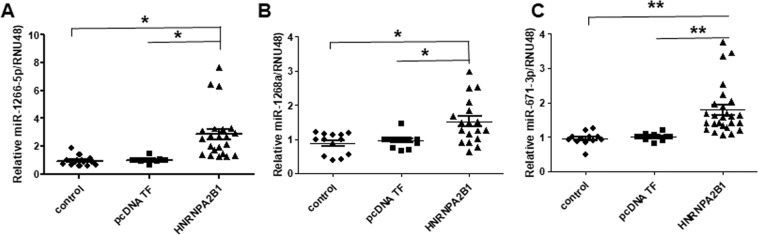
Regulation of miR-1266-5p, miR-1268a, and miR-671-3p by HNRNPA2B1. MCF-7 cells were either non-transfected (control), transfected with pcDNA3.1 parental vector (pcDNA), or an expression vector for HNRNPA2B1 for 48 h. qPCR for (**A)** miR-1266-5p; (**B)** miR-1268a, and (**C)** miR-671-3p. Each miRNA was normalized by RNU48. Values are relative expression normalized to control-transfected cells from 11 biological replicate experiments with multiple control and transfected wells in each experiment. Data were analyzed by two way ANOVA followed by Tukey’s *post hoc* test, *p < 0.01; **p < 0.001.

Based on their upregulation on HNRNPA2B1-transfected MCF-7 cells, we performed qPCR to validate increases in miR-1266-5p, miR-1268a, and miR-671-3p in separately HNRNPA2B1-transfected MCF-7 cells (11 biological replicate experiments, Fig. 8). As expected, all three miRNAs were significantly higher in HNRNPA2B1-transfected MCF-7 cells.

Expression of miR-1266 was increased in breast tumors showing recurrence or metastasis after TAM treatment with Kaplan-Meir analysis showing that higher miR-1266 was associated with shorter OS and DFS^61^. This suggests a role for increased miR-1266 in TAM-resistant breast cancer progression. miR-1268a is upregulated in drug-resistant MDA-MB-231 cell lines^62,63^. We observed and increased in miR-1268a in HNRNPA2B1-transfected MCF-7 cells, but whether this increase is associated with endocrine-resistance in ERα+ breast cancer cells remains to be evaluated.

miR-671-5p was identified as a tumor suppressor in breast cancer, as its expression is lower in invasive breast tumors compared with normal breast. It directly targets FOXM1, and miR-671-5p overexpression inhibits the proliferation and invasion of MDA-MB-231, Hs578T, and T47D, but not MCF-7 cells *in vitro*^64^. Likewise, miR-671-5p was downregulated in fulvestrant-resistant MCF-7 cells^43^. Thus, the increase in miR-671-5p with HNRNPA2B1 overexpression in MCF-7 cells might not have the same tumor suppressor properties. Because HNRNAP2B1 caused multiple changes in miRNA expression, it will be necessary to analyze combinations of miRNAs and examine both phenotypic and transcriptomic responses.

### Transient overexpression of HNRNPA2/B1 reduces TAM and fulvestrant responses in MCF-7 cells

**Figure 9:**
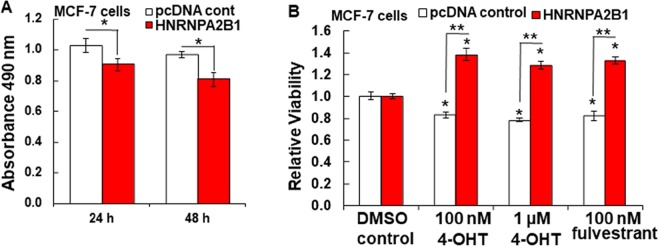
Effect of transient HNRNPA2B1 cells on MCF-7 cell viability. (**A)** Results are the Absorbance readings at 490 nm from an MTT assay in which 5,000 MCF-7 cells were plated in OPTI-MEM for 18 h prior to transfection with vector control (pcDNA cont) or HNRNPA2B1 for 24 or 48 h followed by an MTT assay. Each bar is the avg. ± SEM of 4 wells in one experiment. B) MCF-7 cells were transfected with the pcDNA control vector or HNRNPA2B1 for 24 and then treated with vehicle control (DMSO), 100 nM or 1 µM 4-OHT or 100 nM fulvestrant for 48 h followed by an MTT assay. The control was set to 1 for each transfection. Each bar is the avg. ± SEM of 4 wells in one experiment. *p < 0.05 vs. control in both A and B. In (**B**) **p < 0.05 vs. the same treatment between control vs. HNRNPA2B-transfected cells. Student’s 2-tailed t-test was used for analysis.

Since LCC9 TAM-resistant cells have higher HNRNPA2/B1 than parental, TAM-sensitive MCF-7 cells, we examined whether transient transfection of MCF-7 cells with HNRNPA2/B1 would impact cell viability in response to TAM or fulvestrant treatment. HNRNPA2B1 overexpression alone does not positively affect MCF-7 viability. We actually observed a 10–15% reduction in MCF-7 cell viability 24 and 48 h post- transfection (Fig. 9A). However, in response to 4-OHT or fulvestrant treatment, HNRNPA2/B1 overexpression was able to increase cell viability, indicating decreased sensitivity to ER antagonists (Fig. 9B). This response is similar to the increased viability of LCC9 cells treated with 4-OHT and fulvestrant^33^. These data suggest a role for increased HNRNPA2/B1 expression in endocrine-resistance in MCF-7 cells. Future experiments will be required to examine the precise pathways and the role of the altered miRNAs and their targets in this response. Additional future directions include examination of how HNRNPA2B1 overexpression in MCF-7 cells and knockdown in LCC9 cells affects phenotypes associated with TAM-resistance including cell invasion, migration, clonogenic survival, and examination of mRNA targets/proteins of the pathways identified, particularly TGFβ signaling.

## Conclusions

The primary goal of this study was to identify the global impact of HNRNPA2/B1 overexpression on the miRNA transcriptome of luminal A MCF-7 breast cancer cells, based on the observation of higher HNRNPA2/B1 in LCC9 endocrine-resistant breast cancer cells. We report the comprehensive miRNA changes after 48 and 72 h of HNRNPA2/B1 transfection. A limitation of this study is that both 48 and 72 h HNRNPAB1-transfected cells were compared to control-transfected MCF-7 cells at 48 h. Time- and direction-specific regulated miRNAs were characterized using the MetaCore GO enrichment analysis algorithm, and PR action in breast cancer and TGFβ signaling via miRNA in breast cancer were identified as pathways downstream of the HNRNA2B1 miRome in MCF-7 cells. HNRNPA2B1-downregualtion of miR-29a-3p, miR-29b-3p, and miR-222-3p were confirmed by qPCR in separate experiments. Each of these miRNAs has established roles in breast cancer, including the PR action and TGFβ signaling pathways that were identified in MetaCore analysis. Transient overexpression of HNRNPA2B1 in MCF-7 cells abrogated the ability of 4-OHT and fulvestrant to reduce cell growth. These data support a role for increased HNRNPA2B1 in processes contributing to endocrine-resistance in breast cancer.

## Methods

### Cell culture and treatments

MCF-7 cells were purchased from American Type Tissue Collection (ATCC, Manassas, VA, USA) and were used within 9 passages from ATCC. MCF-7 cells were grown as described previously^65^ prior to transient transfection with pcDNA3.1+C-DYK or pcDNA3.1+C-DYK into which HNRNPA2/B1 was cloned (purchased from GenScript, Piscataway, NJ, USA) using Lipofectamine 3000 (Invitrogen, Carlsbad, CA, USA) and Opti-MEM® Reduced Serum Medium (Thermo Fisher Scientific, Carlsbad, CA, USA). The medium was changed from OPTI-MEM (Thermo Fisher Scientific) to Modified IMEM (Thermo Fisher) + 10% FBS six hours after transfection. For the 72 h transfected cells, the medium was replaced with fresh medium 48 h post transfection. A total of six biological replicates for each sample were analysed: control, HNRNPA2/B1-transfected for 48 h, and HNRNPA2/B1-transfected for 72 h.

### For miRNA-seq

miRNA was isolated from six separate, biological replicate experiments for each sample group (Control, HNRNPA2/B1 48 h, HNRNPA2/B172 h) using the miRNeasy mini kit from Qiagen (Hilden, Germany) following the manufacturer’s directions. The integrity of the miRNA was confirmed using Agilent 2100 Bioanalyzer (Agilent Technologies, Santa Clara, CA). Libraries were prepared using QIAseq miRNA Library Kit (Qiagen). cDNA samples were barcoded with QIAseq miRNA NGS ILM IP primers. Adaptor dimers were removed from amplified libraries using QIAseq miRNA NGS beads. The quantity and quality of the library were analyzed on an Agilent Bioanalyzer using the Agilent high sensitivity DNA kit. Pooled library samples were run on an Illumina miSeq to test quantity and quality using the miSeq Reagent Nano Kit V2 300 cycles (Illumina, Foster City, CA). Library and PhIX control (Illumina, Cat. No. FC-110-3001) were denatured and diluted using the standard normalization method to a final concentration of 6 pM. 300 µl of library and 300 µl of PhIX were combined and sequenced on Illumina MiSeq. Based on the miSeq run, the concentration of the libraries was corrected and re-pooled. Sequencing was performed on the University of Louisville Center for Genetics and Molecular Medicine’s (CGeMM) Illumina NextSeq 500 using the NextSeq 500/550 75 cycle High Output Kit v2 (FC-404-2002). Seventy-two single-end raw sequencing files (.fastq) representing three conditions with six biological replicates and four lanes per replicate were downloaded from Illumina’s BaseSpace onto the KBRIN server for analysis. Data were analyzed using miRDeep2^66^ and edgR^67^.

The sequence reads were mapped to the human reference genome (hg19). Quality control (QC) of the raw sequence data was performed using FastQC (version 0.10.1)^68^. The FastQC results indicate quality trimming is not necessary since the minimum quality value for all samples is well above Q30 (1 in 1000 error rate). Preliminary adapter trimming was performed on each of the samples to remove the Qiagen 3′ Adapter sequence with Trimmomatric v0.33^69^. For all of the samples, a peak around 22 bp was found with a broader peak prior to 22 bp (data not shown). Further examination of the overrepresented full-length sequences show that many of these are from other ncRNA sequences. Furthermore, the mapping rate is similar among replicates of the same samples. Therefore, although the distributions differ, the resulting data appears to be consistent with previous miR reports^70^. The trimmed sequences were directly aligned to the human hg19 reference genome assembly using mirDeep2^66^. Supplementary Table 1 indicates the number of raw reads, number of reads after trimming, and number of reads successfully aligned for each of the samples. The aligned sequences were then used as inputs into mirSeep2 along with the mirBase^71^ release 22 mature miRNA and miRNA hairpin sequences. The result is a file containing the number of reads mapping to each of the 2,822 human (hsa) miRs. After quantification, the resulting counts for each miR in each sample were combined into a reads matrix. Using the counts table resulting from the previous step, differentially expressed miRs were determined using edgeR^67^. Using a p-value cutoff of 0.05, the number of differentially expressed miRs in each comparison is shown in Table 1. A heatmap was constructed for differentially expressed miRs passing a FC threshold of ±4 (Log2FC ± 2) in one or more of the comparisons (Fig. 1). The resulting heatmap is shown with up-regulated genes (treatment vs. control) in red and down-regulated genes in green (Fig. 5). The differentially expressed miRs are shown in Tables 1–4, Supplementary Tables 2, 3 for all comparisons. Several miRs are listed twice, due to their coding from multiple gene locations. The raw data were uploaded in the Gene Expression Omnibus (GEO) database as GSE122634.

### *In silco* identification of mRNA targets for miRNAs identified as HNRNPA2/B1-regulated in MCF-7 cells

Experimentally validated mRNA targets for human miRs were downloaded from miRTarBase release 6.1^72^ from http://mirtarbase.mbc.nctu.edu.tw/php/download.php which contains 410,620 miRNA-mRNA interacting pairs. The differentially expressed miRs were then searched against miRTarBase for gene targets. Table 5 shows the number of differentially expressed miRs and the number of validated targets for these miRs. Genes identified as targets of the DEG miRNAs were used as an input into categoryCompare [13] to determine enriched Gene Ontology Biological Processes (GO:BP) and KEGG pathways^52^ (Supplementary Fig. 8).

### *In silico* MetaCore network analysis

Pathway and network analysis of differentially expressed genes was performed in MetaCore version 6.27 (GeneGO, Thomson Reuters, New York, NY, USA).

### RNA extraction and quantitative real-time PCR (qPCR)

RNA was extracted using the RNeasy Mini Kit (Qiagen, Gaithersburg, MD, USA). For miRNA analysis, RNA was isolated using miRNeasy Mini Kit RNA (Qiagen). RNA concentration and quality was assessed using a NanoDrop spectrophotometer (Thermo Scientific, Rockford, IL, USA). The TaqMan® MicroRNA Reverse Transcription Kit and the High Capacity cDNA Reverse Transcription Kit for RNA (both from ThermoFisher) were used to make cDNA for miRNA and mRNA, respectively. Quantitative real-time PCR (qPCR) HNRNPA2/B1 was performed using TaqMan assays (ThermoFisher). 18S rRNA (ThermoFisher) was used as normalizer. qPCR for miR-29a-3p, miR-29b-3p, and miR-222-3p used TaqMan assays and were normalized to RNU6B (ThermoFisher). qPCR was performed using an ABI Viia 7 Real-Time PCR system (LifeTechnologies) with each reaction run in triplicate. The comparative threshold cycle (C_T_) method was used to determine ΔC_T_, ΔΔC_T_, and fold-change relative to control^73^.

### MTT assay

MCF-7 cells were transfected in 6-well plates for 24 h prior to counting and replating (5,000 cells/well) to 96-well plates in phenol red free IMEM supplemented with 5% charcoal-stripped fetal bovine serum (CSS-FBS, Atlanta Biologicals, Lawrenceville, GA, USA) and 1% penicillin/streptomycin (Invitrogen, Carlsbad, CA, USA). Cells were treated with vehicle control (DMS), 100 nM or 1 µM 4-OHT (4-hydroxytamoxifen, Sigma-Aldrich, St. Louis, MO, USA), or 100 nM fulvestrant (ICI 182,780; Tocris, Ellisville, MO, USA) for 48 h and cell viability quantified using CellTiter Aqueous One Solution Cell Proliferation Assay (Promega, Fitchburg, WI, USA).

### Western blot

Whole cell extracts (WCE) were prepared in RIPA buffer (Sigma-Aldrich) with added phosphatase and complete protease inhibitors (Roche, Indianapolis, IN, USA). Protein concentrations were determined using the Bio-Rad DC protein assay (Bio-Rad, Hercules, CA, USA). 40 or 45 µg of WCE protein were electrophoresed on 10% SDS-PAGE gels and electroblotted on PVDF membranes (Bio-Rad) for western blotting with the following antibodies: HNRNPA2B1 (B1 epitope-specific^32^) antibody # 18941 from IBL America (Minneapolis, MN USA); GAPDH cat.# sc-365062 (Santa Cruz Biotechnology, Dallas, TX, USA); β-actin (cat. # A5316, Sigma-Aldrich). Bands were visualized using a Bio Rad ChemiDoc MP imager and quantified by UN-SCAN-IT Graph Digitizer Software 7.1 (Silk Scientific, Orem, UT, USA).

### Statistics

Statistical analyses were performed using GraphPad Prism 5 (Graph Pad Software, Inc., San Diego, CA, USA). For data in which two samples were compared, Student’s two-tailed test was performed. For data in which more than two samples were compared, one way ANOVA followed by Tukey’s *post hoc* test was performed.

## Supplementary information

Manuscript with Tracker

Supplementary Tables and Figures

## Data Availability

Raw sequencing data files obtained from our analysis are available at GEO: accession number GSE122634. All data analyzed during this study are included in this published article (and its Supplementary Information Files).
