# Author Correction: HNRNPA2/B1 is upregulated in endocrine-resistant LCC9 breast cancer cells and alters the miRNA transcriptome when overexpressed in MCF-7 cells

**DOI:** 10.1038/s41598-021-87869-6

**Published:** 2021-04-23

**Authors:** Carolyn M. Klinge, Kellianne M. Piell, Christine Schaner Tooley, Eric C. Rouchka

**Affiliations:** 1 Department of Biochemistry & Molecular Genetics, University of Louisville School of Medicine, Louisville, KY 40292 USA; 2 Department of Biochemistry, Jacobs School of Medicine and Biomedical Sciences, State University of New York at Buffalo, Buffalo, NY 14203 USA; 3 Bioinformatics and Biomedical Computing Laboratory, Department of Computer Engineering and Computer Science, University of Louisville, Louisville, KY 40292 USA

Correction to: *Scientific Reports* 10.1038/s41598-019-45636-8, published online 01 July 2019

The original version of this article contained errors. The tracked changed manuscript was erroneously published as a Supplementary Information file. This Supplementary Information file has now been removed. In addition, all in-text citations to “Supplementary Table 8” have been removed, as Supplementary Table 8 does not exist.

The original article has been corrected.

