# Supplementary material for: HNRNPA2/B1 is upregulated in endocrine-resistant LCC9 breast cancer cells and alters the miRNA transcriptome when overexpressed in MCF-7 cells
Source: Sci Rep. 2019 Jul 1;9:9430. doi: 10.1038/s41598-019-45636-8 (PMC6603045; doi:10.1038/s41598-019-45636-8)
Supplement: Supplementary file 1 — Manuscript with Tracker [file 41598_2019_45636_MOESM1_ESM.docx]

HNRNPA2/B1 is upregulated in endocrine-resistant LCC9 breast cancer cells and alters the miRNA transcriptome when overexpressed in MCF-7 cells

Carolyn M. Klinge^1*^, Kellianne M. Piell^1^, Christine Schaner Tooley^2^, and Eric C. Rouchka^3^

^1^Department of Biochemistry & Molecular Genetics, University of Louisville School of Medicine; Louisville, KY 40292 USA

^2^Department of Biochemistry, Jacobs School of Medicine and Biomedical Sciences, State University of New York at Buffalo, Buffalo, NY 14203 USA

^3^Bioinformatics and Biomedical Computing Laboratory, Department of Computer Engineering and Computer Science, Louisville, KY 40292 USA

*Corresponding author: Carolyn M. Klinge, Department of Biochemistry & Molecular Genetics

University of Louisville School of Medicine, Louisville, KY 40292 USA. Telephone: 502-852-3668, FAX: 502-852-3659 Email: [carolyn.klinge@louisville.edu](mailto:carolyn.klinge@louisville.edu)

MicroRNAs are dysregulated in breast cancer. Heterogeneous Nuclear Ribonucleoprotein A2/B1 (HNRNPA2/B1) is a reader of the N(6)-methyladenosine (m6A) mark in primary-miRNAs (pri-miRNAs) and promotes DROSHA processing to precursor-miRNAs (pre-miRNAs). We examined the expression of writers, readers, and erasers of m6A and report that HNRNPA2/B1 expression is higher in tamoxifen-resistant LCC9 breast cancer cells as compared to parental, tamoxifen-sensitive MCF-7 cells. To examine how increased expression of HNRNPA2/B1 affects miRNA expression, HNRNPA2/B1 was transiently overexpressed (~ 5.4-fold) in MCF-7 cells for whole genome miRNA profiling (miRNA-seq). 148 and 88 miRNAs were up- and down-regulated, respectively, 48 h after transfection and 177 and 172 up- and down-regulated, respectively, 72 h after transfection. MetaCore Enrichment analysis identified progesterone receptor action and transforming growth factor β (TGFβ) signaling via miRNA in breast cancer as pathways downstream of the upregulated miRNAs and TGFβ signaling via SMADs and Notch signaling as pathways of the downregulated miRNAs. GO biological processes for mRNA targets of HNRNPA2/B1-regulated miRNAs included response to estradiol and cell-substrate adhesion. qPCR confirmed HNRNPA2B1 downregulation of miR-29a-3p, miR-29b-3p, and miR-222 and upregulation of miR-1266-5p, miR-1268a, miR-671-3p. Transient overexpression of HNRNPA2/B1 reduced MCF-7 sensitivity to 4-hydroxytamoxifen and fulvestrant, suggesting a role for HNRNPA2/B1 in endocrine-resistance.

**Keywords:** breast cancer, miRNA, N6-methyladenosine, miRNA-seq, endocrine resistance

**Introduction**

The majority of breast tumors (70%) express estrogen receptor α (ERα) which is successfully targeted by adjuvant therapies that increase overall survival ^1^. The current standard adjuvant treatments for patients with ERα+ breast cancer either inhibit ERα activity, *e.g*., tamoxifen (TAM) for premenopausal women, or block the conversion of androgens to estrogens by aromatase inhibitors (AIs), *e.g*., letrozole, in postmenopausal women ^2^. Unfortunately, endocrine therapies are limited by the development of acquired endocrine resistance in ~30-40% of initially responsive patients that can occur up to 30 years after primary therapy ^3,4^. A variety of mechanisms have been implicated in TAM-resistance (TAM-R) ^5,6^, including altered microRNA (miRNA) and long noncoding RNA (lncRNA) expression ^7-9^. Most miRNAs are transcribed, by RNA polymerase II, either as introns of host genes or as independent genes called primary (pri)-miRNAs ^10^. Pri-miRNAs are processed by the DROSHA-DGCR8 microprocessor complex to precursor (pre)-miRNAs prior to nuclear export ^11^. In the cytoplasm, the double stranded pre-miRNA is unwound by the DICER-TRBP complex to incorporate one strand of the miRNA (called the guide strand) into the RNA induced silencing (RISC) complex containing the catalytic Argonaut proteins, *e.g*., AGO2 ^12^. By basepairing with nucleotides in the 3’UTR of target genes within RISC, miRNAs can act as either oncomiRs by reducing protein levels of tumor suppressors or as tumor suppressors by decreasing oncogenic proteins in breast tumors ^7^. The processing of pri-miRNA transcripts is regulated in part by post-transcriptional modifications (PTMs) of pri-miRNA ^13^.

Next-generation sequencing (NGS) and mass spectrometry identified N(6)-methyladenosine (m6A) as the most common modification of mRNA and lncRNAs ^14,15^. m6A plays a role in pre-mRNA processing, alternative splicing, nuclear export, stability, and translation ^16,17^ by acting as a ‘conformational marker’ that induces sequence-dependent outcomes in RNA remodeling ^18^. A recent report also identified higher m6A in selected pri-miRNA sequences that corresponded with increased levels of the corresponding mature miRNA in MDA-MB-231 triple negative breast cancer (TNBC) cells ^13^.

m6A methylation is added by the RNA methyltransferase complex (WTAP, METTL3, METTL14, VIRMA, and RBM15), removed by the dioxygenases FTO and ALKBH5, and recognized by a variety of ‘readers’, including YTHDF1, YTHDF2, and HNRNPA2/B1 ^19-21^. METTL3 methylation of m6A on pri-miRNAs ^13^ and RNA-dependent interaction of HNRNPA2/B1 with DGCR8, a component of the DROSHA complex, stimulate processing of selected pri-miRNA-m6A to precursor miRNA (pre-miRNA) ^22^. HNRNPA2/B1 transcript expression is upregulated in breast tissue of postmenopausal parous women ^23^, but its role in the protective effect of early pregnancy on postmenopausal ERα+ breast cancer is unknown ^24^. HNRNPA2/B1 protein expression was higher in breast tumors compared to normal breast and knockdown of HNRNPA2/B1 inhibited the proliferation of MCF-7 and MDA-MB-231 breast cancer cells by causing S phase arrest and apoptosis ^25^.

HNRNPA2 and HNRNPB1 are two splice isoforms transcribed from the same locus but are traditionally treated as a single protein ^26^. HNRNPB1 is a lower abundance (~5%) N-terminal splice variant of the more highly expressed HNRNPA2 isoform and contains an additional 12 aa encoded by exon 2 ^27^. HNRNPA2/B1 share the remaining protein structure including an RNA-binding domain containing two RNA recognition motifs (RRMs) separated by a 15 aa linker and a C-terminal Gly-rich, low complexity region with a prion-like domain (PrLD), RGG box, and Py-motif including M9 nuclear localization signal ^28^. In addition to its recognition of m6A in pri-miRNA and role in RNA splicing and processing ^29^, HNRNPA2/B1 is involved in DNA repair ^30^ and genome stability ^31^.

In MCF-7 ERα+ breast cancer cells, enhanced cross-linking immunoprecipitation (eCLIP) using antibodies specific to HNRNPB1 alone or HNRNPA2/B1 in combination identified 1,472 transcripts bound by both HNRNPB1 and HNRNPA2/B1, 899 transcripts uniquely bound by HNRNPB1, and 479 transcripts uniquely bound by HNRNPA2/B1 ^32^. HNRNPB1 binding sites revealed a preference for 5’-AGGAAGG-3’ *versus* 5’-UGGGGA-3’ for HNRNPA2/B1 ^32^. HNRNPA2/B1 binding peaks were primarily in chromatin samples, consistent with HNRNPA2/B1 binding to nascent transcripts ^32^.

Here we identified HNRNPA2/B1 expression to be higher in LCC9 and LY2 endocrine-resistant cells compared to parental MCF-7 luminal A breast cancer cells. We used miRNA-seq to identify differences in miRNA transcripts in MCF-7 cells when HNRNPA2/B1 is overexpressed and evaluated the pathways and mRNA targets associated with each misregulated miRNA for relevance to breast cancer and endocrine resistance. Progesterone receptor (PR) action in breast cancer and TGFβ signaling via miRNA in breast cancer were identified as pathways downstream of the upregulated miRNAs, and TGFβ signaling via SMADs and activation of Notch signaling were identified as pathways downstream of the downregulated miRNAs. TGFβ signaling, response to estradiol, and cell-substrate adhesion were pathways associated with mRNA targets of the identified miRNAs. Accordingly, overexpression of HNRNPA2/B1 in MCF-7 cells reduced their sensitivity to 4-hydroxytamoxifen and fulvestrant, indicating that increased HNRNPA2/B1 plays a role in tamoxifen and fulvestrant resistant cell proliferation.

**RESULTS AND DISCUSSION**

***Expression of RNA writers, readers, and erasers in breast cancer cells***

TAM/fulvestrant-resistant LCC9 breast cancer cells have higher levels of expression of diverse miRNAs compared with parental, TAM-sensitive MCF-7 cells ^33^. To determine if there are differences in the expression of the genes encoding the readers, writers, and erasers of reversible m6A RNA modification ^19^ between MCF-7 and LCC9 cells, we examined the steady state transcript levels of m6A writers (*WTAP, METTL3,* and *METTL14*), readers (*YTHDF1, YTHDF2, YTHDF3*, and *HNRNPA2/B1*) and erasers (*FTO* and *ALKBH5*) in RNA-seq data from our previous RNA-seq study, GEO accession number GSE81620 ^34^ (Figure 1A). The expression of *METTL3* and *YTHDF1* transcripts was lower in LCC9 than MCF-7 cells whereas *WTAP, FTO*, *ALKBH5*, and *HNRNPA2/B1* were higher in LCC9 than MCF-7 cells. The possible role of the expression of *METTL3, YTHDF1, WTAP, FTO*, and *HNRNPA2/B1* transcripts in human breast tumors on overall survival was examined using the online tool Kaplan-Meier Plotter ^35^. There was no association of overall survival (OS) for breast cancer patients based on primary tumor expression of *METTL3, YTHDF1*, or *WTAP* (Supplementary Figure 1). Low expression of *FTO* was associated with lower OS (Supplementary Figure 2A). However, higher FTO nuclear staining was reported in ER-/PR-/HER2+ breast tumors ^36^. Patients with ER-/PR-/HER2+ breast tumors have ~ 40% lower disease-free survival compared to women with luminal A breast tumors ^37^. *HNRNPA2/B1* transcript expression was higher than any of the other genes examined in the m6A pathway (Figure 1B). HNRNPA2/B1 protein expression was also ~ 2.6-fold higher in LCC9 and LY2 cells than MCF-7 cells (Figure 1C, 1D, Supplementary Figure 3). Kaplan-Meier (K-M) survival analysis showed that higher expression of *HNRNPA2/B1* is associated with lower OS to ~ 150 months (Supplementary Figure 2B). After ~ 220 months, the black line denoting high HNRNPA2B expression indicates reduced survival for those 3 patients in the K-M plot (Supplementary Figure 2B). More data are needed to better understand whether low HNRNPA2B1 in the primary tumor predicts reduced OS after ~220 months. Thus, because of the high expression of *HNRNPA2B1* at the transcript and protein levels in LCC9 endocrine-resistant cells, its association with lower survival, and its role in increasing pri-miRNA processing ^22^, we selected HNRNPA2B1 for further study.

***miRNA-seq analysis of HNRNPA2/B1-regulated miRNAs in MCF-7 cells***

Based on our observation of higher HNRNPA2/B1 in LCC9 compared to MCF-7 cells, we hypothesized that the overexpression of HNRNPA2/B1 in LCC9 cells promotes processing of pri-miRNAs resulting in increased pre- and mature miRNAs that act on targets and pathways to promote endocrine resistance. We note that HNRNPA2/B1 upregulated miR-99a, miR-125b, and miR-149 in MDA-MB-231 TNBC cells ^22^, and we reported higher levels of miR-125b and miR-149, but not miR-99a, in LY2 endocrine resistant breast cancer cells as compared to MCF-7 cells in an earlier study ^38^. To evaluate the effect of increased HNRNPA2/B1 on mature miRNA expression in breast cancer, MCF-7 cells were transiently transfected with a control vector for 48 h or an expression vector for HNRNPA2/B1 for 48 or 72 h (Figure 2A). A limitation of this analysis was that a 72 h control-transfected group was not included. We did not detect differences in control gene (*GAPDH*) expression between 48 and 72 h control-transfected samples (Supplementary Figure 3E). However, complete RNA transcriptome analysis of the 72 h control-transfected MCF-7 cells would have been a better control for the 72 h HNRNPA2/B1-transfected cells.

The transfection resulted in average ~ 5 fold increase in HNRNPA2/B1 protein expression (Figure 2B, 2C). miRNA was isolated from six replicate experiments 48 or 72 h after HNRNPA2/B1 transfection for global changes in the miRNA transcriptome (miRome). Supplementary Table 1 shows a summary of the sequence analysis of the samples. A heatmap shows the relative consistency of miRNA expression changes in the replicate samples within each comparison and the changes between time after HNRNPA2/B1 transfection (Supplementary Figure 4).

Three pairwise comparisons were evaluated: 48 h *versus* control, 72 h *versus* control, and 72 h *versus* 48 h. In total, 795 miRNAs were differentially expressed (p ≤ 0.05). 210 (110 up and 100 down) common to both time points, 236 (148 up and 88 down) uniquely at 48 h, and 349 (177 up and 172 down) uniquely at 72 h (Table 1). The identities and values of differentially expressed miRs are shown in Supplementary Tables 2-7 for all comparisons. Note that several miRs are listed twice, due to their coding from multiple gene locations. A heatmap for differentially expressed miRs passing a fold change (FC) threshold of ± 4 (Log2FC ±2) in one or more of the comparisons is shown in Figure 2D.

***miRNAs upregulated in HNRNPA2/B1-transfected MCF-7 cells***

Based on previous reports that HNRNPA2B1 increases processing of pri-miRNA to pre-miRNA and mature miRNAs ^13,22^, we hypothesized that HNRNPA2/B1 overexpression would increase levels of miRNAs regulated by m6A in the respective pri-miRNA. We focus only on the miRNAs whose expression was significantly increased in response to HNRNPA2B1 transfection (Figure 3, Tables 2-3, Supplementary Table 8). Figure 3 shows that 148 and 177 miRNAs were uniquely increased at 48 and 72 h after HNRNPA2B1 transfection while 110 miRNAs were increased at both time points.

Fourteen miRNAs were increased by ≥ 2.0-log fold at both 48 and 72 h (Table 2). Of the six miRNAs on which publications were found, four (miR-1266, miR-2861, miR-7107-5p, and miR-762) have oncogenic, endocrine- or chemo-resistance activities in breast cancer (Table 2). Sixty miRNAs were increased at 48h, but not 72 h (Table 3, Supplementary Table 8). Of the twenty miRNAs with publications, seven had reported oncomiR functions and six had tumor suppressor functions. HNRNPA2/B1 transfection increased miR-222-5p, which is increased in TAM-R MCF-7 cells, and its role in TAM-R and targets, including *ESR1* (ERα) and cell cycle genes, *e.g*., *CDKN1B* (P27/KIP1) have been reviewed ^7,39^. While miR-595 has no established role in breast cancer, it has tumor promoter roles in papillary thyroid carcinoma (PTC) ^40^ and human glioblastoma (GBM) cells ^41^. However, miR-595 acts as a tumor suppressor in ovarian cancer ^42^; thus, its role in breast cancer remains to be determined.

Fifty-one miRNAs were increased at 72 h, but were not increased at 48 h (Supplementary Table 8). Of the 17 miRNAs with published information relevant to cancer, 3 miRNAs (miR-4763-3p, miR-4787-5p, and miR-4800-3p) were reported to be higher in fulvestrant-resistant MCF-7 *versus* TAM-resistant MCF-7 cells ^43^.

MetaCore analysis was performed on all three groups of upregulated miRNAs (common to 48 and 72 h, unique to 48 h, and unique to 72 h (Figure 3). Pathways identified included “PR action in breast cancer: stimulation of metastasis” (involving downregulation of miR-29a-3p) and “TGFβ signaling via miRNA in breast cancer” (involving downregulation of miR-21-5p, miR-200a-3p, miR-200a-5p, miR-200c-3p, miR-200c-5p, miR-200b-3p, upregulation of miR-181a-5p) (Figure 4). miR-200 family members are downregulated in breast cancer and in TAM-R breast cancer cell lines and tumors (reviewed in ^7,44^). The decrease in miR-200 family members would be expected to relieve repression of ZEB1/2 leading to repression of E-cadherin and EMT, an indicator of breast cancer progression and metastasis ^45^. The GO processes identified included “Cellular response to estrogen stimulus” (upregulation of miR-574-5p and miR-466) (Supplementary Figure 6, Supplementary Table 2). Increased serum of miR-574-5p is a marker of breast cancer ^46^.

***miRNAs downregulated in HNRNPA2/B1-transfected MCF-7 cells***

Unexepectedly, we identified 88, 172, and 100 miRNAs downregulated at 48 h, 72 h, and both time points, respectively (Supplementary Figure 5). This is the first identification of miRNAs downregulated in response to HNRNPA2/B1 overexpression. Of course, this could be a direct or indirect effect. Another HNRNP family member, HNRNPA1, can either promote or inhibit pri-miRNA processing, resulting in increased mature miR-18a ^47^ and reduced let-7a-1 in HeLa cells ^48^. We did not detect any significant increase in miR-18a nor a decrease in let-7a-1 in HNRNPA2/B1-transfected MCF-7 cells, implying that these two HNRNPs have different targets in different cells.

We focused on those downregulated ≥ 2.0-log fold: 57 at 48 h, 130 at 72 h, and 18 in common (Figure 4, Supplementary Table 7). Nine of the 18 common downregulated miRNAs had roles in breast cancer, including miR-221-3p and miR-222-3p (both target *ESR1*) and miR-515-5p and miR-516-5p, which are increased in TAM-R MCF-7 cells (Table 4). Of the 57 miRNAs downregulated at 48 h (Table 5), 26 have reported roles in breast cancer. Some have roles in TAM-R. let-7i and miR-489 are downregulated in TAM-resistant breast cancer cells and miR-101, miR-221, miR-222, and miR-515 are upregulated in TAM-resistant MCF-7 and other breast cancer cells ^7,39,49^. Both miR-29a-3p and miR-29b-3p which reduce TAM-resistant MCF-7 cell (LCC9 and LY2 cell lines) proliferation, migration, and colony formation ^33^ were downregulated by HNRNPA2/B1 overexpression.

MetaCore pathway analysis identified “TGFβ signaling via SMADs in breast cancer” for the common 18 downregulated miRNAs, as well as “PR action in breast cancer: stimulation of metastasis” in the 48 h and “Activation of Notch signaling in breast cancer” in the 72 h downregulated miRNAs (Figure 4). MetaCore enrichment analysis by GO processes identified “cellular response to estrogen stimulus (miR-206)” and “response to estrogen” (also miR-206) (Supplementary Figure 7). E_2_, and the ER-selective agonist PPT suppressed miR-206 expression in MCF-7 cells ^50^.

***Identification of experimentally validated gene targets of the miRNAs differentially expressed in MCF-7 cells transfected with HNRNPA2/B1****.*

The differentially expressed miRNAs were searched against miRTarBase^51^ for experimentally validated gene targets. Table 6 shows the number of differentially expressed miRNAs and the number of validated gene targets for these miRNAs. Genes identified as targets of the DE miRNAs were used as an input into categoryCompare^52^ to identify enriched Gene Ontology Biological Processes (GO:BP) ^52^.

Processes putatively regulated by the HNRNPA2B1-regulated miRNAs in MCF-7 cells include TGFβ signaling (Figure 5), which is protective in normal breast epithelium but acts as a tumor promoter after genetic and epigenetic changes involved in breast tumorigenesis accrue ^45^. TGFβ induces EMT in breast cancer cells in a pathway involving tumor suppressor miR-34 family members and we observed miR-34c-5p was downregulated by HNRNPA2/B1 overexpression at 48 h (Supplementary Table 3). Other processes identified as downstream of HNRNPA2B1-regulated miRNAs included response to estrogen/estradiol, stem cell population maintenance, Wnt signaling, regulation of cell junction organization, cellular response to steroid hormone stimulus, JNK/MAPK cascade, and nuclear transport (Figure 6). Future studies will address which targets in these pathways are downstream of HNRNPA2B1-regulation of miRNA expression and their role in endocrine-resistance.

***qPCR validation of HNRNPA2/B1-downregulated miRNA targets***

We selected miR-29a-3p, miR-29b-3p, and miR-222-3p for validation by qPCR based on their roles in breast cancer and responses to antiestrogen therapies ^7,33,34,53-56^. Because 48 h HNRNPA2B1-transfected MCF-7 cells showed decreased expression of each of these miRNAs (Tables 4 and 5), we expected each miRNA to be decreased in new transient transfection experiments. Indeed, miR-29a-3p, miR-29b-3p, and miR-222-3p transcript expression was reduced by 48 h of HNRNPA2B1 transfection in MCF-7 cells, whereas transfection with the parental expression vector pcDNA3.1 had no significant effect (Figure 7).

Both miR-29a and miR-29b are considered tumor suppressors in breast cancer and their repression results in in cancer stem cell expansion *in vitro* ^57^. Progestins repress miR-29a and miR-29b in ER+/PR+ breast cancer cells ^58^ and “PR action in breast cancer: stimulation of metastasis” was identified in MetaCore analysis. Patients with ductal carcinoma and elevated miR-29b levels had a significantly longer disease-free survival (DFS) and lower risk to relapse ^58^. miR-29b-3p was downregulated in exosomes of patients with breast cancer recurrence, suggesting a role for miR-29b-3p in inhibition of breast cancer progression and recurrence ^59^. Downregulation of miR-222-3p is associated with AI-resistance in long-term estrogen-deprived MCF-7 cells ^60^. Since miR-222 represses TGFβ-stimulated breast cancer growth ^56^, HNRNPA2B’s downregulation of miR-222-3p may facilitate TGFβ signaling as identified in the MetaCore analysis. Hence, downregulation of these three miRNAs by HNRNPA2B1 may be involved in development of a TAM-R phenotype and worse prognosis *in vivo*, although further experiments will be needed to determine the pathways and targets involved.

***qPCR validation of HNRNPA2/B1-upregulated miRNA targets***

Based on their upregulation on HNRNPA2B1-transfected MCF-7 cells, we performed qPCR to validate increases in miR-1266-5p, miR-1268a, and miR-671-3p in separately HNRNPA2B1-transfected MCF-7 cells (11 biological replicate experiments, Figure 8). As expected, all three miRNAs were significantly higher in HNRNPA2B1-transfected MCF-7 cells.

Expression of miR-1266 was increased in breast tumors showing recurrence or metastasis after TAM treatment with Kaplan-Meir analysis showing that higher miR-1266 was associated with shorter OS and DFS ^61^. This suggests a role for increased miR-1266 in TAM-resistant breast cancer progression. miR-1268a is upregulated in drug-resistant MDA-MB-231 cell lines ^62,63^. We observed and increased in miR-1268a in HNRNPA2B1-transfected MCF-7 cells, but whether this increase is associated with endocrine-resistance in ERα+ breast cancer cells remains to be evaluated.

miR-671-5p was identified as a tumor suppressor in breast cancer, as its expression is lower in invasive breast tumors compared with normal breast. It directly targets FOXM1, and miR-671-5p overexpression inhibits the proliferation and invasion of MDA-MB-231, Hs578T, and T47D, but not MCF-7 cells *in vitro* ^64^. Likewise, miR-671-5p was downregulated in fulvestrant-resistant MCF-7 cells ^43^. Thus, the increase in miR-671-5p with HNRNPA2B1 overexpression in MCF-7 cells might not have the same tumor suppressor properties. Because HNRNAP2B1 caused multiple changes in miRNA expression, it will be necessary to analyze combinations of miRNAs and examine both phenotypic and transcriptomic responses.

***Transient overexpression of HNRNPA2/B1 reduces TAM and fulvestrant responses in MCF-7 cells***

Since LCC9 TAM-resistant cells have higher HNRNPA2/B1 than parental, TAM-sensitive MCF-7 cells, we examined whether transient transfection of MCF-7 cells with HNRNPA2/B1 would impact cell viability in response to TAM or fulvestrant treatment. HNRNPA2B1 overexpression alone does not positively affect MCF-7 viability. We actually observed a 10-15% reduction in MCF-7 cell viability 24 and 48 h post- transfection (Figure 9A). However, in response to 4-OHT or fulvestrant treatment, HNRNPA2/B1 overexpression was able to increase cell viability, indicating decreased sensitivity to ER antagonists (Figure 9B). This response is similar to the increased viability of LCC9 cells treated with 4-OHT and fulvestrant ^33^. These data suggest a role for increased HNRNPA2/B1 expression in endocrine-resistance in MCF-7 cells. Future experiments will be required to examine the precise pathways and the role of the altered miRNAs and their targets in this response. Additional future directions include examination of how HNRNPA2B1 overexpression in MCF-7 cells and knockdown in LCC9 cells affects phenotypes associated with TAM-resistance including cell invasion, migration, clonogenic survival, and examination of mRNA targets/proteins of the pathways identified, particularly TGFβ signaling.

**Conclusions**: The primary goal of this study was to identify the global impact of HNRNPA2/B1 overexpression on the miRNA transcriptome of luminal A MCF-7 breast cancer cells, based on the observation of higher HNRNPA2/B1 in LCC9 endocrine-resistant breast cancer cells. We report the comprehensive miRNA changes after 48 and 72 h of HNRNPA2/B1 transfection. A limitation of this study is that both 48 and 72 h HNRNPAB1-transfected cells were compared to control-transfected MCF-7 cells at 48 h. Time- and direction-specific regulated miRNAs were characterized using the MetaCore GO enrichment analysis algorithm, and PR action in breast cancer and TGFβ signaling via miRNA in breast cancer were identified as pathways downstream of the HNRNA2B1 miRome in MCF-7 cells. HNRNPA2B1-downregualtion of miR-29a-3p, miR-29b-3p, and miR-222-3p were confirmed by qPCR in separate experiments. Each of these miRNAs has established roles in breast cancer, including the PR action and TGFβ signaling pathways that were identified in MetaCore analysis. Transient overexpression of HNRNPA2B1 in MCF-7 cells abrogated the ability of 4-OHT and fulvestrant to reduce cell growth. These data support a role for increased HNRNPA2B1 in processes contributing to endocrine-resistance in breast cancer.

**Methods**Cell culture and treatments: MCF-7 cells were purchased from American Type Tissue Collection (ATCC, Manassas, VA, USA) and were used within 9 passages from ATCC. MCF-7 cells were grown as described previously ^65^ prior to transient transfection with pcDNA3.1+C-DYK or pcDNA3.1+C-DYK into which HNRNPA2/B1 was cloned (purchased from GenScript, Piscataway, NJ, USA) using Lipofectamine 3000 (Invitrogen, Carlsbad, CA, USA) and Opti-MEM® Reduced Serum Medium (Thermo Fisher Scientific, Carlsbad, CA, USA). The medium was changed from OPTI-MEM (Thermo Fisher Scientific) to Modified IMEM (Thermo Fisher) + 10% FBS six hours after transfection. For the 72 h transfected cells, the medium was replaced with fresh medium 48 h post transfection. A total of six biological replicates for each sample were analysed: control, HNRNPA2/B1-transfected for 48 h, and HNRNPA2/B1-transfected for 72 h.

For miRNA-seq: miRNA was isolated from six separate, biological replicate experiments for each sample group (Control, HNRNPA2/B1 48 h, HNRNPA2/B172 h) using the miRNeasy mini kit from Qiagen (Hilden, Germany) following the manufacturer’s directions. The integrity of the miRNA was confirmed using Agilent 2100 Bioanalyzer (Agilent Technologies, Santa Clara, CA). Libraries were prepared using QIAseq miRNA Library Kit (Qiagen). cDNA samples were barcoded with QIAseq miRNA NGS ILM IP primers. Adaptor dimers were removed from amplified libraries using QIAseq miRNA NGS beads. The quantity and quality of the library were analyzed on an Agilent Bioanalyzer using the Agilent high sensitivity DNA kit. Pooled library samples were run on an Illumina miSeq to test quantity and quality using the miSeq Reagent Nano Kit V2 300 cycles (Illumina, Foster City, CA). Library and PhIX control (Illumina, Cat. No. FC-110-3001) were denatured and diluted using the standard normalization method to a final concentration of 6pM. 300 µl of library and 300 µl of PhIX were combined and sequenced on Illumina MiSeq. Based on the miSeq run, the concentration of the libraries was corrected and re-pooled. Sequencing was performed on the University of Louisville Center for Genetics and Molecular Medicine’s (CGeMM) Illumina NextSeq 500 using the NextSeq 500/550 75 cycle High Output Kit v2 (FC-404-2002). Seventy-two single-end raw sequencing files (.fastq) representing three conditions with six biological replicates and four lanes per replicate were downloaded from Illumina’s BaseSpace onto the KBRIN server for analysis. Data were analyzed using miRDeep2 ^66^ and edgR ^67^.

The sequence reads were mapped to the human reference genome (hg19). Quality control (QC) of the raw sequence data was performed using FastQC (version 0.10.1) ^68^. The FastQC results indicate quality trimming is not necessary since the minimum quality value for all samples is well above Q30 (1 in 1000 error rate). Preliminary adapter trimming was performed on each of the samples to remove the Qiagen 3’ Adapter sequence with Trimmomatric v0.33 ^69^. For all of the samples, a peak around 22 bp was found with a broader peak prior to 22 bp (data not shown). Further examination of the overrepresented full-length sequences show that many of these are from other ncRNA sequences. Furthermore, the mapping rate is similar among replicates of the same samples. Therefore, although the distributions differ, the resulting data appears to be consistent with previous miR reports ^70^. The trimmed sequences were directly aligned to the human hg19 reference genome assembly using mirDeep2 ^66^. Supplementary Table 1 indicates the number of raw reads, number of reads after trimming, and number of reads successfully aligned for each of the samples. The aligned sequences were then used as inputs into mirSeep2 along with the mirBase ^71^ release 22 mature miRNA and miRNA hairpin sequences. The result is a file containing the number of reads mapping to each of the 2,822 human (hsa) miRs. After quantification, the resulting counts for each miR in each sample were combined into a reads matrix. Using the counts table resulting from the previous step, differentially expressed miRs were determined using edgeR ^67^. Using a p-value cutoff of 0.05, the number of differentially expressed miRs in each comparison is shown in Table 1. A heatmap was constructed for differentially expressed miRs passing a FC threshold of ± 4 (Log2FC ±2) in one or more of the comparisons (Figure 1). The resulting heatmap is shown with up-regulated genes (treatment vs. control) in red and down-regulated genes in green (Figure 5). The differentially expressed miRs are shown in Tables 1-4, Supplementary Tables 2-3 for all comparisons. Several miRs are listed twice, due to their coding from multiple gene locations. The raw data were uploaded in the Gene Expression Omnibus (GEO) database as GSE122634.

*In silco* identification of mRNA targets for miRNAs identified as HNRNPA2/B1-regulated in MCF-7 cells: Experimentally validated mRNA targets for human miRs were downloaded from miRTarBase release 6.1 ^72^ from http://mirtarbase.mbc.nctu.edu.tw/ php/download.php which contains 410,620 miRNA-mRNA interacting pairs. The differentially expressed miRs were then searched against miRTarBase for gene targets. Table 5 shows the number of differentially expressed miRs and the number of validated targets for these miRs. Genes identified as targets of the DEG miRNAs were used as an input into categoryCompare [13] to determine enriched Gene Ontology Biological Processes (GO:BP) and KEGG pathways ^52^ (Supplementary Figure 8).

*In silico* MetaCore network analysis: Pathway and network analysis of differentially expressed genes was performed in MetaCore version 6.27 (GeneGO, Thomson Reuters, New York, NY, USA)

RNA extraction and quantitative real-time PCR (qPCR): RNA was extracted using the RNeasy Mini Kit (Qiagen, Gaithersburg, MD, USA). For miRNA analysis, RNA was isolated using miRNeasy Mini Kit RNA (Qiagen). RNA concentration and quality was assessed using a NanoDrop spectrophotometer (Thermo Scientific, Rockford, IL, USA). The TaqMan® MicroRNA Reverse Transcription Kit and the High Capacity cDNA Reverse Transcription Kit for RNA (both from ThermoFisher) were used to make cDNA for miRNA and mRNA, respectively. Quantitative real-time PCR (qPCR) HNRNPA2/B1 was performed using TaqMan assays (ThermoFisher). 18s rRNA (ThermoFisher) was used as normalizer. qPCR for miR-29a-3p, miR-29b-3p, and miR-222-3p used TaqMan assays and were normalized to RNU6B (ThermoFisher). qPCR was performed using an ABI Viia 7 Real-Time PCR system (LifeTechnologies) with each reaction run in triplicate. The comparative threshold cycle (C_T_) method was used to determine ΔC_T_, ΔΔC_T_, and fold-change relative to control ^73^.

MTT assay: MCF-7 cells were transfected in 6-well plates for 24 h prior to counting and replating (5,000 cells/well) to 96-well plates in phenol red freeIMEM supplemented with 5% charcoal-stripped fetal bovine serum (CSS-FBS, Atlanta Biologicals, Lawrenceville, GA, USA) and 1% penicillin/streptomycin (Invitrogen, Carlsbad, CA, USA). Cells were treated with vehicle control (DMS), 100 nM or 1 µM 4-OHT (4-hydroxytamoxifen, Sigma-Aldrich, St. Louis, MO, USA), or 100 nM fulvestrant (ICI 182,780; Tocris, Ellisville, MO, USA) for 48 h and cell viability quantified using CellTiter Aqueous One Solution Cell Proliferation Assay (Promega, Fitchburg, WI, USA).

Western blot: Whole cell extracts (WCE) were prepared in RIPA buffer (Sigma-Aldrich) with added phosphatase and complete protease inhibitors (Roche, Indianapolis, IN, USA). Protein concentrations were determined using the Bio-Rad DC protein assay (Bio-Rad, Hercules, CA, USA). 40 or 45 µg of WCE protein were electrophoresed on 10% SDS-PAGE gels and electroblotted on PVDF membranes (Bio-Rad) for western blotting with the following antibodies: HNRNPA2B1 (B1 epitope-specific ^32^) antibody # 18941 from IBL America (Minneapolis, MN USA); GAPDH cat.# sc-365062 (Santa Cruz Biotechnology, Dallas, TX, USA); β-actin (cat. # A5316, Sigma-Aldrich). Bands were visualized using a Bio Rad ChemiDoc MP imager and quantified by UN-SCAN-IT Graph Digitizer Software 7.1 (Silk Scientific, Orem, UT, USA).

Statistics: Statistical analyses were performed using GraphPad Prism 5 (Graph Pad Software, Inc., San Diego, CA, USA). For data in which two samples were compared, Student’s two-tailed test was performed. For data in which more than two samples were compared, one way ANOVA followed by Tukey’s *post hoc* test was performed.

**Acknowledgements:** This research was funded by a grant from NIH: R21CA21952 to C.M.K. and C.S.T. and by an internal grant to C.M.K. from the KBRIN Bioinformatics core that supported E.C.R. and is supported by NIH/NIGMS grant P20 GM103436 (Nigel Cooper, PI).

**Author Contributions**: C.M.K. and K.M.P performed experiments; C.M.K. designed experiments; E.C.R. performed bioinformatic analysis. C.M.K. performed MetaCore and statistical analyses; C.M.K. wrote the manuscript with editing by C.S.T. and E.C.R.

**Additional Information**

**Supplementary information** accompanies this paper at http/www.nature.com/srep

**Competing Interests:** The authors have no competing interests.

**Data availability statement:** Raw sequencing data files obtained from our analysis are available at GEO: accession number GSE122634. All data analyzed during this study are included in this published article (and its Supplementary Information files).

**Table 1: Comparison of the number of differentially expressed miRNAs using a p-value cutoff of ≤ 0.05.**

| **Comparison time transfected with HNRNPA2/B1** | **Total DE miRNAs** | **Upregulated miRNAs** | **Downregulated miRNA** |
| --- | --- | --- | --- |
| 48 h vs. control | 236 | 148 | 88 |
| 72 h vs. control | 349 | 177 | 172 |
| 72 h vs. 48 h | 433 | 204 | 229 |

**Table 2: Fourteen miRNAs were upregulated ≥ 2.0-fold by transient overexpression of HNRNPA2/B1 in MCF-7 cells at 48 and 72 h.** miRNAs are sorted by name. The log fold change (logFC) is the average of 6 biological replicate samples and all are statistically significant as indicated by the p ≤ 0.05. The apparent cellular role is based on publications cited related to breast or other cancers as indicated as found in PubMed and Google Scholar..

| **miRNA** | **logFC 48 h** | **logFC 72 h** | **Comments on role in breast or other cancers** | **Possible cellular role** |
| --- | --- | --- | --- | --- |
| miR-1266-3p | 2.77 | 2.94 | High breast tumor levels are a prognostic factor for recurrence-metastasis ^61^ | oncomiR |
| miR-2861 | 2.55 | 2.42 | Higher expression in fulvestrant-resistant MCF-7 cells ^43^. Increased in papillary thyroid carcinomas (PTC) with lymph node metastasis ^74^. | Endocrine-resistance oncomiR |
| miR-4426 | 3.63 | 3.63 | Downregulated in HER2-overexpressing MCF-7 cells ^75^. |  |
| miR-4667-5p | 2.25 | 2.25 | Upregulated in HER2-overexpressing MCF-7 cells ^75^ |  |
| miR-4739 | 3.03 | 3.03 | Increased by si-CTNNB1 (β-catenin) in gastric cancer (GC) cells, implying a tumor suppressive function in GC ^76^. | Tumor suppressor |
| miR-6087 | 2.15 | 2.03 | Downregulated in Adriamycin-resistant MCF-7 cells ^77^. |  |
| miR-6088 | 2.63 | 2.42 | Increased by the natural sweetener steviol in HCT-116 cells ^78^. |  |
| miR-6762-5p | 2.95 | 2.41 | No references found |  |
| miR-6771-5p | 2.49 | 2.26 | No references found |  |
| miR-6801-3p | 3.19 | 3.03 | No references found |  |
| miR-6803-5p | 2.45 | 2.00 | No references found |  |
| miR-6805-3p | 2.79 | 2.40 | Increased by 1 nM progestin R5020 in T47D, BT474, and ZR-75-1 BCa cells, but its role was not examined ^79^ |  |
| miR-7107-5p | 2.13 | 3.03 | Higher breast tumor expression levels associated with acquired resistance to chemotherapy ^63^. | oncomiR chemo-resistance |
| miR-762 | 3.23 | 2.41 | promotes BrCa cell proliferation & invasion by targeting IRF7 ^80^. Directly targets tumor suppressor MEN1 in ovarian cancer and promotes metastasis ^81^. | oncomiR |

**Table 3: Sixty miRNAs were upregulated ≥ 2.0-fold by transient overexpression of HNRNPA2/B1 in MCF-7 cells at 48 h, but not at 72 h.** The miRNAs are sorted by name. The logFC is the average of 6 biological replicate samples and all are statistically significant as indicated. The apparent cellular role is based on publications cited related to breast or other cancers as found in PubMed and Google Scholar. We found published information on 14 of these 60 miRNAs with 8 having oncomiR and 7 had tumor suppressor roles in breast or other cancer.

| **miRNA** | **logFC** | **Comments on role in breast or other cancers** | **Possible cellular role** |
| --- | --- | --- | --- |
| miR-1233-3p | 2.21 | Increased in serum of renal cell carcinoma (RCC) patients ^82^; represses *GDF15* ^83^ | oncomiR |
| miR-1279 | 2.55 | No references found |  |
| miR-1538 | 2.13 | Identified in serum of neuroblastoma patients ^84^. |  |
| miR-212-5p | 2.15 | overexpressed in drug-resistant breast tumors and DOX-resistant MCF-7 cells, targets *PTEN* ^85^ | oncomiR |
| miR-217 | 1.39 | Expression correlated with ER+ in breast tumors ^86^; higher in TNBC tumors than ER+ tumors, correlated with tumor grade and cancer stage and targeted *DACH1* ^87^; overexpression in MCF-7 cells reduced TAM-sensitivity, reduced E-cadherin, increased invasion and *SNAI1* (Snail), and downregulated PTEN ^88^; targets *PPARGC1A* (PGC-1α) in breast cancer cells ^89^; miR-217 inhibitor blocks docetaxel- or cisplatin-induced apoptosis in MCF-7 cells ^90^; acts as a tumor suppressor by targeting *KLF5* in TNBC cells ^91^. Directly targets *SIRT1* ^92^. | oncomiR  TAM-R  Tumor suppressor |
| miR-222-5p | 2.59 | Increased in TAM-R cells ^54^; Roles in BCa and TAM-R reviewed ^7,34,39^; high levels are associated with breast tumor stage ^93^. Directly targets *SSSCA1* (P27) ^94^. | oncomiR  TAM-R |
| miR-302c-3p | 2.60 | Expression correlated with HER2+ in breast tumors ^86^ and development of breast cancer ^95^; Directly targets *ESR1* (ERα) ^96^, *CCND1* (Cyclin D1) ^97^; *BCRP* ^98^; *MEKK1* ^99^ | oncomiR |
| miR-3129-3p | 2.57 | Downregulated in epithelial ovarian cancer (EOC) cell lines and overexpression by lentiviral transduction inhibited EOC cell proliferation *in vitro* and tumor xenograft growth *in vivo* ^100^. | Tumor suppressor in EOC |
| miR-3132 | 2.13 | Upregulated in A549, HUVEC and THP-1 cells infected with a hantavirus (Prospect Hill virus)^101^ |  |
| miR-3135a | 2.60 | No references relevant to human cancer were found |  |
| miR-3168 | 2.60 | Upregulated by 2 nM paclitaxel treatment in HepG2 cells and thought to play a role in paclitaxel resistance^102^ . |  |
| miR-3195 | 2.42 | Related to metastases ^103^ |  |
| miR-3610 | 2.58 | Upregulated in BCa tissues ^104^. |  |
| miR-3619-3p | 2.20 | High expression in MCF-7 cells and acts as a tumor suppressor by directly targeting *PLD* (phospholipase D) ^105^; higher expression correlated with tumor relapse in small cell carcinoma of the esophagus ^106^ | tumor suppressor |
| miR-3655 | 3.26 | Upregulated in metastatic melanoma ^107^. |  |
| miR-3674 | 2.40 | No references relevant to cancer were found |  |
| miR-3919 | 2.60 | No references relevant to cancer were found |  |
| miR-3923 | 2.13 | Downregulated in primary breast tumors with lymph node metastasis ^108^. | tumor suppressor |
| miR-3938 | 2.57 | No references relevant to cancer were found |  |
| miR-3944-5p | 2.00 | Upregulated by hypoxia in AC16 cardiomyocytes ^109^. |  |
| miR-3960 | 2.24 | No references found re. experimental evidence for miR-3960 in cancer. |  |
| miR-410-5p | 2.00 | Located in the DLK1-DIO3 genomic region 14q32 that has 2 maternally and 3 paternally expressed genes, 2 lncRNAs, and 53 miRNAs ^110^; high expression favorable in gastric, ovarian, and lung cancer ^110^; miR-410-3p is downregulated in breast tumors and acts as a tumor suppressor by targeting *SNAI1* ^111^; Suppresses cell growth, migration, and invasion and enhances apoptosis in MCF-7 and T47D cells; directly targets *ERLIN2* ^112^; Directly targets *STAT3* ^113^. | tumor suppressor |
| miR-4459 | 2.38 | Upregulated in exosomes derived from chemoresistant ovarian cancer cells ^114^. Identified as specific for ERBB2 breast tumors ^115^. |  |
| miR-4463 | 2.60 | Increased in serum of PCOS patients ^116^ |  |
| miR-4524b-5p | 3.29 | Increased in salivary glands from Sjögren syndrome patients ^117^. |  |
| miR-4532 | 2.07 | Increased expression in MCF-7 CSC-mammospheres- spheroid culture ^118^; Higher in fulvestrant-resistant MCF-7 cells ^43^ | oncomiR TAM-R |
| miR-4634 | 3.86 | Expression similar in serum from BC patients vs controls ^119^. |  |
| miR-4653-5p | 3.86 | None found, but miR-4653-**3p** was decreased in recurrent/metastatic lesions compared to the matched ER+/PR+ primary breast tumors ^120^ |  |
| miR-4657 | 2.13 | Downregulated in metformin-treated cholangiocarcinoma tumor cell lines ^121^. |  |
| miR-4665-5p | 2.60 | Upregulated by mechanical compression of cancer-associated fibroblasts (CAFs) from invasive ductal carcinomas ^122^. |  |
| miR-4679 | 2.13 | Upregulated in VEGF-overexpressing K562 leukemia cells ^123^. |  |
| miR-4701-3p | 2.21 | Downregulated in fulvestrant-resistant MCF-7 cells ^43^. Upregulated in plasma of PTC patients ^124^ |  |
| miR-4717-5p | 2.60 | miR-4717-3p was downregulated in the blood of 6 breast cancer patients ^125^. |  |
| miR-4723-3p | 2.60 | Downregulated in prostate tumors and acts as a tumor suppressor by targeting *ABL1* ^126^. | tumor suppressor in PCA |
| miR-4739 | 2.60 | Increased by si-CTNNB1 (β-catenin) in gastric cancer (GC) cells, implying a tumor suppressive function in GC ^76^. |  |
| miR-4750-5p | 3.45 | Computational studies identified a binding site for miR-4750-5p in TBC1D17 that has a role in breast cancer, but direct interaction was not verified ^127^. |  |
| miR-4752 | 2.60 | No references found |  |
| miR-4755-3p | 3.24 | No references relevant to breast or other cancers was found |  |
| miR-4763-5p | 2.60 | Increased in blood from multiple myeloma patients ^128^. |  |
| miR-4787-5p | 2.98 | Downregulated in human pancreatic ductal adenocarcinomas ^129^. Upregulated in plasma as a specific biomarker of lung squamous cell carcinoma ^130^. |  |
| miR-4800-3p | 4.20 | Upregulated in MDA-MB-231 and Hs578T TNBC cells compared to MCF-7 and SK-BR-3 cells ^131^. |  |
| miR-500b-3p | 2.45 | Higher in blood from patients with synovial sarcoma ^132^ and prostate cancer (PCA) ^133^ than controls. |  |
| miR-507 | 2.57 | Apparent tumor suppressor: lower in breast tumors and cell lines than non-neoplastic tissue and cells and directly targets *FLT1* ^134^. | tumor suppressor in BC |
| miR-5188 | 2.13 | Downregulated in siHER2-transfected BT474 cells ^75^ |  |
| miR-548f-3p | 2.35 | Upregulated in metformin-treated cholangiocarcinoma tumor cell lines ^121^. |  |
| miR-548g-3p | 2.39 | Directly targets the stem loop A promoter element within the 5′UTR of dengue virus and represses replication ^135^. |  |
| miR-5572 | 2.13 | Identified in minor salivary glands of Sjögren’s syndrome patients, in Jurkat cells, and in immortalized human salivary gland cell line pHSG^136^ |  |
| miR-5581-5p | 2.21 | Upregulated in vulvar squamous cell carcinomas ^137^ |  |
| miR-587 | 2.60 | Higher expression in chemoresistant colorectal tumors and modulates drug resistance by directly targeting PPP2R1B in colorectal cancer cells ^138^. | oncomiR in colo-rectal cancer |
| miR-595 | 2.66 | Commonly overexpressed in endocrine cancers, including PTC ^40^; tumor promoter in human glioblastoma (GBM) cells by directly targeting *SOX7* ^41^. Downregulated in ovarian cancer tissues and directly targets *ABCB1* ^42^. | oncomiR and tumor suppressor |
| miR-6075 | 2.75 | Increased expression in pancreato-biliary cancer ^139^ |  |
| miR-6501-5p | 2.41 | No references found |  |
| miR-6515-3p | 2.18 | Increased in blood from vitiligo patients^140^ |  |
| miR-6723-5p | 2.26 | Increased by xanthohumol (a hop plant extract that reduces cell viability) treatment of U87 MG glioma cells ^141^. |  |
| miR-6741-3p | 2.60 | Upregulated in the blood of Systemic Lupus Erythematous patients with class IV lupus nephritis ^142^. |  |
| miR-6762-5p | 2.95 | Identified as a potential target of hsa-circ-0036722, but not experimentally validated ^143^ |  |
| miR-6773-5p | 2.53 | Downregualted by cyclosporine A treatment of HK‐2 immortalized proximal tubule epithelial cells ^144^. |  |
| miR-6836-3p | 2.21 | Upregulated in MDA-MB-231 and Hs578T TNBC cells compared to MCF-7 and SK-BR-3 cells ^131^. |  |
| miR-6882-5p | 2.13 | Identified as a biomarker for pancreatic ductal adenocarcinoma ^145^ |  |
| miR-6886-3p | 2.58 | Downregulated by the lncRNA HULC in hepatocellular carcinoma (HCC) and directly targets *USP22* ^146^. |  |
| miR-7109-5p | 2.31 | No references found |  |
| miR-8079 | 2.13 | No references found |  |

**Table 4: Eighteen miRNAs were downregulated by transient overexpression of HNRNPA2/B1 in MCF-7 cells at 48 and 72 h.** miRNAs are sorted by name. LogFC is the average of 6 biological replicate samples and all are statistically significant as indicated by the p ≤ 0.05. The apparent cellular role is based on publications cited related to breast or other cancers as indicated as found in PubMed and Google Scholar.

| **miRNA** | **logFC** | **Comments on role in breast or other cancers** | **Possible Cellular role** |
| --- | --- | --- | --- |
| miR-1283 | -2.27 | *ATF1* rs11169571 variant C, associated with increased colorectal cancer risk, inhibits binding of miR-1283 ^147^. Downregulated in plasma from stage IV and stage III melanoma patients ^148^. Overexpressed in malignant Spitz lesions from melanoma tumors ^149^ |  |
| miR-221-3p | -1.16 | High expression in breast tumors vs. normal breast ^150^. Higher expression in TAM-resistant MCF-7 cells ^54^ and higher in MDA-MB-231 cells than MCF-7 cells ^151^. Targets *ESR1* ^152^, *BBC3* (PUMA) ^153^, TRPS1 ^154^, *BIM* ^155^, *NOTCH3* ^156^, *TNFAIP3* (A20) ^157^, PARP1 ^158^, and is involved in EMT ^159^ | oncomiR |
| miR-222-3p | -0.96 | High expression associates with high tumor stage, Ki-67 staining, luminal B, and HER2 amplification in breast tumors ^93^. Increased in TAM-R BCa cells and targets *ESR1*, *ERBB3* (reviewed in ^7^). | oncomiR |
| miR-224-5p | -2.86 | Higher expression in TNBC than in ER+/PR+/HER2 breast tumors ^160^. High expression in TNBC is associated with lower OS ^161^. Downregulated in aromatase-resistant BCa cells ^162^. *SMAD4* is a direct target ^163^. |  |
| miR-4458 | -2.34 | Deregulated in fulvestrant-resistant MCF-7 cells ^43^ |  |
| miR-4724-3p | -2.63 | No reports for breast or other cancers. |  |
| miR-4738-5p | -2.37 | No reports for breast or other cancers. |  |
| miR-486-5p | -1.24 | Downregulated in exosomes in serum from BCa patients with recurrence ^59^. | Tumor suppressor |
| miR-489-5p | -1.67 | Reduced in BCa tumors ^164^. Metastasis suppressor ^165^. reduced in TAM-resistant MCF-7 cells ^54^. | Tumor suppressor |
| miR-5008-3p | -2.63 | No reports for breast or other cancers. |  |
| miR-511-5p | -2.37 | No reports for breast or other solid cancers. |  |
| miR-515-5p | -3.57 | Suppressed by E_2_ in MCF-7 cells ^166^, E_2_-ERα-downregulated and TAM- ERα-upregulated; lower in ER- breast tumors and downregulates oncogenic genes in the WNT-signaling pathway ^167^. | Tumor suppressor |
| miR-516a-5p | -2.31 | Increased expression in TAM-R MCF-7 cells ^168^; miR-516a-5p targets *MARK4*, a regulator of the cytoskeleton and cell motility, in BCa ^169^. | Tumor suppressor |
| miR-518c-3p | -3.39 | No reports for breast or other cancers, but upregulated by E_2_ in MCF-7 cells ^170^. |  |
| miR-518d-5p | -2.37 | No reports for BCa. Downregulated by CircRNA8924 that acts as a competitive endogenous RNA of miR-51d-5p and miR-519-5p in cervical cancer ^171^. |  |
| miR-520c-5p | -2.37 | miR-520c is oncogenic in TNBC ^172^ | Oncogenic in TNBC |
| miR-526a | -2.37 | Transient overexpression of miR-526a mimics stimulated MCF-7 cell proliferation ^173^. |  |
| miR-6850-3p | -2.05 | No reports for breast or other cancers. |  |

**Table 5: Fifty-seven miRNAs were downregulated by transient overexpression of HNRNPA2/B1 in MCF-7 cells at 48 h.** miRNAs are sorted by name. LogFC is the average of 6 biological replicate samples and all are statistically significant as indicated by the p ≤ 0.05. The apparent cellular role is based on publications cited related to breast or other cancers as indicated as found in PubMed.

| **miRNA** | **logFC** | **Comments on role in breast or other cancers is information on breast cancer not identified in PubMed** | **Apparent Cellular role** |
| --- | --- | --- | --- |
| let-7f-2-3p | -0.75 | Low let-7f-2 predicts colon cancer progression ^174^; upregulated in renal cancer ^175^. |  |
| let-7i-3p | -0.76 | TAM-sensitivity of ZR-75-1 BCa cells was increased by let-7i transfection ^176^. |  |
| miR-100-5p | -2.90 | Down-regulated in CSC generated from MDA-MB-231 TNBC cells ^177^; miR-100 inhibits CSC in basal-like breast cancer and low miR-100 is a negative prognostic factor ^178^ |  |
| miR-101-3p | -0.61 | miR-101 transcripts on different chromosomes play diverse roles in the diagnosis, prognosis, and clinical outcome of BC ^179^. miR-101-1, closely linked to ER, PR, and HER2, is processed into miR-101-3p and miR-101-5p, while miR-101-2 lower in expression in BC tumors than normal breast tissue, only produces mature miR-101-3p ^179^.  *AMPK* is a verified direct target of miR-101-3p ^180^. Overexpression of miR-101 promotes E_2_-independent growth and TAM-R of MCF-7 cells ^181^. | Putative oncomiR in BCa (reviewed in ^7^).Tumor suppressor in breast cancer ^180^ |
| miR-101-5p | -1.09 | Downregulated in HCC tumors and is a “potential diagnostic marker” for HCC ^182^ | Putative tumor suppressor |
| miR-1251-5p | -1.36 | No reports for breast or other cancers. |  |
| miR-1323 | -3.60 | Higher expression in locally advanced esophageal squamous cell carcinoma tumors that are resistant to radiotherapy ^183^. Upregulated in radiation-resistant A549 NSCLC cells ^184^ and in radiation-resistant nasopharyngeal carcinoma cells^185^; High expression in cirrhosis-associated HCC correlated with “dismal survival and advanced staging” ^186^ | oncomiR |
| miR-134-3p | -2.37 | Suppresses ovarian cancer stem cell biogenesis by directly targeting *RAB27A* ^187^ | Tumor suppressor |
| miR-135a-5p | -2.30 | Reported to be highly expressed in breast tumors (n = 20) ^188^; Upregulated by E_2_ in MCF-7 cells ^189^; Directly targets *ESRRA* (ERRα) ^190^ and *ELK1* and *ELK3* oncogenes ^191^. | Tumor suppressor |
| miR-138-5p | -1.83 | Upregulated in the circulation of patients with breast cancer, but there was no change observed in the tumor tissue ^192^; Downregulated in breast tumors and lower expression was associated with advanced clinical tumor stage and metastatic disease ^193^; directly targets VIM (vimentin) and inhibits cell invasion, migration, and proliferation in BCa cell lines ^193^; | Tumor suppressor ^193^ |
| miR-145-5p | -1.85 | Induced by p53 and directly targets *MYC* ^194^ and *RPS6KB1* (P70S6K1) ^195^*.* Overexpression abrogates the oncogenic activity of circZNF609 in BCa cells; further, circZNF609 and miR-145-5p are strongly negatively correlated in breast tumors ^196^. linc01561 was a ceRNA of miR-145-5p in BCa cells ^197^. |  |
| miR-17-5p | -0.77 | Higher expression in TNBC *versus* luminal A invasive breast ductal carcinoma ^198^. Downregulates E_2_-ERα-regulated gene expression by downregulating coactivator *NCOA3* (AIB1, SRC-3) in MCF-7 cells ^199^. Directly targets *HBP1* to promote invasion and migration of BCa cells ^200^. Directly targets DR4 and DR5 to inhibit TRAIL-induced apoptosis in BCa cells ^201^. Suppresses TNBC cell proliferation and invasion by targeting *ETV1* ^202^. Downregulated in exosomes from BCa patients with recurrence ^59^. Directly inhibits translation of *NCOA3* (AIB1) in BCa cells ^199^ | Tumor suppressor ^203^; anti-metastatic function in basal breast tumors ^204^; oncomiR in other cancers ^205^ |
| miR-187-3p | -0.91 | Higher expression in sporadic BCa than in tumors from women with BRCA1 or BRCA2 mutations ^206^. Down-regulated in colorectal, prostate, lung, RCC, and HCC ^207^. |  |
| miR-193a-3p | -0.82 | Acts as a tumor suppressor by targeting *HIC2, HOXC9, PSEN1, LOXL4, ING5, c-KIT, PLAU,* *MCL,*  *SRSF2,* and *WT1* ^208^. Upregulated in fulvestrant-resistant MCF-7 cells ^43^. miR-193a gene is silenced by methylation and directly targets GRB7 in ovarian cancer ^209^. Only miR-193a-5p was downregulated in breast tumors whereas no difference was observed in the expression levels of miR-193-3p in BCa *versus* normal tissues ^210^. Both miR193a-5p and miR-193a-3p repressed MCF-7 and MDA-MB-231 cell proliferation by different targets. miR-193a-3p suppressed cell growth by inhibiting *CCND1, PLAU*, and *SEPN1* and inhibited cell motility by suppressing *PLAU* expression | Tumor suppressor in many cancers. |
| miR-19a-3p | -0.70 | Lower in MCF-7 than MDA-MB-231 cells ^211^. | oncomiR in breast cancer cells ^212^ |
| miR-19b-3p | -0.82 | Expression level was significantly down‐regulated in BCa vs normal breast ^213^. Downregulated in SK-BR-3 cells resistant to the PI3K inhibitor saracatinib and miR-19b-3p directly targets PIK3CA ^214^ | Tumor suppressor |
| miR-20a-5p | -0.64 | Higher in TNBC than luminal A invasive breast ductal cancer ^198^. LncRNA *HOTAIR* is overexpressed in breast tumors and directly binds downregulates miR-20a-5p ^215^. Directly targets *HMGA2* ^215^ and *RUNX3* ^216^. | Tumor suppressor |
| miR-26a-1-3p | -1.06 | Expression correlates with ER+/PR+ in breast tumors ^217^. Direct targets include *CCNE1, CDC2*, and *EZH2* ^217^; *CHD1, GREB1* and *KPNA2* ^218^, and *MCL1* ^219^. Downregulated by E_2_ in MCF-7 cells ^220^. Over-expression inhibited the growth of SKBR3 and BT474 cells ^221^ and of MDA-MB-231, MDA-MB-468, and MCF-7 cells ^222^. However, overexpression for ≥ 3 d results in aneuploidy in human BCa cells ^223^. |  |
| miR-29a-3p | -0.97 | stimulates migration and invasion; Repressed by MYC, YYI, NFκB, CEBPA and stimulated by p53 ^224^. | OncomiR and tumor suppressor |
| miR-29b-3p | -0.71 | Low expression in breast tumors correlates with reduced OS and DFS ^225^. Lower expression in invasive ductal adenocarcinoma *versu*s lobular carcinomas and elevated in ER+ *versus* ER- breast tumors ^58^.. Expression increased by GATA2 which suppresses *MMP9, ANGPTL4, VEGF*, and *LOX* promoting differentiation, blocking EMT to suppress metastasis ^226^. Regulated by S100A7 acting as an oncogene in ER-negative and as a cancer-suppressor in ER-positive BCa cells, with miR-29b being the determining regulatory factor ^227^. | Tumor suppressor |
| miR-3125 | -3.38 | No reports for BCa. Lower in glioblastoma tissues as a poor prognostic indicator ^228^. |  |
| miR-320e | -0.88 | No reports for breast cancer. Significantly higher expression in stage III colon cancers from patients with recurrence and associated with poorer DFS ^229^. |  |
| miR-34b-5p | -2.20 | Downregulated in breast tumors ^166,230^. | Tumor suppressor |
| miR-34c-5p | -2.36 | Downregulated in breast tumors ^231^ | Tumor suppressor |
| miR-3591-5p | -2.88 | No reports for BCa. Expression increased after radiation of A549 NSCLC cells and Ubiquitin Specific Peptidase 33 (USP33) was a downstream target of miR-3591-5p ^232^. |  |
| miR-3663-5p | -2.96 | No reports for BCa. Expression increased in human nonalcoholic fatty liver disease (NAFLD) ^233^. |  |
| miR-3912-3p | -0.72 | No reports for breast or other cancers. |  |
| miR-424-5p | -1.18 | Increased in serum of BCa patients with resistance to dovitinib ^234^. Low in basal-like breast tumors ^235^. | Tumor suppressor |
| miR-4419a | -2.37 | No reports for breast or other cancers. |  |
| miR-4500 | -2.08 | No reports for BCa, but down regulated in colorectal cancer and downregulates HMGA2 ^236^. |  |
| miR-4764-3p | -2.37 | Downregulated in HER2-overexpressing MCF-7 cells ^75^. |  |
| miR-4767 | -1.63 | No reports for breast or other cancers. |  |
| miR-4789-3p | -2.16 | Identified as specific for basal breast tumors ^115^. |  |
| miR-4790-3p | -1.66 | No reports for breast or other cancers. |  |
| miR-4793-3p | -2.37 | No reports for breast or other cancers. |  |
| miR-488-5p | -2.83 | Down regulated in breast tumors, but upregulated in plasma of patients with recurrent BCa ^237^. | Tumor suppressor |
| miR-497-3p | -0.66 | Higher in ER+ Breast tumors and directly targets *ERRA* ^238^. | Tumor suppressor |
| miR-520g-3p | -2.63 | miR-520g is higher in ER-/PR- breast tumors ^86^. Plasma miR-520g expression levels were significantly higher in BC patients with lymph node metastatic disease ^239^. | Oncogenic |
| miR-548ao-3p | -3.79 | Specifically upregulated in TNBC tumors ^240^ |  |
| miR-551b-3p | -1.99 | Lower in breast tumors and characterized as a tumor suppressor ^241^. | Tumor suppressor |
| miR-5584-5p | -2.63 | No reports for breast or other cancers. |  |
| miR-5681a | -2.07 | No reports for breast or other cancers. |  |
| miR-5682 | -2.37 | No reports for breast or other cancers. |  |
| miR-5692a | -3.51 | Overexpressed in HCC tumors and knockdown inhibited HCC cell growth and invasion ^242^. |  |
| miR-652-5p | -0.60 | Lower in breast tumors versus adjacent normal tissue ^243^. miR-652-3p levels were significantly lower in the serum of BCa patients than that in controls ^244^. | Tumor suppressor |
| miR-659-5p | -1.39 | Upregulated in rectal tumors from smokers ^245^. |  |
| miR-6716-3p | -1.61 | No reports for breast or other cancers. |  |
| miR-6733-3p | -1.74 | No reports for breast or other cancers. |  |
| miR-6794-3p | -2.57 | No reports for breast or other cancers. |  |
| miR-6795-3p | -2.90 | No reports for breast or other cancers. |  |
| miR-6834-5p | -2.37 | No reports for breast or other cancers. |  |
| miR-6878-5p | -2.37 | No reports for breast or other cancers. |  |
| miR-7975 | -2.00 | No reports for breast or other cancers. |  |
| miR-934 | -1.46 | Upregulated in breast tumors diagnosed ≤ 5.2 years postpartum in Hispanic women ^246^. |  |
| miR-937-3p | -2.17 | No reports for breast or other solid cancers. |  |
| miR-944 | -1.57 | Increased by E_2_ in MCF-7 cells ^189^. Higher in breast tumors and serum *versus* controls and targets *BNIP3* ^247^. However, another study reported that miR-944 expression was repressed in breast tumors and cell lines ^248^. miR-944 overexpression inhibited cell migration/invasion and directly targeted *SIAH1* and *PTP4A1* ^248^. miR-944 also inhibits metastasis of gastric cancer cells by inhibiting EMT by targeting *MACC1* ^249^. | Cisplatin-resistance  Tumor suppressors |
| miR-98-3p | -0.78 | Increased by E_2_ in MCF-7 cells ^250^. Downregulated in fulvestrant-resistant MCF-7 cells ^43^. Inhibition of endogenous miR-98 in 4T1 mouse BCa cells promoted cell proliferation, survival, tumor growth, invasion, and angiogenesis ^251^. miR-98 directly targets ALK4, MMP11, and HMGA2 ^252^. | Tumor suppressor |

**Table 6: Identification of experimentally validated gene targets of the miRNAs differentially expressed in MCF-7 cells transfected with HNRNPA2/B1.**

| **Comparison time transfected with HNRNPA2/B1** | **Total DE miRNAs** | **Validated Gene Targets from miRTarBase** |
| --- | --- | --- |
| 48 h vs. control | 236 | 7859 |
| 72 h vs. control | 349 | 8914 |
| 72 h vs. 48 h | 433 | 10305 |

**References Cited**

1 Zwart, W., Theodorou, V. & Carroll, J. S. Estrogen receptor-positive breast cancer: a multidisciplinary challenge. *Wiley Interdisciplinary Reviews: Systems Biology and Medicine* **3**, 216-230, doi:10.1002/wsbm.109 (2011).

2 Palmieri, C., Patten, D. K., Januszewski, A., Zucchini, G. & Howell, S. J. Breast cancer: Current and future endocrine therapies. *Mol. Cell. Endocrinol.* **382**, 695-723, doi:10.1016/j.mce.2013.08.001 (2014).

3 Ring, A. & Dowsett, M. Mechanisms of tamoxifen resistance. *Endocr Relat Cancer* **11**, 643-658, doi:10.1677/erc.1.00776 (2004).

4 Dowsett, M., Martin, L.-A., Smith, I. & Johnston, S. Mechanisms of resistance to aromatase inhibitors. *The Journal of Steroid Biochemistry and Molecular Biology* **95**, 167-172, doi:10.1016/j.jsbmb.2005.04.022 (2005).

5 Fan, P., Maximov, P. Y., Curpan, R. F., Abderrahman, B. & Jordan, V. C. The molecular, cellular and clinical consequences of targeting the estrogen receptor following estrogen deprivation therapy. *Mol. Cell. Endocrinol.* **408**, 245–263, doi:10.1016/j.mce.2015.06.004 (2015).

6 Clarke, R., Tyson, J. J. & Dixon, J. M. Endocrine resistance in breast cancer – An overview and update. *Mol. Cell. Endocrinol.* **418, Part 3**, 220-234, doi:10.1016/j.mce.2015.09.035 (2015).

7 Muluhngwi, P. & Klinge, C. M. Roles for miRNAs in endocrine resistance in breast cancer. *Endocrine-Related Cancer* **22**, R279-R300, doi:10.1530/erc-15-0355 (2015).

8 Egeland, N. G. *et al.* The Role of MicroRNAs as Predictors of Response to Tamoxifen Treatment in Breast Cancer Patients. *Int J Mol Sci* **16**, 24243-24275, doi:10.3390/ijms161024243 (2015).

9 Klinge, C. M. Non-Coding RNAs in Breast Cancer: Intracellular and Intercellular Communication. *Non-coding RNA* **4**, 40 (2018).

10 Saini, H. K., Griffiths-Jones, S. & Enright, A. J. Genomic analysis of human microRNA transcripts. *Proceedings of the National Academy of Sciences* **104**, 17719-17724, doi:10.1073/pnas.0703890104 (2007).

11 Daugaard, I. & Hansen, T. B. Biogenesis and Function of Ago-Associated RNAs. *Trends Genet.* **33**, 208-219, doi:10.1016/j.tig.2017.01.003 (2017).

12 Hock, J. & Meister, G. The Argonaute protein family. *Genome Biol* **9**, 210 (2008).

13 Alarcón, C. R., Lee, H., Goodarzi, H., Halberg, N. & Tavazoie, S. F. N6-methyladenosine marks primary microRNAs for processing. *Nature* **519**, 482-485, doi:10.1038/nature14281 (2015).

14 Pan, T. N6-methyl-adenosine modification in messenger and long non-coding RNA. *Trends Biochem. Sci.* **38**, 204-209, doi:10.1016/j.tibs.2012.12.006 (2013).

15 Fu, L. *et al.* Simultaneous Quantification of Methylated Cytidine and Adenosine in Cellular and Tissue RNA by Nano-Flow Liquid Chromatography-Tandem Mass Spectrometry Coupled with the Stable Isotope-Dilution Method. *Anal. Chem.* **87**, 7653-7659, doi:10.1021/acs.analchem.5b00951 (2015).

16 Dominissini, D. *et al.* Topology of the human and mouse m6A RNA methylomes revealed by m6A-seq. *Nature* **485**, 201-206, doi:10.1038/nature11112 (2012).

17 Meyer, K. D. & Jaffrey, S. R. The dynamic epitranscriptome: N6-methyladenosine and gene expression control. *Nat Rev Mol Cell Biol* **15**, 313-326, doi:10.1038/nrm3785 (2014).

18 Zou, S. *et al.* N6-Methyladenosine: a conformational marker that regulates the substrate specificity of human demethylases FTO and ALKBH5. *Scientific reports* **6**, 25677, doi:10.1038/srep25677 (2016).

19 Licht, K. & Jantsch, M. F. Rapid and dynamic transcriptome regulation by RNA editing and RNA modifications. *J. Cell Biol.* **213**, 15-22, doi:10.1083/jcb.201511041 (2016).

20 Wang, X. *et al.* N6-methyladenosine-dependent regulation of messenger RNA stability. *Nature* **505**, 117-120, doi:10.1038/nature12730 (2014).

21 Deng, X. *et al.* RNA N6-methyladenosine modification in cancers: current status and perspectives. *Cell Res.* **28**, 507-517, doi:10.1038/s41422-018-0034-6 (2018).

22 Alarcón, Claudio R. *et al.* HNRNPA2B1 Is a Mediator of m6A-Dependent Nuclear RNA Processing Events. *Cell* **162**, 1299-1308, doi:10.1016/j.cell.2015.08.011 (2015).

23 Peri, S. *et al.* Defining the genomic signature of the parous breast. *BMC Med Genomics* **5**, 46, doi:10.1186/1755-8794-5-46 (2012).

24 Barton, M., Santucci-Pereira, J. & Russo, J. Molecular pathways involved in pregnancy-induced prevention against breast cancer. *Front Endocrinol (Lausanne)* **5**, 213, doi:10.3389/fendo.2014.00213 (2014).

25 Hu, Y. *et al.* Splicing factor hnRNPA2B1 contributes to tumorigenic potential of breast cancer cells through STAT3 and ERK1/2 signaling pathway. *Tumour Biol.* **39**, 1010428317694318, doi:10.1177/1010428317694318 (2017).

26 Burd, C. G., Swanson, M. S., Görlach, M. & Dreyfuss, G. Primary structures of the heterogeneous nuclear ribonucleoprotein A2, B1, and C2 proteins: a diversity of RNA binding proteins is generated by small peptide inserts. *Proceedings of the National Academy of Sciences* **86**, 9788-9792, doi:10.1073/pnas.86.24.9788 (1989).

27 Kozu, T., Henrich, B. & Schäfer, K. P. Structure and expression of the gene (HNRPA2B1) encoding the human hnRNP protein A2/B1. *Genomics* **25**, 365-371, doi:10.1016/0888-7543(95)80035-K (1995).

28 Wu, B. *et al.* Molecular basis for the specific and multivariant recognitions of RNA substrates by human hnRNP A2/B1. *Nature Communications* **9**, 420, doi:10.1038/s41467-017-02770-z (2018).

29 Glisovic, T., Bachorik, J. L., Yong, J. & Dreyfuss, G. RNA-binding proteins and post-transcriptional gene regulation. *FEBS Lett.* **582**, 1977-1986, doi:doi:10.1016/j.febslet.2008.03.004 (2008).

30 Haley, B., Paunesku, T., Protic, M. & Woloschak, G. E. Response of heterogeneous ribonuclear proteins (hnRNP) to ionising radiation and their involvement in DNA damage repair. *Int. J. Radiat. Biol.* **85**, 643-655, doi:10.1080/09553000903009548 (2009).

31 Shkreta, L. & Chabot, B. The RNA Splicing Response to DNA Damage. *Biomolecules* **5**, doi:10.3390/biom5042935 (2015).

32 Nguyen, E. D., Balas, M. M., Griffin, A. M., Roberts, J. T. & Johnson, A. M. Global Profiling of hnRNP A2/B1-RNA Binding on Chromatin Highlights LncRNA Interactions. *RNA Biology* **15**, 91-913, doi:10.1080/15476286.2018.1474072 (2018).

33 Muluhngwi, P. *et al.* Tamoxifen differentially regulates miR-29b-1 and miR-29a expression depending on endocrine-sensitivity in breast cancer cells. *Cancer Lett.* **388**, 230-238, doi:10.1016/j.canlet.2016.12.007 (2017).

34 Muluhngwi, P., Alizadeh-Rad, N., Vittitow, S. L., Kalbfleisch, T. S. & Klinge, C. M. The miR-29 transcriptome in endocrine-sensitive and resistant breast cancer cells. *Scientific reports* **7**, 5205, doi:10.1038/s41598-017-05727-w (2017).

35 Gyorffy, B. *et al.* An online survival analysis tool to rapidly assess the effect of 22,277 genes on breast cancer prognosis using microarray data of 1,809 patients. *Breast Cancer Res. Treat.* **123**, 725-731, doi:10.1007/s10549-009-0674-9 (2010).

36 Tan, A., Dang, Y., Chen, G. & Mo, Z. Overexpression of the fat mass and obesity associated gene (FTO) in breast cancer and its clinical implications. *Int J Clin Exp Pathol* **8**, 13405-13410 (2015).

37 Prat, A. *et al.* Clinical implications of the intrinsic molecular subtypes of breast cancer. *The Breast* **24**, S26-S35, doi:10.1016/j.breast.2015.07.008 (2015).

38 Manavalan, T. T. *et al.* Differential expression of microRNA expression in tamoxifen-sensitive MCF-7 versus tamoxifen-resistant LY2 human breast cancer cells. *Cancer Lett.* **313**, 26-43, doi:10.1016/j.canlet.2011.08.018 (2011).

39 Klinge, C. M. miRNAs regulated by estrogens, tamoxifen, and endocrine disruptors and their downstream gene targets. *Mol. Cell. Endocrinol.* **418**, 273-297, doi:10.1016/j.mce.2015.01.035 (2015).

40 Lima, C. R., Gomes, C. C. & Santos, M. F. Role of microRNAs in endocrine cancer metastasis. *Mol. Cell. Endocrinol.* **456**, 62-75, doi:10.1016/j.mce.2017.03.015 (2017).

41 Hao, Y., Zhang, S., Sun, S., Zhu, J. & Xiao, Y. MiR-595 targeting regulation of SOX7 expression promoted cell proliferation of human glioblastoma. *Biomed. Pharmacother.* **80**, 121-126, doi:10.1016/j.biopha.2016.03.008 (2016).

42 Tian, S., Zhang, M., Chen, X., Liu, Y. & Lou, G. MicroRNA-595 sensitizes ovarian cancer cells to cisplatin by targeting ABCB1. *Oncotarget* **7**, 87091-87099, doi:10.18632/oncotarget.13526 (2016).

43 Zhou, Q. *et al.* Differential microRNA profiles between fulvestrant-resistant and tamoxifen-resistant human breast cancer cells. *Anticancer. Drugs* **29**, 539-548, doi:10.1097/cad.0000000000000623 (2018).

44 Klinge, C. M. Non-coding RNAs: long non-coding RNAs and microRNAs in endocrine-related cancers. *Endocrine-Related Cancer* **25**, R259-R282, doi:10.1530/erc-17-0548 (2018).

45 Gulei, D. *et al.* The “good-cop bad-cop” TGF-beta role in breast cancer modulated by non-coding RNAs. *Biochimica et Biophysica Acta (BBA) - General Subjects* **1861**, 1661-1675, doi:10.1016/j.bbagen.2017.04.007 (2017).

46 Huang, S. K. *et al.* A Panel of Serum Noncoding RNAs for the Diagnosis and Monitoring of Response to Therapy in Patients with Breast Cancer. *Med Sci Monit* **24**, 2476-2488, doi:oi: 10.12659/MSM.909453 (2018).

47 Guil, S. & Caceres, J. F. The multifunctional RNA-binding protein hnRNP A1 is required for processing of miR-18a. *Nat Struct Mol Biol* **14**, 591-596, doi:10.1038/nsmb1250 (2007).

48 Michlewski, G. & Cáceres, J. F. Post-transcriptional control of miRNA biogenesis. *RNA* **25**, 1-16, doi:10.1261/rna.068692.118 (2019).

49 Klinge, C. M. miRNAs and estrogen action. *Trends in Endocrinology & Metabolism* **23**, 223-233, doi:10.1016/j.tem.2012.03.002 (2012).

50 Adams, B. D., Furneaux, H. & White, B. A. The Micro-Ribonucleic Acid (miRNA) miR-206 Targets the Human Estrogen Receptor-{alpha} (ER{alpha}) and Represses ER{alpha} Messenger RNA and Protein Expression in Breast Cancer Cell Lines. *Mol. Endocrinol.* **21**, 1132-1147, doi:10.1210/me.2007-0022 (2007).

51 Chou, C.-H. *et al.* miRTarBase 2016: updates to the experimentally validated miRNA-target interactions database. *Nucleic Acids Res.*, doi:10.1093/nar/gkv1258 (2015).

52 Flight, R. M. *et al.* categoryCompare, an analytical tool based on feature annotations. *Front Genet* **5**, 98, doi:10.3389/fgene.2014.00098 (2014).

53 Schultz, D. J. *et al.* Transcriptomic response of breast cancer cells to anacardic acid. *Scientific reports* **8**, 8063, doi:10.1038/s41598-018-26429-x (2018).

54 Miller, T. E. *et al.* MicroRNA-221/222 confers tamoxifen resistance in breast cancer by targeting p27(Kip1). *J. Biol. Chem.* **283**, 29897-29903, doi:10.1074/jbc.M804612200 (2008).

55 Di Leva, G. *et al.* MicroRNA Cluster 221-222 and Estrogen Receptor {alpha} Interactions in Breast Cancer. *J. Natl. Cancer Inst.* **102**, 706-721, doi:10.1093/jnci/djq102 (2010).

56 Rao, X. *et al.* MicroRNA-221/222 confers breast cancer fulvestrant resistance by regulating multiple signaling pathways. *Oncogene* **30**, 1082-1097, doi:<http://www.nature.com/onc/journal/v30/n9/suppinfo/onc2010487s1.html> (2011).

57 Cittelly, D. M. *et al.* Progestin suppression of miR-29 potentiates dedifferentiation of breast cancer cells via KLF4. *Oncogene* **32**, 2555-2564, doi:10.1038/onc.2012.275 (2013).

58 Papachristopoulou, G., Papadopoulos, E. I., Nonni, A., Rassidakis, G. Z. & Scorilas, A. Expression Analysis of miR-29b in Malignant and Benign Breast Tumors: A Promising Prognostic Biomarker for Invasive Ductal Carcinoma With a Possible Histotype-Related Expression Status. *Clinical Breast Cancer* **18**, 305-312.e303, doi:10.1016/j.clbc.2017.11.007 (2018).

59 Sueta, A. *et al.* Differential expression of exosomal miRNAs between breast cancer patients with and without recurrence. *Oncotarget* **8**, 69934-69944, doi:10.18632/oncotarget.19482 (2017).

60 Hoppe, R. *et al.* Profiles of miRNAs matched to biology in aromatase inhibitor resistant breast cancer. *Oncotarget* **7**, 71235-71254, doi:10.18632/oncotarget.12103 (2016).

61 Sevinc, E. D. *et al.* Association of miR-1266 with recurrence/metastasis potential in estrogen receptor positive breast cancer patients. *Asian Pacific journal of cancer prevention : APJCP* **16**, 291-297 (2015).

62 Zhong, S. *et al.* MicroRNA expression profiles of drug-resistance breast cancer cells and their exosomes. *Oncotarget* **7**, 19601-19609, doi:10.18632/oncotarget.7481 (2016).

63 Chen, X. *et al.* The role of miRNAs in drug resistance and prognosis of breast cancer formalin-fixed paraffin-embedded tissues. *Gene* **595**, 221-226, doi:10.1016/j.gene.2016.10.015 (2016).

64 Tan, X. *et al.* miR-671-5p inhibits epithelial-to-mesenchymal transition by downregulating FOXM1 expression in breast cancer. *Oncotarget* **7**, 293-307, doi:10.18632/oncotarget.6344 (2016).

65 Schultz, D. J. *et al.* Genome-wide miRNA response to anacardic acid in breast cancer cells. *PLOS ONE* **12**, e0184471, doi:10.1371/journal.pone.0184471 (2017).

66 Friedlander, M. R., Mackowiak, S. D., Li, N., Chen, W. & Rajewsky, N. miRDeep2 accurately identifies known and hundreds of novel microRNA genes in seven animal clades. *Nucleic Acids Res* **40**, 37-52, doi:10.1093/nar/gkr688 (2012).

67 Robinson, M. D., McCarthy, D. J. & Smyth, G. K. edgeR: a Bioconductor package for differential expression analysis of digital gene expression data. *Bioinformatics* **26**, 139-140, doi:10.1093/bioinformatics/btp616 (2010).

68 Andrews, S. *FastQC: A Quality Control Tool for High Throughput Sequence Data*, <<http://bioinformatics.babraham.ac.uk/projects/fastqc/>> (2014).

69 Bolger, A. M., Lohse, M. & Usadel, B. Trimmomatic: a flexible trimmer for Illumina sequence data. *Bioinformatics* **30**, 2114-2120, doi:10.1093/bioinformatics/btu170 (2014).

70 Metpally, R. P. *et al.* Comparison of Analysis Tools for miRNA High Throughput Sequencing Using Nerve Crush as a Model. *Front Genet* **4**, 20, doi:10.3389/fgene.2013.00020 (2013).

71 Griffiths-Jones, S., Saini, H. K., van Dongen, S. & Enright, A. J. miRBase: tools for microRNA genomics. *Nucl. Acids Res.* **36**, D154-158, doi:10.1093/nar/gkm952 (2008).

72 Hsu, S.-D. *et al.* miRTarBase update 2014: an information resource for experimentally validated miRNA-target interactions. *Nucleic Acids Res.* **42**, D78-D85, doi:10.1093/nar/gkt1266 (2014).

73 Schmittgen, T. D. & Livak, K. J. Analyzing real-time PCR data by the comparative C(T) method. *Nat Protoc* **3**, 1101-1108 (2008).

74 Wang, Z. *et al.* Upregulation of miR-2861 and miR-451 expression in papillary thyroid carcinoma with lymph node metastasis. *Medical oncology (Northwood, London, England)* **30**, 577, doi:10.1007/s12032-013-0577-9 (2013).

75 Wang, X. *et al.* Differential expression profile analysis of miRNAs with HER-2 overexpression and intervention in breast cancer cells. *Int. J. Clin. Exp. Pathol.* **10**, 5039-5062 (2017).

76 Dong, L. *et al.* Interference with the beta-catenin gene in gastric cancer induces changes to the miRNA expression profile. *Tumour Biol.* **36**, 6973-6983, doi:10.1007/s13277-015-3415-1 (2015).

77 Feng, F. *et al.* Downregulation of hypermethylated in cancer-1 by miR-4532 promotes adriamycin resistance in breast cancer cells. *Cancer Cell International* **18**, 127, doi:10.1186/s12935-018-0616-x (2018).

78 Chen, J. *et al.* Steviol, a natural product inhibits proliferation of the gastrointestinal cancer cells intensively. *Oncotarget* **9**, 26299-26308, doi:10.18632/oncotarget.25233 (2018).

79 McFall, T. *et al.* Progesterone receptor A promotes invasiveness and metastasis of luminal breast cancer by suppressing regulation of critical microRNAs by estrogen. *J. Biol. Chem.* **293**, 1163-1177, doi:10.1074/jbc.M117.812438 (2018).

80 Li, Y. *et al.* microRNA-762 promotes breast cancer cell proliferation and invasion by targeting IRF7 expression. *Cell Prolif.* **48**, 643-649, doi:10.1111/cpr.12223 (2015).

81 Hou, R. *et al.* miR-762 can negatively regulate menin in ovarian cancer. *OncoTargets and therapy* **10**, 2127-2137, doi:10.2147/ott.S127872 (2017).

82 Wulfken, L. M. *et al.* MicroRNAs in Renal Cell Carcinoma: Diagnostic Implications of Serum miR-1233 Levels. *PLOS ONE* **6**, e25787, doi:10.1371/journal.pone.0025787 (2011).

83 Teng, M.-S. *et al.* A GDF15 3′ UTR variant, rs1054564, results in allele-specific translational repression of GDF15 by hsa-miR-1233-3p. *PLOS ONE* **12**, e0183187, doi:10.1371/journal.pone.0183187 (2017).

84 de Carvalho, I. N., de Freitas, R. M. & Vargas, F. R. Translating microRNAs into biomarkers: What is new for pediatric cancer? *Medical oncology (Northwood, London, England)* **33**, 49, doi:10.1007/s12032-016-0766-4 (2016).

85 Xie, M. *et al.* MicroRNA-132 and microRNA-212 mediate doxorubicin resistance by down-regulating the PTEN-AKT/NF-κB signaling pathway in breast cancer. *Biomed. Pharmacother.* **102**, 286-294, doi:10.1016/j.biopha.2018.03.088 (2018).

86 Lowery, A. *et al.* MicroRNA signatures predict oestrogen receptor, progesterone receptor and HER2/neu receptor status in breast cancer. *Breast Cancer Research* **11**, R27 (2009).

87 Zhang, Q., Yuan, Y., Cui, J., Xiao, T. & Jiang, D. MiR-217 Promotes Tumor Proliferation in Breast Cancer via Targeting DACH1. *J Cancer* **6**, 184-191, doi:10.7150/jca.10822 (2015).

88 Zhang, A. X. *et al.* MicroRNA-217 overexpression induces drug resistance and invasion of breast cancer cells by targeting PTEN signaling. *Cell Biol. Int.*, doi:10.1002/cbin.10506 (2015).

89 Zhang, S. *et al.* PGC-1 alpha interacts with microRNA-217 to functionally regulate breast cancer cell proliferation. *Biomed. Pharmacother.* **85**, 541-548, doi:10.1016/j.biopha.2016.11.062 (2017).

90 Kim, H., Kim, Y. & Jeoung, D. DDX53 Promotes Cancer Stem Cell-Like Properties and Autophagy. *Mol. Cells* **40**, 54-65, doi:10.14348/molcells.2017.2258 (2017).

91 Zhou, W. *et al.* miR-217 inhibits triple-negative breast cancer cell growth, migration, and invasion through targeting KLF5. *PLOS ONE* **12**, e0176395, doi:10.1371/journal.pone.0176395 (2017).

92 Deng, S. *et al.* Chronic pancreatitis and pancreatic cancer demonstrate active epithelial–mesenchymal transition profile, regulated by miR-217-SIRT1 pathway. *Cancer Lett.* **355**, 184-191, doi:10.1016/j.canlet.2014.08.007 (2014).

93 Han, S. H. *et al.* MicroRNA-222 Expression as a Predictive Marker for Tumor Progression in Hormone Receptor-Positive Breast Cancer. *Journal of breast cancer* **20**, 35-44, doi:10.4048/jbc.2017.20.1.35 (2017).

94 Wang, D.-d. *et al.* miR-222 confers the resistance of breast cancer cells to Adriamycin through suppression of p27kip1 expression. *Gene* **590**, 44-50, doi:10.1016/j.gene.2016.06.013 (2016).

95 Chen, D. & Yang, H. Integrated analysis of differentially expressed genes in breast cancer pathogenesis. *Oncology letters* **9**, 2560-2566, doi:10.3892/ol.2015.3147 (2015).

96 Leivonen, S. K. *et al.* Protein lysate microarray analysis to identify microRNAs regulating estrogen receptor signaling in breast cancer cell lines. *Oncogene* **28**, 3926-3936, doi:10.1038/onc.2009.241 (2009).

97 Zheng, J. I. E., Benbrook, D. M., Yu, H. & Ding, W.-Q. Clioquinol Suppresses Cyclin D1 Gene Expression through Transcriptional and Post-transcriptional Mechanisms. *Anticancer Res.* **31**, 2739-2747 (2011).

98 Wang, Y. *et al.* miR-302a/b/c/d cooperatively inhibit BCRP expression to increase drug sensitivity in breast cancer cells. *Gynecol. Oncol.* **141**, 592-601, doi:10.1016/j.ygyno.2015.11.034 (2016).

99 Zhao, L. *et al.* MiR-302a/b/c/d cooperatively sensitizes breast cancer cells to adriamycin via suppressing P-glycoprotein(P-gp) by targeting MAP/ERK kinase kinase 1 (MEKK1). *J. Exp. Clin. Cancer Res.* **35**, 25, doi:10.1186/s13046-016-0300-8 (2016).

100 Sun, X., Cui, M., Tong, L., Zhang, A. & Wang, K. Upregulation of microRNA-3129 suppresses epithelial ovarian cancer through CD44. *Cancer Gene Ther.*, doi:10.1038/s41417-018-0026-1 (2018).

101 Shin, O. S., Kumar, M., Yanagihara, R. & Song, J.-W. Hantaviruses induce cell type- and viral species-specific host microRNA expression signatures. *Virology* **446**, 217-224, doi:<https://doi.org/10.1016/j.virol.2013.07.036> (2013).

102 Xu, C. Z. *et al.* Gene and microRNA expression reveals sensitivity to paclitaxel in laryngeal cancer cell line. *Int J Clin Exp Pathol* **6**, 1351-1361 (2013).

103 Kim, G. *et al.* Hsa-miR-1246 and hsa-miR-1290 are associated with stemness and invasiveness of non-small cell lung cancer. *Lung Cancer* **91**, 15-22, doi:<https://doi.org/10.1016/j.lungcan.2015.11.013> (2016).

104 Cheng, D., He, H. & Liang, B. A three-microRNA signature predicts clinical outcome in breast cancer patients. *Eur. Rev. Med. Pharmacol. Sci.* **22**, 6386-6395, doi:10.26355/eurrev_201810_16051 (2018).

105 Fite, K. & Gomez-Cambronero, J. Down-regulation of MicroRNAs (MiRs) 203, 887, 3619 and 182 Prevents Vimentin-triggered, Phospholipase D (PLD)-mediated Cancer Cell Invasion. *J. Biol. Chem.* **291**, 719-730, doi:10.1074/jbc.M115.686006 (2016).

106 OKUMURA, T. *et al.* MicroRNA Profiles to Predict Postoperative Prognosis in Patients with Small Cell Carcinoma of the Esophagus. *Anticancer Res.* **35**, 719-727 (2015).

107 Jayawardana, K. *et al.* Identification, Review, and Systematic Cross-Validation of microRNA Prognostic Signatures in Metastatic Melanoma. *J. Invest. Dermatol.* **136**, 245-254, doi:<https://doi.org/10.1038/JID.2015.355> (2016).

108 Wang, B., Li, J., Sun, M., Sun, L. & Zhang, X. MiRNA expression in breast cancer varies with lymph node metastasis and other clinicopathologic features. *IUBMB Life* **66**, 371-377, doi:10.1002/iub.1273 (2014).

109 Lee, W. H. *et al.* Deduction of novel genes potentially involved in hypoxic AC16 human cardiomyocytes using next-generation sequencing and bioinformatics approaches. *Int. J. Mol. Med.* **42**, 2489-2502, doi:10.3892/ijmm.2018.3851 (2018).

110 Benetatos, L. *et al.* The microRNAs within the DLK1-DIO3 genomic region: involvement in disease pathogenesis. *Cell. Mol. Life Sci.* **70**, 795-814, doi:10.1007/s00018-012-1080-8 (2013).

111 Zhang, Y. F. *et al.* miR-410-3p suppresses breast cancer progression by targeting Snail. *Oncol. Rep.* **36**, 480-486, doi:10.3892/or.2016.4828 (2016).

112 Wu, H., Li, J., Guo, E., Luo, S. & Wang, G. MiR-410 Acts as a Tumor Suppressor in Estrogen Receptor-Positive Breast Cancer Cells by Directly Targeting ERLIN2 via the ERS Pathway. *Cell. Physiol. Biochem.* **48**, 461-474 (2018).

113 Liu, D. *et al.* MiR-410 Down-Regulates the Expression of Interleukin-10 by Targeting STAT3 in the Pathogenesis of Systemic Lupus Erythematosus. *Cell. Physiol. Biochem.* **39**, 303-315, doi:10.1159/000445625 (2016).

114 Kanlikilicer, P. *et al.* Ubiquitous release of exosomal tumor suppressor miR-6126 from ovarian cancer cells. *Cancer Res.*, canres.0714.2016, doi:10.1158/0008-5472.CAN-16-0714 (2016).

115 Adhami, M., MotieGhader, H., Haghdoost, A. A., Afshar, R. M. & Sadeghi, B. Gene co-expression network approach for predicting prognostic microRNA biomarkers in different subtypes of breast cancer. *Genomics*, doi:<https://doi.org/10.1016/j.ygeno.2019.01.010> (2019).

116 Ding, C. F. *et al.* Circulating microRNAs in patients with polycystic ovary syndrome. *Human fertility (Cambridge, England)* **18**, 22-29, doi:10.3109/14647273.2014.956811 (2015).

117 Singh, R. P. *et al.* The role of miRNA in inflammation and autoimmunity. *Autoimmun. Rev.* **12**, 1160-1165, doi:<https://doi.org/10.1016/j.autrev.2013.07.003> (2013).

118 Boo, L. *et al.* MiRNA Transcriptome Profiling of Spheroid-Enriched Cells with Cancer Stem Cell Properties in Human Breast MCF-7 Cell Line. *International journal of biological sciences* **12**, 427-445, doi:10.7150/ijbs.12777 (2016).

119 Shimomura, A. *et al.* Novel combination of serum microRNA for detecting breast cancer in the early stage. *Cancer Sci* **107**, 326-334, doi:10.1111/cas.12880 (2016).

120 Zhong, X. *et al.* MiR-4653-3p and its target gene FRS2 are prognostic biomarkers for hormone receptor positive breast cancer patients receiving tamoxifen as adjuvant endocrine therapy. *Oncotarget* **7**, 61166-61182, doi:10.18632/oncotarget.11278 (2016).

121 Fujimori, T. *et al.* Antitumor effect of metformin on cholangiocarcinoma: In vitro and in vivo studies. *Oncol. Rep.* **34**, 2987-2996, doi:10.3892/or.2015.4284 (2015).

122 Kim, B. G. *et al.* Transcriptome-wide analysis of compression-induced microRNA expression alteration in breast cancer for mining therapeutic targets. *Oncotarget* **7**, 27468-27478, doi:10.18632/oncotarget.8322 (2016).

123 Li, L. *et al.* Profiling of microRNAs in AML cells following overexpression or silencing of the VEGF gene. *Oncology letters* **13**, 105-110, doi:10.3892/ol.2016.5412 (2017).

124 Yu, S. *et al.* Circulating microRNA124-3p, microRNA9-3p and microRNA196b-5p may be potential signatures for differential diagnosis of thyroid nodules. *Oncotarget* **7**, 84165-84177, doi:10.18632/oncotarget.12389 (2016).

125 Zhang, K. *et al.* Identification of microRNA biomarkers in the blood of breast cancer patients based on microRNA profiling. *Gene* **619**, 10-20, doi:<https://doi.org/10.1016/j.gene.2017.03.038> (2017).

126 Arora, S. *et al.* MicroRNA-4723 Inhibits Prostate Cancer Growth through Inactivation of the Abelson Family of Nonreceptor Protein Tyrosine Kinases. *PLOS ONE* **8**, e78023, doi:10.1371/journal.pone.0078023 (2013).

127 Berillo, O., Régnier, M. & Ivashchenko, A. Binding of intronic miRNAs to the mRNAs of host genes encoding intronic miRNAs and proteins that participate in tumourigenesis. *Comput. Biol. Med.* **43**, 1374-1381, doi:<https://doi.org/10.1016/j.compbiomed.2013.07.011> (2013).

128 Bong, I. P. N., Ng, C. C., Baharuddin, P. & Zakaria, Z. MicroRNA expression patterns and target prediction in multiple myeloma development and malignancy. *Genes & genomics* **39**, 533-540, doi:10.1007/s13258-017-0518-7 (2017).

129 Mody, H. R., Hung, S. W., AlSaggar, M., Griffin, J. & Govindarajan, R. Inhibition of S-Adenosylmethionine-Dependent Methyltransferase Attenuates TGFβ1-Induced EMT and Metastasis in Pancreatic Cancer: Putative Roles of miR-663a and miR-4787-5p. *Mol. Cancer Res.* **14**, 1124-1135, doi:10.1158/1541-7786.Mcr-16-0083 (2016).

130 Pu, Q. *et al.* Tissue-specific and plasma microRNA profiles could be promising biomarkers of histological classification and TNM stage in non-small cell lung cancer. *Thoracic cancer* **7**, 348-354, doi:10.1111/1759-7714.12317 (2016).

131 Yang, F., Zhang, W., Shen, Y. & Guan, X. Identification of dysregulated microRNAs in triple-negative breast cancer (review). *Int. J. Oncol.* **46**, 927-932, doi:10.3892/ijo.2015.2821 (2015).

132 Fricke, A. *et al.* Identification of a blood-borne miRNA signature of synovial sarcoma. *Molecular Cancer* **14**, 151, doi:10.1186/s12943-015-0424-z (2015).

133 Daniel, R. *et al.* A Panel of MicroRNAs as Diagnostic Biomarkers for the Identification of Prostate Cancer. *International Journal of Molecular Sciences* **18**, doi:10.3390/ijms18061281 (2017).

134 Jia, L., Liu, W., Cao, B., Li, H. & Yin, C. MiR-507 inhibits the migration and invasion of human breastcancer cells through Flt-1 suppression. *Oncotarget* **7**, 36743-36754, doi:10.18632/oncotarget.9163 (2016).

135 Wen, W. *et al.* Cellular microRNA-miR-548g-3p modulates the replication of dengue virus. *J. Infect.* **70**, 631-640, doi:<https://doi.org/10.1016/j.jinf.2014.12.001> (2015).

136 Tandon, M., Gallo, A., Jang, S. I., Illei, G. G. & Alevizos, I. Deep sequencing of short RNAs reveals novel microRNAs in minor salivary glands of patients with Sjögren’s syndrome. *Oral Dis.* **18**, 127-131, doi:10.1111/j.1601-0825.2011.01849.x (2012).

137 Yang, X. & Wu, X. miRNA expression profile of vulvar squamous cell carcinoma and identification of the oncogenic role of miR-590-5p. *Oncol. Rep.* **35**, 398-408, doi:10.3892/or.2015.4344 (2016).

138 Zhang, Y., Talmon, G. & Wang, J. MicroRNA-587 antagonizes 5-FU-induced apoptosis and confers drug resistance by regulating PPP2R1B expression in colorectal cancer. *Cell Death &Amp; Disease* **6**, e1845, doi:10.1038/cddis.2015.200 (2015).

139 Kojima, M. *et al.* MicroRNA Markers for the Diagnosis of Pancreatic and Biliary-Tract Cancers. *PLOS ONE* **10**, e0118220, doi:10.1371/journal.pone.0118220 (2015).

140 Shang, Z. & Li, H. Altered expression of four miRNA (miR-1238-3p, miR-202-3p, miR-630 and miR-766-3p) and their potential targets in peripheral blood from vitiligo patients. *The Journal of Dermatology* **44**, 1138-1144, doi:doi:10.1111/1346-8138.13886 (2017).

141 Chen, P.-H. *et al.* The miR-204-3p-targeted IGFBP2 pathway is involved in xanthohumol-induced glioma cell apoptotic death. *Neuropharmacology* **110**, 362-375, doi:10.1016/j.neuropharm.2016.07.038 (2016).

142 Navarro-Quiroz, E. *et al.* Profiling analysis of circulating microRNA in peripheral blood of patients with class IV lupus nephritis. *PLOS ONE* **12**, e0187973, doi:10.1371/journal.pone.0187973 (2017).

143 Cao, S. *et al.* Novel circular RNA expression profiles reflect progression of patients with hypopharyngeal squamous cell carcinoma. *Oncotarget* **8**, 45367-45379, doi:10.18632/oncotarget.17488 (2017).

144 Benway, C. J. & Iacomini, J. Defining a microRNA-mRNA interaction map for calcineurin inhibitor induced nephrotoxicity. *Am. J. Transplantation* **18**, 796-809, doi:doi:10.1111/ajt.14503 (2018).

145 Giulietti, M., Occhipinti, G., Principato, G. & Piva, F. Identification of candidate miRNA biomarkers for pancreatic ductal adenocarcinoma by weighted gene co-expression network analysis. *Cellular oncology (Dordrecht)* **40**, 181-192, doi:10.1007/s13402-017-0315-y (2017).

146 Xiong, H. *et al.* LncRNA HULC triggers autophagy via stabilizing Sirt1 and attenuates the chemosensitivity of HCC cells. *Oncogene* **36**, 3528-3540, doi:10.1038/onc.2016.521 (2017).

147 Chang, J. *et al.* A Rare Missense Variant in TCF7L2 Associates with Colorectal Cancer Risk by Interacting with a GWAS-Identified Regulatory Variant in the MYC Enhancer. *Cancer Res.* **78**, 5164 (2018).

148 Latchana, N. *et al.* Plasma MicroRNA Levels Following Resection of Metastatic Melanoma. *Bioinformatics and biology insights* **11**, 1177932217694837, doi:10.1177/1177932217694837 (2017).

149 Latchana, N. *et al.* Global microRNA profiling for diagnostic appraisal of melanocytic Spitz tumors. *J. Surg. Res.* **205**, 350-358, doi:10.1016/j.jss.2016.06.085 (2016).

150 Falkenberg, N. *et al.* MiR-221/-222 differentiate prognostic groups in advanced breast cancers and influence cell invasion. *Br. J. Cancer*, doi:10.1038/bjc.2013.625 (2013).

151 Chen, J. *et al.* Bioinformatics identification of dysregulated microRNAs in triple negative breast cancer based on microRNA expression profiling. *Oncology letters* **15**, 3017-3023, doi:10.3892/ol.2017.7707 (2018).

152 Zhao, J.-J. *et al.* MicroRNA-221/222 negatively regulates ERalpha and associates with tamoxifen resistance in breast cancer. *J. Biol. Chem.* **283**, 31079-31086, doi:10.1074/jbc.M806041200 (2008).

153 Zhang, C. *et al.* PUMA is a novel target of miR-221/222 in human epithelial cancers. *Int. J. Oncol.* **37**, 1621-1626 (2010).

154 Stinson, S. *et al.* TRPS1 targeting by miR-221/222 promotes the epithelial-to-mesenchymal transition in breast cancer. *Sci Signal* **4**, ra41, doi:10.1126/scisignal.2001538 (2011).

155 Ye, Z., Hao, R., Cai, Y., Wang, X. & Huang, G. Knockdown of miR-221 promotes the cisplatin-inducing apoptosis by targeting the BIM-Bax/Bak axis in breast cancer. *Tumor Biol.* **37**, 4509-4515, doi:10.1007/s13277-015-4267-4 (2015).

156 Liang, Y. K. *et al.* MiR-221/222 promote epithelial-mesenchymal transition by targeting Notch3 in breast cancer cell lines. *NPJ breast cancer* **4**, 20, doi:10.1038/s41523-018-0073-7 (2018).

157 Santolla, M. F. *et al.* miR-221 stimulates breast cancer cells and cancer-associated fibroblasts (CAFs) through selective interference with the A20/c-Rel/CTGF signaling. *J. Exp. Clin. Cancer Res.* **37**, 94, doi:10.1186/s13046-018-0767-6 (2018).

158 Deng, L. *et al.* Downregulation of miR-221-3p and upregulation of its target gene PARP1 are prognostic biomarkers for triple negative breast cancer patients and associated with poor prognosis. *Oncotarget* **8**, 108712-108725, doi:10.18632/oncotarget.21561 (2017).

159 Howe, E., Cochrane, D. & Richer, J. The miR-200 and miR-221/222 microRNA Families: Opposing Effects on Epithelial Identity. *J. Mammary Gland Biol. Neoplasia* **17**, 65-77, doi:10.1007/s10911-012-9244-6 (2012).

160 Dai, X., Chen, A. & Bai, Z. Integrative investigation on breast cancer in ER, PR and HER2-defined subgroups using mRNA and miRNA expression profiling. *Sci. Rep.* **4**, doi:10.1038/srep06566 (2014).

161 Turashvili, G. *et al.* Novel prognostic and predictive microRNA targets for triple-negative breast cancer. *FASEB J.*, fj201800120R, doi:10.1096/fj.201800120R (2018).

162 Vilquin, P. *et al.* MicroRNA-125b upregulation confers aromatase inhibitor resistance and is a novel marker of poor prognosis in breast cancer. *Breast Cancer Res* **17**, 13, doi:10.1186/s13058-015-0515-1 (2015).

163 Cheng, Y. *et al.* MiRNA-224-5p inhibits autophagy in breast cancer cells via targeting Smad4. *Biochem. Biophys. Res. Commun.*, doi:10.1016/j.bbrc.2018.10.150 (2018).

164 Chai, P. *et al.* GSE1 negative regulation by miR-489-5p promotes breast cancer cell proliferation and invasion. *Biochem. Biophys. Res. Commun.* **471**, 123-128, doi:10.1016/j.bbrc.2016.01.168 (2016).

165 Tavazoie, S. F. *et al.* Endogenous human microRNAs that suppress breast cancer metastasis. *Nature* **451**, 147-152 (2008).

166 Lee, Y.-M. *et al.* miRNA-34b as a tumor suppressor in estrogen-dependent growth of breast cancer cells. *Breast Cancer Research* **13**, R116 (2011).

167 Pinho, F. G. *et al.* Downregulation of microRNA-515-5p by the estrogen receptor modulates sphingosine kinase-1 and breast cancer cell proliferation. *Cancer Res.* **73**, 5936-5948, doi:10.1158/0008-5472.can-13-0158 (2013).

168 Joshi, T. *et al.* Integrative analysis of miRNA and gene expression reveals regulatory networks in tamoxifen-resistant breast cancer. *Oncotarget* **7**, 57239-57253, doi:10.18632/oncotarget.11136 (2016).

169 Pardo, O. E. *et al.* miR-515-5p controls cancer cell migration through MARK4 regulation. *EMBO Rep* **17**, 570-584, doi:10.15252/embr.201540970 (2016).

170 Papaioannou, M. D., Koufaris, C. & Gooderham, N. J. The cooked meat-derived mammary carcinogen 2-amino-1-methyl-6-phenylimidazo[4,5-b]pyridine (PhIP) elicits estrogenic-like microRNA responses in breast cancer cells. *Toxicol. Lett.* **229**, 9-16, doi:10.1016/j.toxlet.2014.05.021 (2014).

171 Liu, J., Wang, D., Long, Z., Liu, J. & Li, W. CircRNA8924 Promotes Cervical Cancer Cell Proliferation, Migration and Invasion by Competitively Binding to MiR-518d-5p /519-5p Family and Modulating the Expression of CBX8. *Cell. Physiol. Biochem.* **48**, 173-184, doi:10.1159/000491716 (2018).

172 Keklikoglou, I. *et al.* MicroRNA-520/373 family functions as a tumor suppressor in estrogen receptor negative breast cancer by targeting NF-[kappa]B and TGF-[beta] signaling pathways. *Oncogene* **31**, 4150-4163, doi:10.1038/onc.2011.571 (2012).

173 Yang, X. *et al.* miR-526a regulates apoptotic cell growth in human carcinoma cells. *Mol. Cell. Biochem.* **407**, 69-76, doi:10.1007/s11010-015-2455-6 (2015).

174 Tang, S. *et al.* Stratification of Digestive Cancers with Different Pathological Features and Survival Outcomes by MicroRNA Expression. *Scientific reports* **6**, 24466, doi:10.1038/srep24466 (2016).

175 Gottardo, F. *et al.* Micro-RNA profiling in kidney and bladder cancers. *Urologic Oncology: Seminars and Original Investigations* **25**, 387-392, doi:10.1016/j.urolonc.2007.01.019 (2007).

176 Zhao, Y. *et al.* let-7 MicroRNAs Induce Tamoxifen Sensitivity by Downregulation of Estrogen Receptor α Signaling in Breast Cancer. *Molecular Medicine* **17**, 1233-1241, doi:10.2119/molmed.2010.00225 (2011).

177 Morata-Tarifa, C. *et al.* Low adherent cancer cell subpopulations are enriched in tumorigenic and metastatic epithelial-to-mesenchymal transition-induced cancer stem-like cells. *Scientific reports* **6**, 18772, doi:10.1038/srep18772 (2016).

178 Petrelli, A. *et al.* By promoting cell differentiation, miR-100 sensitizes basal-like breast cancer stem cells to hormonal therapy. *Oncotarget* **6**, 2315-2330, doi:10.18632/oncotarget.2962 (2015).

179 Li, C. Y. *et al.* Clinical Value of miR-101-3p and Biological Analysis of its Prospective Targets in Breast Cancer: A Study Based on The Cancer Genome Atlas (TCGA) and Bioinformatics. *Med Sci Monit* **23**, 1857-1871 (2017).

180 Liu, P. *et al.* mir-101-3p is a key regulator of tumor metabolism in triple negative breast cancer targeting AMPK. *Oncotarget* **7**, 35188-35198, doi:10.18632/oncotarget.9072 (2016).

181 Sachdeva, M. *et al.* MicroRNA-101-mediated Akt activation and estrogen-independent growth. *Oncogene* **30**, 822-831, doi:10.1038/onc.2010.463 (2011).

182 Yang, X. *et al.* Diagnostic value of strand-specific miRNA-101-3p and miRNA-101-5p for hepatocellular carcinoma and a bioinformatic analysis of their possible mechanism of action. *FEBS Open Bio* **8**, 64-84, doi:10.1002/2211-5463.12349 (2018).

183 Slotta-Huspenina, J. *et al.* MicroRNA expression profiling for the prediction of resistance to neoadjuvant radiochemotherapy in squamous cell carcinoma of the esophagus. *Journal of Translational Medicine* **16**, 109, doi:10.1186/s12967-018-1492-9 (2018).

184 Li, Y. *et al.* Knockdown of microRNA-1323 restores sensitivity to radiation by suppression of PRKDC activity in radiation-resistant lung cancer cells. *Oncol. Rep.* **33**, 2821-2828, doi:10.3892/or.2015.3884 (2015).

185 Li, G. *et al.* Genome-Wide Analyses of Radioresistance-Associated miRNA Expression Profile in Nasopharyngeal Carcinoma Using Next Generation Deep Sequencing. *PLOS ONE* **8**, e84486, doi:10.1371/journal.pone.0084486 (2013).

186 Law, P. T. Y. *et al.* Deep sequencing of small RNA transcriptome reveals novel non-coding RNAs in hepatocellular carcinoma. *J. Hepatol.* **58**, 1165-1173, doi:10.1016/j.jhep.2013.01.032 (2013).

187 Chang, C. *et al.* MicroRNA-134-3p is a novel potential inhibitor of human ovarian cancer stem cells by targeting RAB27A. *Gene* **605**, 99-107, doi:10.1016/j.gene.2016.12.030 (2017).

188 Chen, Y. *et al.* miRNA-135a promotes breast cancer cell migration and invasion by targeting HOXA10. *BMC Cancer* **12**, 111, doi:10.1186/1471-2407-12-111 (2012).

189 Kim, K., Madak-Erdogan, Z., Ventrella, R. & Katzenellenbogen, B. S. A MicroRNA196a2* and TP63 circuit regulated by estrogen receptor-alpha and ERK2 that controls breast cancer proliferation and invasiveness properties. *Horm Cancer* **4**, 78-91, doi:10.1007/s12672-012-0129-3 (2013).

190 Tribollet, V. *et al.* miR-135a Inhibits the Invasion of Cancer Cells via Suppression of ERRα. *PLOS ONE* **11**, e0156445, doi:10.1371/journal.pone.0156445 (2016).

191 Ahmad, A., Zhang, W., Wu, M., Tan, S. & Zhu, T. Tumor-suppressive miRNA-135a inhibits breast cancer cell proliferation by targeting ELK1 and ELK3 oncogenes. *Genes & genomics* **40**, 243-251, doi:10.1007/s13258-017-0624-6 (2018).

192 Waters, P. S. *et al.* Impact of Tumour Epithelial Subtype on Circulating microRNAs in Breast Cancer Patients. *PLOS ONE* **9**, e90605, doi:10.1371/journal.pone.0090605 (2014).

193 Zhang, J. *et al.* MicroRNA-138 modulates metastasis and EMT in breast cancer cells by targeting vimentin. *Biomed. Pharmacother.* **77**, 135-141, doi:10.1016/j.biopha.2015.12.018 (2016).

194 Sachdeva, M. *et al.* p53 represses c-Myc through induction of the tumor suppressor miR-145. *Proceedings of the National Academy of Sciences* **106**, 3207-3212, doi:10.1073/pnas.0808042106 (2009).

195 Xu, Q. *et al.* MiR-145 directly targets p70S6K1 in cancer cells to inhibit tumor growth and angiogenesis. *Nucleic Acids Res.* **40**, 761-774, doi:10.1093/nar/gkr730 (2012).

196 Wang, S. *et al.* CircZNF609 promotes breast cancer cell growth, migration, and invasion by elevating p70S6K1 via sponging miR-145-5p. *Cancer management and research* **10**, 3881-3890, doi:10.2147/cmar.S174778 (2018).

197 Jiang, R. *et al.* Mixomics analysis of breast cancer: Long non-coding RNA linc01561 acts as ceRNA involved in the progression of breast cancer. *The International Journal of Biochemistry & Cell Biology* **102**, 1-9, doi:10.1016/j.biocel.2018.06.003 (2018).

198 Calvano Filho, C. M. *et al.* Triple-negative and luminal a breast tumors: differential expression of miR-18a-5p, miR-17-5p, and miR-20a-5p. *Tumour Biol.* **35**, doi:10.1007/s13277-014-2025-7 (2014).

199 Hossain, A., Kuo, M. T. & Saunders, G. F. miR-17-5p Regulates Breast Cancer Cell Proliferation by Inhibiting Translation of AIB1 mRNA. *Mol. Cell. Biol.* **26**, 8191-8201 (2006).

200 Li, H., Bian, C., Liao, L., Li, J. & Zhao, R. C. miR-17-5p promotes human breast cancer cell migration and invasion through suppression of HBP1. *Breast Cancer Res. Treat.* **126**, 565-575, doi:10.1007/s10549-010-0954-4 (2011).

201 Li, X. *et al.* Urokinase-type plasminogen activator receptor inhibits apoptosis in triple-negative breast cancer through miR-17/20a suppression of death receptors 4 and 5. *Oncotarget* **8**, 88645-88657, doi:10.18632/oncotarget.20435 (2017).

202 Li, J. *et al.* miR-17-5p suppresses cell proliferation and invasion by targeting ETV1 in triple-negative breast cancer. *BMC Cancer* **17**, 745, doi:10.1186/s12885-017-3674-x (2017).

203 O'Day, E. & Lal, A. MicroRNAs and their target gene networks in breast cancer. *Breast Cancer Res* **12**, 201, doi:10.1186/bcr2484 (2010).

204 Fan, M., Sethuraman, A., Brown, M., Sun, W. & Pfeffer, L. M. Systematic analysis of metastasis-associated genes identifies miR-17-5p as a metastatic suppressor of basal-like breast cancer. *Breast Cancer Res. Treat.* **146**, doi:10.1007/s10549-014-3040-5 (2014).

205 Bobbili, M. R., Mader, R. M., Grillari, J. & Dellago, H. OncomiR-17-5p: alarm signal in cancer? *Oncotarget* **8**, 71206-71222, doi:10.18632/oncotarget.19331 (2017).

206 Murria Estal, R. *et al.* MicroRNA signatures in hereditary breast cancer. *Breast Cancer Res. Treat.* **142**, 19-30, doi:10.1007/s10549-013-2723-7 (2013).

207 Dou, C. *et al.* miR-187-3p inhibits the metastasis and epithelial–mesenchymal transition of hepatocellular carcinoma by targeting S100A4. *Cancer Lett.* **381**, 380-390, doi:10.1016/j.canlet.2016.08.011 (2016).

208 Xie, F. *et al.* MicroRNA-193a inhibits breast cancer proliferation and metastasis by downregulating WT1. *PLOS ONE* **12**, e0185565, doi:10.1371/journal.pone.0185565 (2017).

209 Chen, K. *et al.* Methylation-associated silencing of miR-193a-3p promotes ovarian cancer aggressiveness by targeting GRB7 and MAPK/ERK pathways. *Theranostics* **8**, 423-436, doi:10.7150/thno.22377 (2018).

210 Tsai, K.-W. *et al.* Arm Selection Preference of MicroRNA-193a Varies in Breast Cancer. *Scientific reports* **6**, 28176, doi:10.1038/srep28176 (2016).

211 Wu, Q. *et al.* Analysis of the miRNA-mRNA-lncRNA networks in ER+ and ER- breast cancer cell lines. *J Cell Mol Med* **19**, 2874-2887, doi:10.1111/jcmm.12681 (2015).

212 Lee, S., Lee, H., Bae, H., Choi, E. H. & Kim, S. J. Epigenetic silencing of miR-19a-3p by cold atmospheric plasma contributes to proliferation inhibition of the MCF-7 breast cancer cell. *Scientific reports* **6**, 30005, doi:10.1038/srep30005 (2016).

213 Maleki, E., Ghaedi, K., Shahanipoor, K. & Karimi Kurdistani, Z. Down-regulation of microRNA-19b in hormone receptor-positive/HER2-negative breast cancer. *APMIS* **126**, 303-308, doi:10.1111/apm.12820 (2018).

214 Jin, J. *et al.* miR-19b-3p inhibits breast cancer cell proliferation and reverses saracatinib-resistance by regulating PI3K/Akt pathway. *Arch. Biochem. Biophys.* **645**, 54-60, doi:10.1016/j.abb.2018.03.015 (2018).

215 Zhao, W., Geng, D., Li, S., Chen, Z. & Sun, M. LncRNA HOTAIR influences cell growth, migration, invasion, and apoptosis via the miR-20a-5p/HMGA2 axis in breast cancer. *Cancer medicine* **7**, 842-855, doi:10.1002/cam4.1353 (2018).

216 Bai, X., Han, G., Liu, Y., Jiang, H. & He, Q. MiRNA-20a-5p promotes the growth of triple-negative breast cancer cells through targeting RUNX3. *Biomed. Pharmacother.* **103**, 1482-1489, doi:10.1016/j.biopha.2018.04.165 (2018).

217 Jansen, M. P. *et al.* High miR-26a and low CDC2 levels associate with decreased EZH2 expression and with favorable outcome on tamoxifen in metastatic breast cancer. *Breast Cancer Res. Treat.* **133**, 937-947, doi:10.1007/s10549-011-1877-4 (2012).

218 Tan, S. *et al.* Identification of miR-26 as a key mediator of estrogen stimulated cell proliferation by targeting CHD1, GREB1 and KPNA2. *Breast Cancer Res* **16**, R40, doi:10.1186/bcr3644 (2014).

219 Gao, J. *et al.* MiR-26a inhibits proliferation and migration of breast cancer through repression of MCL-1. *PLoS One* **8**, e65138, doi:10.1371/journal.pone.0065138 (2013).

220 Maillot, G. *et al.* Widespread Estrogen-Dependent Repression of microRNAs Involved in Breast Tumor Cell Growth. *Cancer Res.* **69**, 8332-8340, doi:10.1158/0008-5472.can-09-2206 (2009).

221 Ichikawa, T. *et al.* Trastuzumab produces therapeutic actions by upregulating miR-26a and miR-30b in breast cancer cells. *PLoS One* **7**, e31422, doi:10.1371/journal.pone.0031422 (2012).

222 Cabello, P., Pineda, B., Tormo, E., Lluch, A. & Eroles, P. The Antitumor Effect of Metformin Is Mediated by miR-26a in Breast Cancer. *Int J Mol Sci* **17**, doi:10.3390/ijms17081298 (2016).

223 Castellano, L. *et al.* Sustained expression of miR-26a promotes chromosomal instability and tumorigenesis through regulation of CHFR. *Nucleic Acids Res.* **45**, 4401-4412, doi:10.1093/nar/gkx022 (2017).

224 Wang, Y., Zhang, X., Li, H., Yu, J. & Ren, X. The role of miRNA-29 family in cancer. *Eur. J. Cell Biol.* **92**, 123-128, doi:10.1016/j.ejcb.2012.11.004 (2013).

225 Shinden, Y. *et al.* miR-29b is an indicator of prognosis in breast cancer patients. *Molecular and clinical oncology* **3**, 919-923, doi:10.3892/mco.2015.565 (2015).

226 Melo, S. A. & Kalluri, R. miR-29b moulds the tumour microenvironment to repress metastasis. *Nat Cell Biol* **15**, 139-140 (2013).

227 Zhao, H. *et al.* miR-29b defines the pro-/anti-proliferative effects of S100A7 in breast cancer. *Mol Cancer* **14**, 11, doi:10.1186/s12943-014-0275-z (2015).

228 Hermansen, S. K. *et al.* A 4-miRNA signature to predict survival in glioblastomas. *PLOS ONE* **12**, e0188090, doi:10.1371/journal.pone.0188090 (2017).

229 Perez-Carbonell, L. *et al.* MiR-320e is a novel prognostic biomarker in colorectal cancer. *Br. J. Cancer* **113**, 83, doi:10.1038/bjc.2015.168 (2015).

230 Svoboda, M. *et al.* MiR-34b is associated with clinical outcome in triple-negative breast cancer patients. *Diagnostic Pathology* **7**, 31 (2012).

231 Achari, C., Winslow, S., Ceder, Y. & Larsson, C. Expression of miR-34c induces G2/M cell cycle arrest in breast cancer cells. *BMC Cancer* **14**, 538, doi:10.1186/1471-2407-14-538 (2014).

232 Lu, J. *et al.* Radiation Enhances the Epithelial- Mesenchymal Transition of A549 Cells via miR3591-5p/USP33/PPM1A. *Cell. Physiol. Biochem.* **50**, 721-733, doi:10.1159/000494238 (2018).

233 Soronen, J. *et al.* Novel hepatic microRNAs upregulated in human nonalcoholic fatty liver disease. *Physiological Reports* **4** (2016).

234 Shivapurkar, N., Vietsch, E. E., Carney, E., Isaacs, C. & Wellstein, A. Circulating microRNAs in patients with hormone receptor-positive, metastatic breast cancer treated with dovitinib. *Clinical and Translational Medicine* **6**, 37, doi:10.1186/s40169-017-0169-y (2017).

235 Wang, J., Wang, S., Zhou, J. & Qian, Q. miR-424-5p regulates cell proliferation, migration and invasion by targeting doublecortin-like kinase 1 in basal-like breast cancer. *Biomed. Pharmacother.* **102**, 147-152, doi:10.1016/j.biopha.2018.03.018 (2018).

236 Yu, F. Y. *et al.* MiR-4500 is epigenetically downregulated in colorectal cancer and functions as a novel tumor suppressor by regulating HMGA2. *Cancer Biol Ther* **17**, 1149-1157, doi:10.1080/15384047.2016.1235661 (2016).

237 MASUDA, T. *et al.* Circulating Pre-microRNA-488 in Peripheral Blood Is a Potential Biomarker for Predicting Recurrence in Breast Cancer. *Anticancer Res.* **38**, 4515-4523, doi:10.21873/anticanres.12755 (2018).

238 Han, L., Liu, B., Jiang, L., Liu, J. & Han, S. MicroRNA-497 downregulation contributes to cell proliferation, migration, and invasion of estrogen receptor alpha negative breast cancer by targeting estrogen-related receptor alpha. *Tumor Biol.* **37**, 13205-13214, doi:10.1007/s13277-016-5200-1 (2016).

239 Ren, G. B., Wang, L., Zhang, F. H., Meng, X. R. & Mao, Z. P. Study on the relationship between miR-520g and the development of breast cancer. *Eur. Rev. Med. Pharmacol. Sci.* **20**, 657-663 (2016).

240 Matamala, N. *et al.* MicroRNA deregulation in triple negative breast cancer reveals a role of miR-498 in regulating BRCA1 expression. *Oncotarget* **7**, 20068-20079, doi:10.18632/oncotarget.7705 (2016).

241 Feliciano, A. *et al.* miR-125b acts as a tumor suppressor in breast tumorigenesis via its novel direct targets ENPEP, CK2-alpha, CCNJ, and MEGF9. *PLoS One* **8**, e76247, doi:10.1371/journal.pone.0076247 (2013).

242 Sun, S. J., Wang, N., Sun, Z. W., Chen, J. & Cui, H. W. MiR-5692a promotes the invasion and metastasis of hepatocellular carcinoma via MMP9. *Eur. Rev. Med. Pharmacol. Sci.* **22**, 4869-4878, doi:10.26355/eurrev_201808_15623 (2018).

243 McDermott, A. M. *et al.* Identification and Validation of Oncologic miRNA Biomarkers for Luminal A-like Breast Cancer. *PLoS ONE* **9**, e87032, doi:10.1371/journal.pone.0087032 (2014).

244 Mangolini, A. *et al.* Diagnostic and prognostic microRNAs in the serum of breast cancer patients measured by droplet digital PCR. *Biomarker Research* **3**, 12, doi:10.1186/s40364-015-0037-0 (2015).

245 Mullany, L. E., Herrick, J. S., Wolff, R. K., Stevens, J. R. & Slattery, M. L. Association of cigarette smoking and microRNA expression in rectal cancer: Insight into tumor phenotype. *Cancer Epidemiology* **45**, 98-107, doi:<https://doi.org/10.1016/j.canep.2016.10.011> (2016).

246 Munoz-Rodriguez, J. L. *et al.* Differentially expressed microRNAs in postpartum breast cancer in Hispanic women. *PLoS One* **10**, e0124340, doi:10.1371/journal.pone.0124340 (2015).

247 He, H., Tian, W., Chen, H. & Jiang, K. MiR-944 functions as a novel oncogene and regulates the chemoresistance in breast cancer. *Tumor Biol.* **37**, 1599-1607, doi:10.1007/s13277-015-3844-x (2016).

248 Flores-Pérez, A. *et al.* Suppression of cell migration is promoted by miR-944 through targeting of SIAH1 and PTP4A1 in breast cancer cells. *BMC Cancer* **16**, 379, doi:10.1186/s12885-016-2470-3 (2016).

249 Pan, T. *et al.* miR-944 inhibits metastasis of gastric cancer by preventing the epithelial–mesenchymal transition via MACC1/Met/AKT signaling. *FEBS Open Bio* **7**, 905-914, doi:10.1002/2211-5463.12215 (2017).

250 Bhat-Nakshatri, P. *et al.* Estradiol-regulated microRNAs control estradiol response in breast cancer cells. *Nucl. Acids Res.* **37**, 4850-4861, doi:10.1093/nar/gkp500 (2009).

251 Siragam, V. *et al.* MicroRNA miR-98 inhibits tumor angiogenesis and invasion by targeting activin receptor-like kinase-4 and matrix metalloproteinase-11. *Oncotarget* **3**, 1370-1385, doi:10.18632/oncotarget.717 (2012).

252 Wang, M.-J., Zhang, H., Li, J. & Zhao, H.-D. microRNA-98 inhibits the proliferation, invasion, migration and promotes apoptosis of breast cancer cells by binding to HMGA2. *Biosci. Rep.* **38**, BSR20180571, doi:10.1042/BSR20180571 (2018).

FIGURE LEGENDS

**Figure 1: Expression of the genes encoding the readers, writers, and erasers of reversible m6A RNA modification**. A and B) Data are from a previous RNA-seq experiment in MCF-7 and LCC9 cells (GEO GSE81620). Data are the average of three replicate experiments +/- SEM. with FPKM = fragments Per Kilobase of transcript per Million mapped reads. * P < 0.05 in a two-tailed student’s t test. C) Representative western blot of HNRNPA2B1 protein expression in WCE from MCF-7 and LCC9 cells. The blot was stripped and reprobed for GAPDH. The numerical values are HNRNPA2B1/GAPDH in these blots. The full-length blot of GAPDH is shown in Supplementary Figure 1C. D) Summary of relative HNRNPA2B1 protein expression in LCC9 and LY2 cells compared to MCF-7 parental cells. P < 0.05, One way ANOVA followed by Tukey’s test.

**Figure 2: HNRNPA2B1 overexpression in MCF-7 cells.** A. The ΔCT values for HNRNPA2B1 normalized to 18S of each of the six samples used for RNA se. MCF-7 cells were transfected with pCDNA3 control or pCDNA-3-HNRNPA2B1. Each point is the mean of triplicate determinations within one qPCR run of these samples. *p< 0.05, One way ANOVA followed by Tukey’s test. B. Western blot for HNRNPA2B1 in MCF-7 cells control-transfected (C) and transfected with HNRNPA2B1 for 48 h. The blot was stripped and reprobed for GAPDH. Values are the HNRNPA2B1/GAPDH in this blot. The full-length blot of GAPDH is shown in Supplementary Figure 1D. C) Summary of relative HNRNPA2B1 protein expression in MCF-7 cells transfected for 48 h vs. control, n = mean ± std of 7 biological replicates. P < 0.0004, two-tailed student’s t-test. D. The heat map represents the miRNAs having a fold-change of ± 4. Yellow is upregulated and purple is downregulated (scale at top). Genes were clustered based on similar expression profiles.

**Figure 3: Venn diagram depicting the number of different and common miRNAs identified as upregulated after transient HNRNPA2B1 overexpression in MCF-7 cells after 48 or 72 h.** MetaCore Enrichment by Pathway Maps analysis of DE miRNAs upregulated after 48 h (left, # 1-5) and 72 h (right, 1-6) (both versus control) and those identified in common at 48 and 72 h (below, #1-10).

**Figure 4: Venn diagram depicting the number of different and common miRNAs identified as downregulated after transient HNRNPA2B1 overexpression in MCF-7 cells after 48 or 72 h.** MetaCore Enrichment by Pathway Maps analysis of DE downregulated miRNAs after 48 h and 72 h (both versus control) identified common and unique pathways putatively regulated by the DE miRNAs.

**Figure 5: Enriched GO:BP (Gene Ontology: Biological Processes) for genes targeted by differentially expressed miRNAs at the indicated times.** mRNA targets identified in miRTarBase as validated targets for the differentially expressed miRNAs at each time point were compared to control or the indicated comparison for GO:BP analysis in categoryCompare

**Figure 6: Enriched GO:BP (Gene Ontology: Biological Processes) for genes targeted by differentially expressed miRNAs at the indicated times.** mRNA targets identified in miRTarBase as validated targets for the differentially expressed miRNAs at each time point were compared to control or the indicated comparison for GO:BP analysis in categoryCompare.

**Figure 7: Regulation of miR-29a-3p, miR-29b-3p, and miR-222-3p by HNRNPA2B1.** MCF-7 cells were either non-transfected (control), transfected with pcDNA3.1 parental vector, or an expression vector for HNRNPA2B1 for 48 h. qPCR for **A)** miR-29a-3p; **B)** miR-29b-3p, and **C)** miR-222-3p. Each miRNA was normalized by RNU48. Values are relative expression normalized to control-transfected cells from 11 biological replicate experiments with multiple control and transfected wells in each experiment. Data were analyzed by two way ANOVA followed by Tukey’s *post hoc* test, * p < 0.05; ** p < 0.01. .

**Figure 8: Regulation of miR-1266-5p, miR-1268a, and miR-671-3p by HNRNPA2B1.** MCF-7 cells were either non-transfected (control), transfected with pcDNA3.1 parental vector (pcDNA), or an expression vector for HNRNPA2B1 for 48 h. qPCR for **A)** miR-1266-5p; **B)** miR-1268a, and **C)** miR-671-3p. Each miRNA was normalized by RNU48. Values are relative expression normalized to control-transfected cells from 11 biological replicate experiments with multiple control and transfected wells in each experiment. Data were analyzed by two way ANOVA followed by Tukey’s *post hoc* test, * p < 0.01; ** p < 0.001.

**Figure 9: Effect of transient HNRNPA2B1 cells on MCF-7 cell viability. A)** Results are the Absorbance readings at 490 nm from an MTT assay in which 5,000 MCF-7 cells were plated in OPTI-MEM for 18 h prior to transfection with vector control (pcDNA cont) or HNRNPA2B1 for 24 or 48 h followed by an MTT assay. Each bar is the avg. ± SEM of 4 wells in one experiment. B) MCF-7 cells were transfected with the pcDNA control vector or HNRNPA2B1 for 24 and then treated with vehicle control (DMSO), 100 nM or 1 µM 4-OHT or 100 nM fulvestrant for 48 h followed by an MTT assay. The control was set to 1 for each transfection. Each bar is the avg. ± SEM of 4 wells in one experiment. * p < 0.05 vs. control in both A and B. In B,**p<0.05 vs. the same treatment between control vs. HNRNPA2B-transfected cells. Student’s 2-tailed t-test was used for analysis.
