# Supplementary material for: HNRNPA2/B1 is upregulated in endocrine-resistant LCC9 breast cancer cells and alters the miRNA transcriptome when overexpressed in MCF-7 cells
Source: Sci Rep. 2019 Jul 1;9:9430. doi: 10.1038/s41598-019-45636-8 (PMC6603045; doi:10.1038/s41598-019-45636-8)
Supplement: Supplementary file 2 — Supplementary Tables and Figures [file 41598_2019_45636_MOESM2_ESM.pdf]

Supplementary Tables (7) and Figures (8)

HNRNPA2/B1 is upregulated in endocrine-resistant LCC9 breast cancer cells and alters the miRNA transcriptome when overexpressed in MCF-7 cells

Carolyn M. Klinge1, Kellianne M. Piell, Christine Schaner Tooley, and Eric C. Rouchka

List of files:

Supplementary Tables 1-7

Supplementary Table 1: Summary of Sequence Analysis The raw data of our RNA-seq are available at Gene Expression Omnibus (GEO) database: accession number GSE122634.

Supplementary Table 2: Upregulated differentially expressed miRNAs in HNRNPA2B1-transfected MCF-7 cells at T48 vs. control

Supplementary Table 3: Downregulated miRNAs differentially expressed in HNRNPA2B1-transfected MCF-7 cells at T48 vs. control

Supplementary Table 4: Up-regulated miRNAs differentially expressed miRs in HNRNPA2B1-transfected MCF-7 cells at T72 vs. control.

Supplementary Table 5: Downregulated miRNAs differentially expressed miRs in HNRNPA2B1-transfected MCF-7 cells at T72 vs. control.

Supplementary Table 6: Up-regulated differentially expressed miRNAs in HNRNPA2B1-transfected MCF-7 cells at T72 vs. T48.

Supplementary Table 7: Downregulated differentially expressed miRNAs in HNRNPA2B1-transfected MCF-7 cells at T72 vs. T48.

Supplementary Figures 1-8

Supplementary Figure 1: Kaplan–Meier estimator plots of the probability of overall survival of breast cancer patients based on primary tumor expression of the indicated gene. Data were from 3,554 breast tumors and are not sorted for ER or HER2 status. Data are from http://kmplot.com

Supplementary Figure 2: Kaplan–Meier estimator plots of the probability of overall survival of breast cancer patients based on primary tumor expression of the indicated gene. Data from http://kmplot.com.

Supplementary Figure 3: HNRNPA2B1 protein in MCF-7 and LCC9 cells; Full length GAPDH blots for Fig 1; and qPCR of GAPHD in cells transfected with pcDNA3 control for 48 or 72 h. A) For this gel, 45 g of WCE were separated on a 10% SDS-PAGE gel and transferred to a PVDF membrane, probed with IBL NRNPA2B1 (catalog #18941), stripped and reprobed for β-actin.

Values are HNRNPA2B1/ β-actin for the lanes shown. While HNRNPA2B1 is a 37 kDa protein it is known to form aggregates (Li *et al* Hum Mol Genet 2016). The estimated MW of the HNRNPA2B1 aggregates was calculated using UnScanIt. B) Full blot of β-actin from A is shown. C) Full blot of GAPDH for Figure 1C. D) Full blot of GAPDH for Figure 2B. E) qPCR for *GAPH* using TaqMan primers was run on 7 samples (each in quadruplicate) from MCF-7 cells transfected with pcDNA3 for 48 or 72 h. Values are the CT for GAPDH in each sample with the avg. ± stdev. plotted. There is no significant difference when analyzed by a two-tailed t-test in GraphPad Prism.

Supplementary Figure 4: Effect of HNRNPA2B1 transfection in MCF-7 cells for 48 or 72 h on miRNA expression. Heatmap represents the raw expression values with miRNAs clustered based on similar expression profiles. The scale is at the top right. Only miRNA transcripts that showed a log2 fold-change greater than 1 (or -1 for repressed miRNAs) were included. miRNAs down-regulated are black and upregulated yellow to white.

Supplementary Figure 5: Venn diagram depicting the number of different and common miRNAs identified as HNRNPA2B1 regulated in MCF-7 cells after 48 or 72 h of transfection. MetaCore Enrichment by Pathway Maps analysis of DE miRNAs (both up and downregulated) after 48 h and 72 h (both versus control) identified common (blue) and unique (black) pathways putatively regulated by the DE miRNAs.

Supplementary Figure 6: Venn diagram depicting the number of different and common miRNAs identified as upregulated after transient HNRNPA2B1 overexpression in MCF-7 cells after 48 or 72 h. MetaCore Enrichment by GO Processes analysis of DE miRNAs upregulated after 48 h and 72 h (both versus control) identified common to 48 h (blue), common to 72 h (red) and unique (black) pathways putatively regulated by the DE miRNAs.

Supplementary Figure 7: Venn diagram depicting the number of different and common miRNAs identified as downregulated after transient HNRNPA2B1 overexpression in MCF-7 cells after 48 or 72 h. MetaCore Enrichment by GO processes of DE downregulated miRNAs after 48 h and 72 h (both versus control) identified as GO processes putatively regulated by the DE miRNAs

Supplementary Figure 8: Enriched KEGG for genes targeted by differentially expressed miRNAs at the indicated times

Supplementary Table 1: Summary of Sequence Analysis The raw data of our RNA-seq are available at Gene Expression Omnibus (GEO) database: accession number GSE122634.

**Sample**	**Raw Reads**	**After Trimming**	**Secondary Trimming**	**Aligned Reads**
CK-01-TF-pcDNA3-HNRNPA2B1-72h-1_S1	20,784,218	20,609,822	9,971,429	5,823,107 (28%)
CK-02-TF-pcDNA3-HNRNPA2B1-72h-2_S2	21,510,449	21,374,568	9,554,508	6,309,282 (29%)
CK-03-TF-pcDNA3-HNRNPA2B1-72h-3_S3	21,454,874	21,303,148	8,553,759	5,602,640 (26%)
CK-04-TF-pcDNA3-HNRNPA2B1-72h-4_S4	22,014,435	21,835,924	9,167,349	5,950,193 (27%)
CK-05-TF-pcDNA3-HNRNPA2B1-72h-5_S5	22,117,742	21,910,863	9,771,096	6,597,004 (30%)
CK-06-TF-pcDNA3-HNRNPA2B1-72h-6_S6	18,504,433	18,331,286	10,380,835	6,995,677 (38%)
CK-07-control-1_S7	17,326,241	16,985,944	10,930,185	7,428,171 (43%)
CK-08-control-2_S8	19,453,438	19,132,289	12,334,834	8,904,535 (46%)
CK-09-control-3_S9	20,141,938	19,825,160	13,783,978	10,009,315 (50%)
CK-10-control-4_S10	13,883,549	13,660,953	13,108,593	8,700,996 (63%)
CK-11-control-5_S11	19,675,313	19,390,808	10,567,309	8,395,825 (43%)
CK-12-control-6_S12	17,833,995	17,685,587	14,197,529	11,251,529 (63%)
CK-13-TF-pcDNA3-HNRNPA2B1-48h-1_S13	19,855,062	19,683,488	9,927,686	7,111,401 (36%)
CK-14-TF-pcDNA3-HNRNPA2B1-48h-2_S14	18,900,596	18,727,624	10,716,468	6,966,345 (37%)
CK-15-TF-pcDNA3-HNRNPA2B1-48h-3_S15	21,140,463	20,988,559	10,723,540	7,209,605 (34%)
CK-16-TF-pcDNA3-HNRNPA2B1-48h-4_S16	16,805,017	16,700,651	11,971,698	7,905,122 (47%)
CK-17-TF-pcDNA3-HNRNPA2B1-48h-5_S17	21,247,122	20,812,156	8,195,406	4,836,167 (23%)
CK-18-TF-pcDNA3-HNRNPA2B1-48h-6_S18	19,154,922	19,065,921	9,556,449	5,353,099 (28%)

References Cited in the Supplementary Materials

1 Li, S. *et al.* Genetic interaction of hnRNPA2B1 and DNAJB6 in a Drosophila model of multisystem proteinopathy. *Hum. Mol. Genet.* **25**, 936-950, doi:10.1093/hmg/ddv627 (2016).

Supplementary Table 2: Upregulated differentially expressed miRNAs in HNRNPA2B1-transfected MCF-7 cells at T48 vs. control		
**miRNA**	**logFC**	**PValue**
hsa-miR-4800-3p	4.203132779	2.13E-13
hsa-miR-4634	3.856338409	8.91E-05
hsa-miR-4653-5p	3.856338409	8.91E-05
hsa-miR-4750-5p	3.445691809	0.001801051
hsa-miR-4524b-5p	3.294704228	0.003407653
hsa-miR-3655	3.255640587	2.43E-07
hsa-miR-4755-3p	3.238031262	0.000625663
hsa-miR-762	3.232914003	0.002230912
hsa-miR-6801-3p	3.190897096	0.005168974
hsa-miR-4426	2.996799607	0.000469721
hsa-miR-4787-5p	2.976950945	2.14E-08
hsa-miR-6762-5p	2.954817975	0.006352896
hsa-miR-1266-3p	2.913436816	0.011955819
hsa-miR-6805-3p	2.787377127	3.19E-05
hsa-miR-6075	2.748673548	4.20E-05
hsa-miR-595	2.655161381	0.024368444
hsa-miR-6088	2.628207642	0.014026732
hsa-miR-4463	2.604258825	0.026240272
hsa-miR-4717-5p	2.602646258	0.028339243
hsa-miR-302c-3p	2.601994507	0.023664042
hsa-miR-4723-3p	2.598117781	0.016340663
hsa-miR-3919	2.598001404	0.016343442
hsa-miR-4752	2.598001404	0.016343442
hsa-miR-3135a	2.597961321	0.016344399
hsa-miR-587	2.597896715	0.016345943
hsa-miR-6741-3p	2.597856635	0.0163469
hsa-miR-4665-5p	2.597829519	0.016347548
hsa-miR-4739	2.597829519	0.016347548
hsa-miR-4763-5p	2.597829519	0.016347548
hsa-miR-3168	2.595217386	0.001814141
hsa-miR-222-5p	2.585461515	0.029770946
hsa-miR-3610	2.580965491	0.039047059
hsa-miR-6886-3p	2.579764101	0.027315608
hsa-miR-507	2.567778506	0.025865982
hsa-miR-3938	2.567639679	0.027324208
hsa-miR-3129-3p	2.56706982	0.022493688
hsa-miR-2861	2.548485084	0.028416533
hsa-miR-1279	2.545726931	0.026825234
hsa-miR-6773-5p	2.529326375	1.18E-05
hsa-miR-6771-5p	2.486092675	0.001475749
hsa-miR-500b-3p	2.44563351	0.01155015
hsa-miR-6803-5p	2.44529891	0.009671516

hsa-miR-3195	2.418257654	4.20E-06
hsa-miR-6501-5p	2.411379343	0.000138415
hsa-miR-3674	2.403660408	3.91E-06
hsa-miR-548g-3p	2.386207653	0.00517501
hsa-miR-4459	2.384074414	0.002203891
hsa-miR-548f-3p	2.350855566	0.007358089
hsa-miR-548f-3p	2.350775788	0.007372239
hsa-miR-7109-5p	2.312884312	0.023185593
hsa-miR-6723-5p	2.263260076	0.026585664
hsa-miR-3960	2.235150537	8.50E-05
hsa-miR-1233-3p	2.20801987	0.02415456
hsa-miR-1233-3p	2.20797134	0.024122772
hsa-miR-5581-5p	2.206518761	0.023053409
hsa-miR-6836-3p	2.206092771	0.023074595
hsa-miR-4701-3p	2.205991388	0.02307964
hsa-miR-3619-3p	2.203277202	0.014625883
hsa-miR-4667-5p	2.195984297	0.032792699
hsa-miR-6515-3p	2.183537735	0.044141557
hsa-miR-212-5p	2.152628477	0.001295284
hsa-miR-6087	2.148433791	0.002187519
hsa-miR-6882-5p	2.12889908	2.79E-07
hsa-miR-4693-3p	2.127446086	0.049901697
hsa-miR-4657	2.127446086	0.049901697
hsa-miR-7107-5p	2.127446086	0.049901697
hsa-miR-5572	2.127413287	0.049903366
hsa-miR-8079	2.127283864	0.049909961
hsa-miR-4679	2.12717909	0.049915302
hsa-miR-4679	2.12717909	0.049915302
hsa-miR-5188	2.12717909	0.049915302
hsa-miR-1538	2.12717909	0.049915302
hsa-miR-3923	2.12717909	0.049915302
hsa-miR-3132	2.127151954	0.049916685
hsa-miR-4532	2.069501044	6.23E-05
hsa-miR-3944-5p	2.003208936	0.035398427
hsa-miR-410-5p	2.00087536	0.014945206
hsa-miR-3138	1.990672417	0.003365158
hsa-miR-211-5p	1.981966671	0.022625785
hsa-miR-1468-3p	1.956609125	0.023503195
hsa-miR-4792	1.927817515	0.000284473
hsa-miR-942-3p	1.917028485	0.00105593
hsa-miR-1281	1.916344682	0.003719344
hsa-miR-6869-5p	1.900132948	0.044563921
hsa-miR-3656	1.850742661	0.003269216
hsa-miR-3619-5p	1.829995954	0.035294567
hsa-miR-3668	1.822187476	0.001375766
hsa-miR-1908-3p	1.810974842	0.020532159
hsa-miR-7114-5p	1.781494419	0.017656164

hsa-miR-8065	1.768588083	0.00023352
hsa-miR-6765-3p	1.69190989	5.70E-06
hsa-miR-4492	1.669306565	0.002163861
hsa-miR-3146	1.65991476	0.008765938
hsa-miR-644a	1.63782716	0.044484196
hsa-miR-6786-3p	1.632459207	0.046054005
hsa-miR-4516	1.629894947	0.002372425
hsa-miR-1273h-5p	1.578873985	0.018151627
hsa-miR-4772-5p	1.566919919	0.000957383
hsa-miR-466	1.564612895	0.04274115
hsa-miR-4745-3p	1.502356416	0.017071051
hsa-miR-4488	1.500744832	0.000327622
hsa-miR-6130	1.432405951	0.030094866
hsa-miR-3620-5p	1.430119946	0.022626372
hsa-miR-371a-3p	1.429123177	0.004331546
hsa-miR-548at-5p	1.427288372	0.028918441
hsa-miR-3129-5p	1.422403159	0.034625678
hsa-miR-8086	1.421223817	4.90E-05
hsa-miR-1268a	1.410789761	0.000173521
hsa-miR-4738-3p	1.410573418	0.043702259
hsa-miR-217	1.386989799	0.021144816
hsa-miR-6510-3p	1.377703955	0.041409307
hsa-miR-3976	1.366176528	0.013647042
hsa-miR-499b-5p	1.359261379	0.039421505
hsa-miR-766-5p	1.352357453	0.000233347
hsa-miR-1268b	1.346726629	0.000374145
hsa-miR-3944-3p	1.315132892	0.040381618
hsa-miR-548aq-5p	1.314317228	0.014340822
hsa-miR-5091	1.29704663	0.046264845
hsa-miR-549a	1.295860794	0.0308179
hsa-miR-6768-5p	1.287933915	0.041251419
hsa-miR-3179	1.286396992	0.033781745
hsa-miR-3179	1.286393569	0.033698158
hsa-miR-3179	1.286391499	0.033647561
hsa-miR-3179	1.286388211	0.033567054
hsa-miR-4273	1.251837003	0.002112764
hsa-miR-1343-3p	1.234162433	0.045119397
hsa-miR-1908-5p	1.2277933	0.005533894
hsa-miR-4639-5p	1.226186819	0.04621075
hsa-miR-574-5p	1.216883307	0.025954293
hsa-miR-1244	1.202043001	0.030706933
hsa-miR-1244	1.201735145	0.031202851
hsa-miR-1244	1.20170748	0.03124771
hsa-miR-1244	1.201681569	0.031289772
hsa-miR-4497	1.182764524	0.005572369
hsa-miR-92b-5p	1.127695243	0.006300523
hsa-miR-4677-5p	1.110336783	0.003663761

hsa-miR-1273g-3p	1.104494071	0.03552572
hsa-miR-212-3p	1.103164321	0.042747968
hsa-miR-4680-3p	1.049113343	0.002387136
hsa-miR-6811-5p	1.011175962	0.044360161
hsa-miR-4662b	1.007819836	0.001707278
hsa-miR-1910-3p	0.991051599	0.010488889
hsa-miR-5690	0.970305124	0.033836141
hsa-miR-2276-3p	0.930575618	0.034286101
hsa-miR-1285-5p	0.908218659	0.022601078
hsa-miR-5100	0.731914964	0.030379696
hsa-miR-4677-3p	0.697518508	0.044506676
hsa-miR-671-5p	0.596643075	0.041299365

Supplementary Table 3: Downregulated miRNAs differentially expressed in HNRNPA2B1-transfected MCF-7 cells at T48 vs. control			
**miRNA**	**logFC**	**PValue**	
hsa-miR-548ao-3p	-3.78539748	0.003172509	
hsa-miR-1323	-3.59794814	0.008542317	
hsa-miR-515-5p	-3.56620124	0.001360777	
hsa-miR-515-5p	-3.56616856	0.001353822	
hsa-miR-5692a	-3.51366668	0.001937032	
hsa-miR-5692a	-3.51366668	0.001937032	
hsa-miR-518c-3p	-3.39141739	0.000749401	
hsa-miR-3125	-3.3755672	0.003272051	
hsa-miR-3663-5p	-2.95646849	0.023834165	
hsa-miR-100-5p	-2.90174158	0.002423172	
hsa-miR-6795-3p	-2.8992754	0.019072607	
hsa-miR-3591-5p	-2.88174185	0.037661813	
hsa-miR-224-5p	-2.85908659	0.016281795	
hsa-miR-4724-5p	-2.83035637	0.021431284	
hsa-miR-488-5p	-2.82979943	0.02162979	
hsa-miR-4724-3p	-2.63449903	0.028308223	
hsa-miR-520g-3p	-2.63448753	0.028308726	
hsa-miR-5584-5p	-2.63337748	0.036290957	
hsa-miR-5008-3p	-2.63154995	0.002928978	
hsa-miR-6794-3p	-2.56511404	0.043495809	
hsa-miR-4764-3p	-2.3701118	0.049758764	
hsa-miR-511-5p	-2.37006925	0.049761458	
hsa-miR-4419a	-2.3699596	0.049768349	
hsa-miR-6834-5p	-2.36991084	0.049771395	
hsa-miR-6878-5p	-2.36991084	0.049771395	
hsa-miR-518d-5p	-2.36978535	0.049779284	
hsa-miR-520c-5p	-2.36978535	0.049779284	
hsa-miR-526a	-2.36978535	0.049779284	
hsa-miR-526a	-2.36978535	0.049779284	
hsa-miR-4793-3p	-2.36976703	0.049780434	
hsa-miR-134-3p	-2.36958444	0.049791916	
hsa-miR-5682	-2.36958444	0.049791916	
hsa-miR-4738-5p	-2.36945221	0.049800226	
hsa-miR-34c-5p	-2.35542088	0.008616474	
hsa-miR-4458	-2.34175631	0.00203071	
hsa-miR-516a-5p	-2.31224228	0.000403793	
hsa-miR-516a-5p	-2.3122401	0.000396367	
hsa-miR-135a-5p	-2.29850329	6.16527E-06	
hsa-miR-135a-5p	-2.29688459	6.3231E-06	
hsa-miR-1283	-2.26847023	0.02507276	
hsa-miR-1283	-2.26843296	0.025117555	
hsa-miR-34b-5p	-2.19886679	0.028976803	

hsa-miR-937-3p	-2.1688452	0.036852658	
hsa-miR-4789-3p	-2.16312344	2.85608E-07	
hsa-miR-4500	-2.07948985	0.032088523	
hsa-miR-5681a	-2.07160014	0.038592042	
hsa-miR-6850-3p	-2.04889561	0.02635164	
hsa-miR-7975	-1.99886341	0.018583062	
hsa-miR-551b-3p	-1.98732792	0.020543731	
hsa-miR-145-5p	-1.85141338	0.011683859	
hsa-miR-138-5p	-1.83491706	0.002232051	
hsa-miR-138-5p	-1.75347548	0.003073259	
hsa-miR-6733-3p	-1.7445249	0.03486103	
hsa-miR-489-5p	-1.67048031	0.010616457	
hsa-miR-4790-3p	-1.65802842	0.047934055	
hsa-miR-4767	-1.63401187	0.039711545	
hsa-miR-6716-3p	-1.60950122	0.041477256	
hsa-miR-944	-1.57342929	0.025418523	
hsa-miR-934	-1.46310403	0.003522488	
hsa-miR-659-5p	-1.39136436	0.018961173	
hsa-miR-1251-5p	-1.36210713	0.002726789	
hsa-miR-486-5p	-1.23643122	0.025189623	
hsa-miR-486-5p	-1.2364223	0.025134227	
hsa-miR-424-5p	-1.18151194	0.008960107	
hsa-miR-221-3p	-1.15550528	0.013398337	
hsa-miR-101-5p	-1.08909061	0.004335057	
hsa-miR-26a-1-3p	-1.05904133	0.045107542	
hsa-miR-29a-3p	-0.96725353	0.005557327	
hsa-miR-222-3p	-0.96265644	0.048910172	
hsa-miR-187-3p	-0.9124113	0.039172011	
hsa-miR-320e	-0.88366874	0.035030824	
hsa-miR-193a-3p	-0.82188668	0.017160333	
hsa-miR-19b-3p	-0.81759176	0.006291317	
hsa-miR-19b-3p	-0.81700018	0.006366224	
hsa-miR-98-3p	-0.78182413	0.012048543	
hsa-miR-17-5p	-0.7678617	0.010601397	
hsa-let-7i-3p	-0.76299911	0.011838904	
hsa-let-7f-2-3p	-0.75484915	0.006799089	
hsa-miR-3912-3p	-0.72070476	0.016340399	
hsa-miR-497-5p	-0.71984362	0.041486009	
hsa-miR-29b-3p	-0.70990076	0.021007515	
hsa-miR-29b-3p	-0.70905306	0.021158079	
hsa-miR-19a-3p	-0.70203303	0.02023362	
hsa-miR-497-3p	-0.66475969	0.040845391	
hsa-miR-20a-5p	-0.63586682	0.043514027	
hsa-miR-101-3p	-0.60813319	0.043943246	
hsa-miR-101-3p	-0.60813013	0.043416602	
hsa-miR-652-5p	-0.60358842	0.039710281	

Supplementary Table 4: Up-regulated miRNAs differentially expressed miRs in HNRNPA2B1-transfected MCF-7 cells at T72 vs. control

miRNA	logFC	PValue		
hsa-miR-4800-3p	4.387388558	9.24E-15		
hsa-miR-4426	3.631080559	6.00E-06		
hsa-miR-4524b-5p	3.616739426	0.000839287		
hsa-miR-541-5p	3.464811288	0.000506411		
hsa-miR-6801-3p	3.031881854	0.007163981		
hsa-miR-4739	3.031167408	0.003281236		
hsa-miR-7107-5p	3.031026921	0.003282251		
hsa-miR-6795-5p	3.030382835	0.005184713		
hsa-miR-4800-5p	3.013801857	0.004568116		
hsa-miR-612	3.003030713	0.007373595		
hsa-miR-4459	2.827175457	8.13E-05		
hsa-miR-6832-3p	2.809629742	0.00631546		
hsa-miR-1266-3p	2.765644806	0.015541775		
hsa-miR-4444	2.761773321	0.010054794		
hsa-miR-1913	2.759524603	0.009867667		
hsa-miR-4525	2.756304166	0.00966634		
hsa-miR-3678-5p	2.700985543	0.029226714		
hsa-miR-4787-5p	2.465144019	2.52E-06		
hsa-miR-411-3p	2.437885496	0.01364255		
hsa-miR-6830-5p	2.435451518	0.030759514		
hsa-miR-211-5p	2.430335048	0.002770363		
hsa-miR-4487	2.419463926	0.04029009		
hsa-miR-2861	2.416820388	0.034615013		
hsa-miR-6088	2.4155076	0.022788681		
hsa-miR-4770	2.412796428	0.038259502		
hsa-miR-544a	2.409024113	0.010894807		
hsa-miR-762	2.407980404	0.02372302		
hsa-miR-378b	2.407184154	0.022726884		
hsa-miR-4703-3p	2.407119606	0.022728916		
hsa-miR-2117	2.407059264	0.022730816		
hsa-miR-6762-5p	2.407030406	0.024389224		
hsa-miR-452-5p	2.407010201	0.022732361		
hsa-miR-514b-3p	2.40700015	0.022732678		
hsa-miR-6776-5p	2.40700015	0.022732678		
hsa-miR-6805-3p	2.397952883	0.00035441		
hsa-miR-7160-3p	2.396696016	0.037811389		
hsa-miR-622	2.39431824	1.63E-05		
hsa-miR-1273g-3p	2.392190463	4.76E-06		
hsa-miR-3155b	2.389817351	0.038225918		
hsa-miR-6793-3p	2.384781753	0.037831186		
hsa-miR-4275	2.38304226	0.037411249		
hsa-miR-4528	2.376296971	0.031253727		

hsa-miR-4463	2.370896592	0.036328607		
hsa-miR-3156-5p	2.364119073	0.036568463		
hsa-miR-3156-5p	2.364104944	0.036575581		
hsa-miR-3156-5p	2.364095434	0.036580372		
hsa-miR-6807-3p	2.326449403	0.005431123		
hsa-miR-4686	2.296511011	2.66E-06		
hsa-miR-6892-3p	2.27598997	4.83E-05		
hsa-miR-6771-5p	2.263844423	0.003725443		
hsa-miR-412-3p	2.25211488	0.029819697		
hsa-miR-4667-5p	2.250041384	0.024273404		
hsa-miR-4637	2.238493102	0.033240088		
hsa-miR-3120-3p	2.234574947	0.017192123		
hsa-miR-4801	2.234141883	0.016736263		
hsa-miR-3160-5p	2.230449069	0.019191452		
hsa-miR-3160-5p	2.230362304	0.019250912		
hsa-miR-4284	2.161295013	0.000179323		
hsa-miR-5684	2.094624258	8.10E-05		
hsa-miR-377-5p	2.047230263	0.010114505		
hsa-miR-6087	2.027799858	0.003686174		
hsa-miR-6715a-3p	2.016373954	0.014266136		
hsa-miR-216a-3p	2.004361062	0.046310978		
hsa-miR-6803-5p	2.002108161	0.038031505		
hsa-miR-4763-3p	2.001161334	0.037368699		
hsa-miR-5681b	2.001033903	0.037378284		
hsa-miR-5089-5p	2.000819383	0.037394428		
hsa-miR-320e	1.993658987	1.53E-06		
hsa-miR-1273h-5p	1.988673299	0.001337381		
hsa-miR-377-3p	1.949076365	0.048194852		
hsa-miR-4792	1.915585534	0.000309091		
hsa-miR-7515	1.856362933	0.047249482		
hsa-miR-7704	1.851171123	0.001271917		
hsa-miR-4532	1.839667018	0.000337186		
hsa-miR-4492	1.773275382	0.00114045		
hsa-miR-5708	1.764122565	0.005766629		
hsa-miR-6786-3p	1.758277311	0.023290864		
hsa-miR-6821-5p	1.758129104	0.03109703		
hsa-miR-4301	1.75470772	0.005080787		
hsa-miR-6126	1.742126394	5.76E-05		
hsa-miR-3074-5p	1.732437418	0.000323516		
hsa-miR-3180-3p	1.690603131	0.017296084		
hsa-miR-3180	1.690406028	0.017375125		
hsa-miR-4516	1.689283615	0.001659584		
hsa-miR-3180	1.688899595	0.017987559		
hsa-miR-3180-3p	1.688897958	0.017988233		
hsa-miR-3180-3p	1.688632457	0.018097708		
hsa-miR-3180	1.688039145	0.018344025		
hsa-miR-3180-3p	1.687608583	0.018524227		

hsa-miR-3180	1.687532849	0.01855605		
hsa-miR-3180-3p	1.687485357	0.018576026		
hsa-miR-3180	1.687041047	0.018763629		
hsa-miR-770-5p	1.664121604	0.042201656		
hsa-miR-488-3p	1.660916212	0.043864013		
hsa-miR-3960	1.647815374	0.003158015		
hsa-miR-3655	1.635422972	0.01667399		
hsa-miR-5585-3p	1.612003882	0.005338615		
hsa-miR-3195	1.609645724	0.001851951		
hsa-miR-1281	1.60635595	0.015510872		
hsa-miR-3976	1.606143204	0.003359873		
hsa-miR-1273e	1.596329494	0.004078557		
hsa-miR-1261	1.590878518	0.000112947		
hsa-miR-656-3p	1.587682081	0.025887019		
hsa-miR-3182	1.574830437	0.00018658		
hsa-miR-1295a	1.567555507	0.039567886		
hsa-miR-766-3p	1.547868879	0.035570153		
hsa-miR-6075	1.522515749	0.018028331		
hsa-miR-1972	1.512173693	0.006085399		
hsa-miR-1972	1.512127151	0.006102678		
hsa-miR-3656	1.499468554	0.016138726		
hsa-miR-363-3p	1.493306605	0.021675259		
hsa-miR-7641	1.480851116	0.004091424		
hsa-miR-7641	1.480850753	0.004102597		
hsa-miR-548ae-3p	1.472534673	0.04260042		
hsa-miR-548ae-3p	1.472521965	0.042536839		
hsa-miR-5096	1.465099494	0.000935534		
hsa-miR-4737	1.437930076	0.017432219		
hsa-miR-4417	1.419292624	0.046799264		
hsa-miR-20b-5p	1.404702672	0.039397932		
hsa-miR-130a-3p	1.398717337	0.008605335		
hsa-miR-6811-5p	1.377471958	0.003854295		
hsa-miR-4488	1.326696212	0.001424828		
hsa-miR-4508	1.311208844	0.025365597		
hsa-miR-1908-5p	1.283262421	0.003273442		
hsa-miR-1273d	1.272747532	0.014092528		
hsa-miR-181a-5p	1.232433857	0.027523109		
hsa-miR-181a-5p	1.232207862	0.027574963		
hsa-miR-3178	1.231417088	0.014167713		
hsa-miR-106a-3p	1.221015394	0.022236132		
hsa-miR-6840-3p	1.163804159	0.045968148		
hsa-miR-1910-5p	1.158689396	0.006850163		
hsa-miR-2467-5p	1.155491413	0.010402092		
hsa-miR-6073	1.154876832	0.004605902		
hsa-miR-548x-5p	1.139239893	0.043240581		
hsa-miR-320d	1.129752305	0.000417833		
hsa-miR-320d	1.129457757	0.000419549		

hsa-miR-619-5p	1.111931086	0.012509481		
hsa-miR-3654	1.105269265	0.030802615		
hsa-miR-1305	1.093484971	0.048167918		
hsa-miR-4497	1.074710123	0.011275517		
hsa-miR-1296-3p	1.070452791	0.043925415		
hsa-miR-181a-3p	1.059651518	0.044425145		
hsa-miR-1285-5p	1.05875602	0.006289426		
hsa-miR-7705	1.037367261	0.042636068		
hsa-miR-6882-5p	1.026892367	0.014202818		
hsa-miR-548aj-5p	1.003969266	0.045960452		
hsa-miR-3174	1.003302857	0.017159856		
hsa-miR-3149	0.983011619	0.044364902		
hsa-miR-502-5p	0.975124936	0.005033391		
hsa-miR-362-5p	0.973577993	0.014780919		
hsa-miR-4306	0.959808342	0.026717062		
hsa-miR-642a-5p	0.956139543	0.016350979		
hsa-miR-320c	0.949528031	0.002193135		
hsa-miR-320c	0.945228987	0.002258145		
hsa-miR-335-3p	0.917919839	0.04461683		
hsa-miR-491-3p	0.917615322	0.029406088		
hsa-miR-3176	0.915786058	0.02355254		
hsa-miR-4783-3p	0.903952261	0.040784614		
hsa-miR-3687	0.879615249	0.047662902		
hsa-miR-3687	0.879611704	0.047618071		
hsa-miR-378e	0.87190777	0.005192904		
hsa-miR-1248	0.86771004	0.02049459		
hsa-miR-320b	0.858798966	0.004470309		
hsa-miR-320b	0.858405574	0.004507697		
hsa-miR-1297	0.838308552	0.009677737		
hsa-miR-3607-5p	0.824984454	0.048456667		
hsa-miR-378d	0.823131996	0.005947268		
hsa-miR-378d	0.820214163	0.006056412		
hsa-miR-629-3p	0.793533329	0.042402584		
hsa-miR-450b-3p	0.78015708	0.041494831		
hsa-miR-5100	0.772067066	0.021257143		
hsa-miR-582-3p	0.75703111	0.029196926		
hsa-miR-597-5p	0.74946048	0.018965914		
hsa-miR-362-3p	0.708851581	0.022091285		
hsa-miR-497-3p	0.690593308	0.031351151		
hsa-miR-5001-3p	0.685832485	0.041349224		

Supplementary Table 5: Downregulated miRNAs differentially expressed miRs in HNRNPA2B1-transfected MCF-7 cells at T72 vs. control			
**miRNA**	**logFC**	**PValue**	
hsa-miR-3118	-3.98659614	0.000278927	
hsa-miR-3118	-3.98655252	0.000278303	
hsa-miR-3118	-3.98650381	0.000277608	
hsa-miR-3118	-3.98645826	0.00027696	
hsa-miR-2053	-3.81758502	0.001462807	
hsa-miR-4422	-3.73589673	0.000751411	
hsa-miR-6856-3p	-3.37528763	0.001741005	
hsa-miR-1271-3p	-3.36530127	0.002152323	
hsa-miR-6767-5p	-3.22843159	0.005055009	
hsa-miR-4720-5p	-3.05490385	0.007523285	
hsa-miR-876-5p	-3.0515501	0.018288202	
hsa-miR-6789-5p	-3.05131792	0.005926018	
hsa-miR-516b-5p	-3.01209155	0.000836571	
hsa-miR-6801-5p	-3.01176697	1.42E-06	
hsa-miR-516b-5p	-3.01167729	0.000837239	
hsa-miR-515-5p	-2.98654739	0.003289361	
hsa-miR-515-5p	-2.98649782	0.003306364	
hsa-miR-5088-5p	-2.94973905	0.018011075	
hsa-miR-6850-3p	-2.92055743	0.002869198	
hsa-miR-6872-5p	-2.86282123	0.001284091	
hsa-miR-224-5p	-2.85908659	0.010522155	
hsa-miR-1227-3p	-2.85824944	0.010539466	
hsa-miR-150-5p	-2.83282183	0.023875187	
hsa-miR-4724-5p	-2.83035637	0.014504364	
hsa-miR-520a-5p	-2.75954377	0.002349324	
hsa-miR-573	-2.75337358	0.003712351	
hsa-miR-4457	-2.72783298	0.000363843	
hsa-miR-561-3p	-2.7229628	2.71E-07	
hsa-miR-4656	-2.6713853	0.024845037	
hsa-miR-520f-3p	-2.65376353	0.011419021	
hsa-miR-6833-5p	-2.64316683	0.022623923	
hsa-miR-4648	-2.63527531	0.019513536	
hsa-miR-1909-5p	-2.63512009	0.019518591	
hsa-miR-3198	-2.63464269	0.019534122	
hsa-miR-3198	-2.63464269	0.019534122	
hsa-miR-4781-5p	-2.63440784	0.019541774	
hsa-miR-7847-3p	-2.63378159	0.023352536	
hsa-miR-509-3p	-2.63275418	0.020987137	
hsa-miR-509-3p	-2.63272968	0.02100529	
hsa-miR-509-3p	-2.6327221	0.021010916	
hsa-miR-4699-5p	-2.58437786	0.000304695	
hsa-miR-580-5p	-2.51412643	0.007702937	

hsa-miR-486-3p	-2.44883114	0.008974786	
hsa-miR-486-3p	-2.44878608	0.008984641	
hsa-miR-8087	-2.44517936	0.041456564	
hsa-miR-1283	-2.44462167	0.013844862	
hsa-miR-1283	-2.44458589	0.013874706	
hsa-miR-4272	-2.42942884	0.001849293	
hsa-miR-8066	-2.37405095	0.046076305	
hsa-miR-4479	-2.37054393	0.036670576	
hsa-miR-1183	-2.37045258	0.036675163	
hsa-miR-511-5p	-2.37006925	0.036694411	
hsa-miR-346	-2.37002797	0.036696482	
hsa-miR-206	-2.37002047	0.036696844	
hsa-miR-518d-5p	-2.36978535	0.036708656	
hsa-miR-520c-5p	-2.36978535	0.036708656	
hsa-miR-526a	-2.36978535	0.036708656	
hsa-miR-526a	-2.36978535	0.036708656	
hsa-miR-4672	-2.3696531	0.036715297	
hsa-miR-4738-5p	-2.36945221	0.036725392	
hsa-miR-4430	-2.367742	0.044694624	
hsa-miR-6780a-3p	-2.3660822	0.037598875	
hsa-miR-1468-3p	-2.33543036	0.047435265	
hsa-miR-1234-3p	-2.32569807	0.04937803	
hsa-miR-938	-2.31690339	0.049906578	
hsa-miR-196b-3p	-2.31589627	0.007583231	
hsa-miR-516a-5p	-2.29690089	0.000335647	
hsa-miR-516a-5p	-2.29682072	0.000342453	
hsa-miR-3668	-2.23553925	0.019230648	
hsa-miR-548m	-2.23233203	0.022025547	
hsa-miR-3620-3p	-2.22944373	0.017044321	
hsa-miR-4697-3p	-2.21052277	0.009840362	
hsa-miR-139-3p	-2.16039315	0.04909242	
hsa-miR-548f-3p	-2.16013166	0.019738958	
hsa-miR-5008-3p	-2.10396032	0.006209091	
hsa-miR-384	-2.08704156	0.035190402	
hsa-miR-6780a-5p	-2.07571907	0.03252203	
hsa-miR-1247-3p	-2.0717553	0.007572579	
hsa-miR-518c-3p	-2.04579376	0.013216542	
hsa-miR-675-3p	-2.01987097	0.009918281	
hsa-miR-577	-1.93685243	0.043625951	
hsa-miR-4458	-1.93538158	0.004697641	
hsa-miR-548bb-5p	-1.90956066	0.019629075	
hsa-miR-1228-5p	-1.89740829	0.039893302	
hsa-miR-221-5p	-1.85747368	0.002347752	
hsa-miR-548ad-3p	-1.77464111	0.037173794	
hsa-miR-3150b-3p	-1.77021774	0.004121933	
hsa-miR-4698	-1.69492042	0.006764039	
hsa-miR-4291	-1.66284169	0.008091534	

hsa-miR-6734-5p	-1.63297996	0.037870203	
hsa-miR-153-5p	-1.60849809	0.013780349	
hsa-miR-598-3p	-1.5932102	0.006411839	
hsa-miR-194-3p	-1.58226524	0.038518822	
hsa-miR-7974	-1.54241467	0.013855496	
hsa-miR-653-3p	-1.53650641	0.000471874	
hsa-miR-3662	-1.53121801	0.042746554	
hsa-miR-653-5p	-1.53032862	0.000470632	
hsa-miR-548h-5p	-1.49940996	0.008148313	
hsa-miR-548ap-5p	-1.49386385	0.040470311	
hsa-miR-548h-5p	-1.49119658	0.008447961	
hsa-miR-548h-5p	-1.49119658	0.008459021	
hsa-miR-548h-5p	-1.49119657	0.008469304	
hsa-miR-452-3p	-1.48882424	0.045696926	
hsa-miR-877-3p	-1.48303427	0.015228877	
hsa-miR-548h-5p	-1.47939174	0.008882042	
hsa-miR-561-5p	-1.47535794	0.000908091	
hsa-miR-27b-5p	-1.44075651	0.000771788	
hsa-miR-128-1-5p	-1.41312541	0.014325447	
hsa-miR-24-1-5p	-1.40424873	0.022258196	
hsa-miR-635	-1.39309686	0.000881052	
hsa-miR-4461	-1.37418945	0.022795708	
hsa-miR-518f-3p	-1.37119381	0.028309565	
hsa-miR-504-5p	-1.36983116	0.043284791	
hsa-miR-1247-5p	-1.36059481	0.020123501	
hsa-miR-24-2-5p	-1.32614086	0.020596452	
hsa-miR-4798-5p	-1.32289011	0.032487122	
hsa-miR-489-3p	-1.31971873	0.00431513	
hsa-miR-4662a-5p	-1.24449076	0.012401856	
hsa-miR-449a	-1.24284667	0.032029359	
hsa-miR-221-3p	-1.22615132	0.00874385	
hsa-miR-32-3p	-1.20692161	0.000893909	
hsa-miR-4483	-1.20316429	0.003346005	
hsa-miR-4680-3p	-1.20292498	0.000900021	
hsa-miR-23a-5p	-1.19873878	0.031622312	
hsa-miR-378f	-1.18673122	0.003887909	
hsa-miR-1179	-1.18193434	0.023375736	
hsa-miR-4775	-1.16143451	0.01835426	
hsa-miR-296-3p	-1.14222558	0.000926566	
hsa-miR-4668-5p	-1.1382016	0.01888399	
hsa-miR-345-3p	-1.13415523	0.004972507	
hsa-miR-365a-5p	-1.12220323	0.006235243	
hsa-miR-664b-3p	-1.11586536	0.003657154	
hsa-miR-1306-3p	-1.11373162	0.010952551	
hsa-miR-222-3p	-1.10031476	0.024530721	
hsa-miR-196b-5p	-1.09693491	0.026030322	
hsa-miR-33b-3p	-1.0913708	0.027160435	

hsa-miR-342-5p	-1.04302219	0.020620466	
hsa-miR-193b-5p	-1.03283008	0.02135743	
hsa-miR-187-3p	-1.0184959	0.01949225	
hsa-miR-301b-5p	-1.00845817	0.032478672	
hsa-miR-200a-5p	-1.00712266	0.000712157	
hsa-miR-342-3p	-1.00301642	0.014469362	
hsa-miR-361-3p	-0.9767061	0.020861295	
hsa-miR-4273	-0.94134961	0.028688489	
hsa-miR-25-5p	-0.93627871	0.015635433	
hsa-miR-200c-5p	-0.91017545	0.011937915	
hsa-miR-556-5p	-0.89444429	0.0388515	
hsa-miR-27b-3p	-0.88704076	0.009548805	
hsa-let-7g-3p	-0.88331468	0.004645281	
hsa-miR-1910-3p	-0.87500893	0.049285192	
hsa-miR-200b-5p	-0.87426412	0.00680166	
hsa-miR-8086	-0.87175608	0.015205676	
hsa-miR-1260a	-0.83695742	0.020111771	
hsa-miR-27a-5p	-0.81886714	0.028714509	
hsa-miR-1260b	-0.81518737	0.023846372	
hsa-miR-296-5p	-0.80675146	0.029145559	
hsa-miR-1268b	-0.79096579	0.038608639	
hsa-miR-33a-3p	-0.78848157	0.033155132	
hsa-miR-652-3p	-0.78424765	0.008866009	
hsa-miR-1268a	-0.78413751	0.039643384	
hsa-miR-149-5p	-0.76664153	0.040472334	
hsa-miR-625-3p	-0.7599489	0.026060458	
hsa-miR-191-5p	-0.74550869	0.016506638	
hsa-miR-301a-5p	-0.73051964	0.017318934	
hsa-miR-676-3p	-0.72434453	0.039980988	
hsa-miR-4760-5p	-0.71680002	0.048057185	
hsa-miR-21-3p	-0.67613136	0.043148714	
hsa-miR-200c-3p	-0.65344461	0.035781202	
hsa-miR-140-5p	-0.64963888	0.037252581	
hsa-miR-26a-2-3p	-0.64582721	0.045628135	
hsa-miR-30d-3p	-0.61676965	0.031215624	
hsa-miR-183-5p	-0.59511067	0.044585986	

Supplementary Table 6: Up-regulated differentially expressed miRNAs in HNRNPA2B1-transfected MCF-7 cells at T72 vs. T48 (72 or 48 h post transfection).

miRNA	logFC	PValue	
hsa-miR-548ao-3p	4.798782984	0.000150972	
hsa-miR-488-5p	3.939050292	0.001031132	
hsa-miR-6832-3p	3.821068603	0.002780887	
hsa-miR-5580-3p	3.761011865	0.011862954	
hsa-miR-6795-3p	3.649441959	0.002840014	
hsa-miR-541-5p	3.464811288	0.003347484	
hsa-miR-551b-3p	3.376596733	0.000164429	
hsa-miR-3160-5p	3.268847101	0.007244979	
hsa-miR-3160-5p	3.268756603	0.007229614	
hsa-miR-134-3p	3.26404457	0.006595256	
hsa-miR-5681a	3.142712751	0.000717504	
hsa-miR-377-3p	3.084643501	0.002849329	
hsa-miR-100-5p	3.050272387	0.001709293	
hsa-miR-5692a	3.031095985	0.013120434	
hsa-miR-5692a	3.031095985	0.013120434	
hsa-miR-5089-5p	3.031048144	0.013121564	
hsa-miR-646	3.023110587	0.017915039	
hsa-miR-6857-3p	3.016029508	0.018095783	
hsa-miR-612	3.003030713	0.022309342	
hsa-miR-548y	2.984099724	0.001517265	
hsa-miR-320e	2.877327727	4.12E-11	
hsa-miR-544a	2.772452802	0.004266689	
hsa-miR-548ax	2.764176106	0.029718363	
hsa-miR-6834-5p	2.752489612	0.026498775	
hsa-miR-8080	2.750322118	0.028465175	
hsa-miR-6785-5p	2.746559744	0.02917645	
hsa-miR-4789-3p	2.741994204	7.59E-11	
hsa-miR-6134	2.740864376	0.04360933	
hsa-miR-548as-3p	2.609377851	0.008742154	
hsa-miR-7975	2.607933904	0.001582121	
hsa-miR-136-5p	2.590915621	0.017019496	
hsa-miR-376c-5p	2.54867982	0.008778861	
hsa-miR-4790-5p	2.53535855	0.00855015	
hsa-miR-376b-5p	2.533220395	0.0127191	
hsa-miR-3126-3p	2.525549981	0.024293495	
hsa-miR-196a-3p	2.512041456	0.006054084	
hsa-miR-205-5p	2.474733447	0.000865233	
hsa-miR-136-3p	2.471440639	0.028467487	
hsa-miR-181a-2-3p	2.456821483	0.015797035	
hsa-miR-376a-5p	2.39901502	0.015660499	
hsa-miR-34b-5p	2.371347754	0.021091761	
hsa-miR-376a-2-5p	2.370396639	0.010348736	

hsa-miR-376c-3p	2.344489187	0.017450293	
hsa-miR-31-5p	2.330175016	0.011001923	
hsa-miR-488-3p	2.325698688	0.006108793	
hsa-miR-299-5p	2.324578399	0.019239439	
hsa-miR-656-3p	2.30277299	0.003973971	
hsa-miR-770-5p	2.272149783	0.008540272	
hsa-miR-4686	2.265631748	7.03E-06	
hsa-miR-154-5p	2.235125224	0.038051846	
hsa-miR-496	2.231338352	0.035589794	
hsa-miR-1972	2.214100025	0.000547115	
hsa-miR-1972	2.214086563	0.00054544	
hsa-miR-4790-3p	2.212843134	0.00858165	
hsa-miR-7112-3p	2.199762891	0.036145512	
hsa-miR-1197	2.190878752	0.035040441	
hsa-miR-1185-5p	2.188085082	0.037493445	
hsa-miR-1185-5p	2.188083309	0.037516702	
hsa-miR-1973	2.179936246	0.041249063	
hsa-miR-135b-5p	2.162166964	0.008396707	
hsa-miR-369-5p	2.129470575	0.041145889	
hsa-miR-409-5p	2.119753631	0.038834145	
hsa-miR-20b-5p	2.087607178	0.002749968	
hsa-miR-548x-3p	2.08434113	0.03148163	
hsa-miR-1305	2.082275826	0.001685347	
hsa-miR-410-3p	2.073525115	0.01801312	
hsa-miR-376b-3p	2.035054855	0.041446358	
hsa-miR-127-5p	2.023300203	0.048987817	
hsa-miR-377-5p	2.017379229	0.022300552	
hsa-miR-655-3p	2.006435183	0.038322368	
hsa-miR-380-3p	1.997034833	0.043479542	
hsa-miR-487a-3p	1.977634649	0.020466035	
hsa-miR-10a-5p	1.967894907	0.049683172	
hsa-miR-411-3p	1.964082651	0.046668573	
hsa-miR-181b-2-3p	1.922345666	0.018717163	
hsa-miR-431-3p	1.864881435	0.02141753	
hsa-miR-548aj-3p	1.825912123	0.005094976	
hsa-miR-338-3p	1.818300488	0.004346405	
hsa-miR-3607-5p	1.796590926	0.000525851	
hsa-miR-7641	1.755007038	0.000752885	
hsa-miR-7641	1.755006987	0.000750135	
hsa-miR-548aj-3p	1.744754588	0.007434478	
hsa-miR-135a-5p	1.732399047	0.000515243	
hsa-miR-135a-5p	1.730777737	0.000524831	
hsa-miR-1251-5p	1.713304983	0.000172515	
hsa-miR-196a-5p	1.681547949	0.04888215	
hsa-miR-31-3p	1.681449982	0.022391909	
hsa-miR-4284	1.681216437	0.003269597	
hsa-miR-196a-5p	1.680904241	0.048914185	

hsa-miR-135b-3p	1.659294352	0.04297983	
hsa-miR-4767	1.608694942	0.048193022	
hsa-miR-145-5p	1.598562057	0.043543818	
hsa-miR-548x-5p	1.596070224	0.005530608	
hsa-miR-3074-5p	1.579727284	0.001595747	
hsa-miR-181a-5p	1.576205308	0.005252501	
hsa-miR-181a-5p	1.575525591	0.005264062	
hsa-miR-363-3p	1.56526801	0.016834228	
hsa-miR-4485-3p	1.564711264	0.047637285	
hsa-miR-548ae-3p	1.558076427	0.03843743	
hsa-miR-548ae-3p	1.558047223	0.038498313	
hsa-miR-338-5p	1.556078716	0.038420956	
hsa-miR-455-5p	1.533439418	0.015717453	
hsa-miR-424-5p	1.513244061	0.000900819	
hsa-miR-5708	1.446530115	0.038019276	
hsa-miR-622	1.446173128	0.00844572	
hsa-miR-450b-3p	1.429990346	0.000754009	
hsa-miR-375	1.414994419	0.039089924	
hsa-miR-99a-3p	1.414588393	0.004365726	
hsa-miR-106a-3p	1.413901656	0.011493681	
hsa-miR-3609	1.397891682	0.000860407	
hsa-miR-548g-5p	1.390667549	0.008172312	
hsa-miR-548aj-5p	1.386149196	0.00699611	
hsa-miR-129-5p	1.373107167	0.01003137	
hsa-miR-129-5p	1.373090458	0.009999696	
hsa-miR-4454	1.372315829	0.009743182	
hsa-miR-1297	1.36849623	3.48E-05	
hsa-miR-619-5p	1.367917881	0.003408346	
hsa-miR-497-3p	1.355352995	3.68E-05	
hsa-miR-4301	1.354229684	0.034912202	
hsa-miR-2467-5p	1.35222011	0.006494766	
hsa-miR-30a-5p	1.350146382	0.006903378	
hsa-miR-3140-3p	1.339561444	0.032215041	
hsa-miR-181a-3p	1.315420215	0.013277666	
hsa-miR-4999-3p	1.309646193	0.029751228	
hsa-miR-1273g-3p	1.287696392	0.010289135	
hsa-miR-362-5p	1.280782361	0.001703758	
hsa-miR-99a-5p	1.269415267	0.010316098	
hsa-miR-320d	1.259142575	9.17E-05	
hsa-miR-320d	1.257845522	9.31E-05	
hsa-miR-5096	1.248128867	0.004880842	
hsa-miR-188-3p	1.247687746	0.004137491	
hsa-miR-138-5p	1.244618817	0.039615714	
hsa-miR-1248	1.200080716	0.002039389	
hsa-miR-450a-5p	1.198099039	0.000381602	
hsa-miR-125b-5p	1.195421384	0.010605572	
hsa-miR-125b-5p	1.19330672	0.010767824	

hsa-miR-450a-5p	1.193157943	0.000406297	
hsa-miR-193a-3p	1.184979489	0.000626583	
hsa-miR-7705	1.164332588	0.029755007	
hsa-miR-7704	1.162920912	0.041125831	
hsa-miR-503-5p	1.156641184	0.029507975	
hsa-miR-497-5p	1.146699828	0.001269376	
hsa-miR-6892-3p	1.14406472	0.032480814	
hsa-miR-378e	1.134772741	0.00034966	
hsa-miR-130a-3p	1.133485926	0.046131227	
hsa-miR-590-5p	1.125172248	0.000196545	
hsa-miR-98-3p	1.122188938	0.000341385	
hsa-miR-378d	1.09669363	0.000314839	
hsa-miR-378d	1.089310718	0.000338954	
hsa-miR-190a-3p	1.068408272	0.007938015	
hsa-miR-5684	1.062212177	0.040666913	
hsa-miR-4783-3p	1.060675328	0.021898234	
hsa-miR-320c	1.055990021	0.000692398	
hsa-miR-3127-3p	1.055236079	0.047728262	
hsa-miR-320c	1.047853316	0.000745136	
hsa-miR-3065-5p	1.039235813	0.028172317	
hsa-miR-3194-5p	1.036486687	0.012708119	
hsa-miR-642a-5p	1.03232583	0.013146855	
hsa-miR-181b-5p	1.014145501	0.040605348	
hsa-miR-532-5p	1.002510131	0.005847367	
hsa-miR-335-3p	0.994058895	0.030082998	
hsa-miR-181b-5p	0.992529226	0.044689061	
hsa-miR-190a-5p	0.98493279	0.006682727	
hsa-miR-5701	0.9807906	0.022618384	
hsa-miR-5701	0.980789272	0.022656456	
hsa-miR-5701	0.980787915	0.022695368	
hsa-miR-4306	0.972300311	0.035528329	
hsa-miR-491-3p	0.961644409	0.025668573	
hsa-miR-3687	0.959229096	0.036224198	
hsa-miR-3687	0.959225734	0.036261052	
hsa-miR-3065-3p	0.951743972	0.027841203	
hsa-miR-320b	0.942058838	0.001879965	
hsa-miR-28-5p	0.940678123	0.025833653	
hsa-miR-320b	0.939478669	0.001943558	
hsa-miR-28-3p	0.930728362	0.036686843	
hsa-miR-597-5p	0.922375667	0.005127756	
hsa-miR-6126	0.903511037	0.032756452	
hsa-miR-93-5p	0.87561697	0.015064332	
hsa-miR-3182	0.854728006	0.039233067	
hsa-miR-570-3p	0.824436902	0.014177125	
hsa-miR-1261	0.823081904	0.042034291	
hsa-miR-660-5p	0.811600646	0.010146839	
hsa-miR-499a-5p	0.799038853	0.006699265	

hsa-miR-188-5p	0.78041204	0.013287292	
hsa-miR-29a-3p	0.76598689	0.027586924	
hsa-miR-33a-5p	0.760843438	0.02478115	
hsa-miR-18b-5p	0.747122901	0.014775107	
hsa-miR-548a-3p	0.738371427	0.035266239	
hsa-miR-627-5p	0.735337798	0.015430168	
hsa-miR-3912-3p	0.72358293	0.016893264	
hsa-miR-502-5p	0.720365548	0.041721634	
hsa-miR-548a-3p	0.719844554	0.040079561	
hsa-miR-548a-3p	0.719843463	0.040094876	
hsa-miR-93-3p	0.673401899	0.034793153	
hsa-miR-195-5p	0.667746531	0.037777332	
hsa-miR-548z	0.64378397	0.025654499	
hsa-miR-19b-3p	0.633976405	0.033664244	
hsa-miR-19b-3p	0.632565882	0.034186246	
hsa-miR-19a-3p	0.628061981	0.037598099	
hsa-miR-548h-3p	0.622038086	0.031069554	
hsa-miR-652-5p	0.586198302	0.046044116	
hsa-miR-130b-3p	0.55613417	0.048001779	
hsa-miR-378c	0.549562596	0.041825875	
hsa-miR-301b-3p	0.547922474	0.047201315	

Supplementary Table 7: Downregulated differentially expressed miRNAs in HNRNPA2B1-transfected MCF-7 cells at T72 vs. T48			
**miRNA**	**logFC**	**PValue**	
hsa-miR-1468-3p	-4.29203948	0.00025208	
hsa-miR-3668	-4.05772673	8.38E-07	
hsa-miR-2053	-4.05598993	0.001010057	
hsa-miR-8066	-4.02264523	0.000812077	
hsa-miR-4634	-3.85633841	0.000428975	
hsa-miR-3674	-3.82774472	2.48E-08	
hsa-miR-4422	-3.67845813	0.001653559	
hsa-miR-6789-5p	-3.47647584	0.002352989	
hsa-miR-4720-5p	-3.46665414	0.003233139	
hsa-miR-548ah-5p	-3.40627199	0.015650424	
hsa-miR-3118	-3.266352	0.006769053	
hsa-miR-3118	-3.26628947	0.006765238	
hsa-miR-3118	-3.26621969	0.006760985	
hsa-miR-3118	-3.26615448	0.006757015	
hsa-miR-6836-3p	-3.23608579	0.005356877	
hsa-miR-4648	-3.23608337	0.005356905	
hsa-miR-6840-5p	-3.21723467	0.007277345	
hsa-miR-4430	-3.21687647	0.008733436	
hsa-miR-3190-5p	-3.16805212	0.017362612	
hsa-miR-4457	-3.16062851	4.20E-05	
hsa-miR-6801-5p	-3.103833	1.08E-06	
hsa-miR-520a-5p	-3.02223978	0.001299258	
hsa-miR-942-3p	-3.01815872	5.25E-05	
hsa-miR-516b-5p	-3.00795514	0.001626622	
hsa-miR-516b-5p	-3.00753282	0.001627985	
hsa-miR-4786-5p	-2.98781602	0.016827012	
hsa-miR-466	-2.96176249	0.002505183	
hsa-miR-6856-3p	-2.95156158	0.012742179	
hsa-miR-6804-5p	-2.95037055	0.013267696	
hsa-miR-1271-3p	-2.94931588	0.013635153	
hsa-miR-6792-5p	-2.94845931	0.013437558	
hsa-miR-7847-3p	-2.94810529	0.015420378	
hsa-miR-580-5p	-2.92340402	0.002380551	
hsa-miR-5586-5p	-2.91895928	0.020508542	
hsa-miR-4699-5p	-2.73612521	0.00024968	
hsa-miR-6499-3p	-2.72970273	0.047655909	
hsa-miR-548g-3p	-2.7266703	0.005004347	
hsa-miR-561-3p	-2.72306681	5.09E-07	
hsa-miR-606	-2.69603959	0.04213303	
hsa-miR-6810-5p	-2.61038173	0.043624006	
hsa-miR-548m	-2.59920636	0.009720116	
hsa-miR-139-3p	-2.59872527	0.019955857	

hsa-miR-5088-5p	-2.59852814	0.042937277	
hsa-miR-1915-3p	-2.59842477	0.030924555	
hsa-miR-4723-3p	-2.59811778	0.030936968	
hsa-miR-3919	-2.5980014	0.030941677	
hsa-miR-4752	-2.5980014	0.030941677	
hsa-miR-4781-5p	-2.59798597	0.030942302	
hsa-miR-3198	-2.59796132	0.030943298	
hsa-miR-3198	-2.59796132	0.030943298	
hsa-miR-4509	-2.59785664	0.030947534	
hsa-miR-4509	-2.59785664	0.030947534	
hsa-miR-4509	-2.59785664	0.030947534	
hsa-miR-3193	-2.59782382	0.030948859	
hsa-miR-1229-5p	-2.59782382	0.030948859	
hsa-miR-222-5p	-2.58546152	0.049083769	
hsa-miR-196b-3p	-2.56701139	0.003929099	
hsa-miR-6510-3p	-2.56043749	0.001606735	
hsa-miR-1267	-2.54657377	0.011662608	
hsa-miR-1279	-2.54572693	0.045627345	
hsa-miR-1243	-2.45246821	0.002520484	
hsa-miR-3662	-2.41930734	0.001110205	
hsa-miR-644a	-2.39524729	0.014700357	
hsa-miR-6891-5p	-2.39404245	0.014149976	
hsa-miR-603	-2.39341097	0.013829209	
hsa-miR-410-5p	-2.39253076	0.009626374	
hsa-miR-1247-3p	-2.38866204	0.002560301	
hsa-miR-573	-2.34693096	0.019507493	
hsa-miR-6773-5p	-2.34060203	8.59E-05	
hsa-miR-8086	-2.2929799	4.05E-10	
hsa-miR-4680-3p	-2.25203832	9.69E-10	
hsa-miR-548f-3p	-2.23294333	0.021518505	
hsa-miR-548bb-5p	-2.2204861	0.009087525	
hsa-miR-4272	-2.21395111	0.010042521	
hsa-miR-4501	-2.20672295	0.003932894	
hsa-miR-1268a	-2.19492728	1.87E-08	
hsa-miR-4273	-2.19318661	4.30E-07	
hsa-miR-4748	-2.19047867	0.028437842	
hsa-miR-1322	-2.14159433	0.001944712	
hsa-miR-1268b	-2.13769242	4.97E-08	
hsa-miR-452-3p	-2.12205324	0.004951719	
hsa-miR-3146	-2.11114552	0.004101838	
hsa-miR-3168	-2.10918539	0.011054682	
hsa-miR-3150b-3p	-2.10082668	0.000741153	
hsa-miR-3141	-2.07401761	0.028771418	
hsa-miR-548aq-5p	-2.06856687	0.000509875	
hsa-miR-548f-3p	-2.03168093	0.026223023	
hsa-miR-548f-3p	-2.03161936	0.026257398	
hsa-miR-4775	-2.00022049	4.20E-05	

hsa-miR-635	-1.98545529	2.82E-06	
hsa-miR-1180-5p	-1.95670773	0.045885421	
hsa-miR-5579-3p	-1.91762549	0.034492288	
hsa-miR-6744-3p	-1.91256858	0.036000008	
hsa-miR-8065	-1.91101622	0.000192742	
hsa-miR-4254	-1.90555373	0.03943488	
hsa-miR-6734-5p	-1.90483636	0.018337529	
hsa-miR-4653-5p	-1.90403687	0.027372397	
hsa-miR-4697-3p	-1.89411637	0.04316632	
hsa-miR-153-5p	-1.89202705	0.004605775	
hsa-miR-3163	-1.89115991	0.040778781	
hsa-miR-676-5p	-1.88281501	0.017975426	
hsa-miR-7974	-1.88223972	0.003601907	
hsa-miR-4654	-1.87976012	0.015698813	
hsa-miR-3138	-1.8785616	0.009300821	
hsa-miR-1910-3p	-1.86606053	1.75E-05	
hsa-miR-548ap-5p	-1.84610013	0.013383919	
hsa-miR-504-5p	-1.83579166	0.007475297	
hsa-miR-598-3p	-1.82834342	0.001896769	
hsa-miR-24-1-5p	-1.82199337	0.003360739	
hsa-miR-518f-3p	-1.78480627	0.005280496	
hsa-miR-1306-3p	-1.7705948	4.74E-05	
hsa-miR-549a	-1.7670082	0.011244347	
hsa-miR-2116-3p	-1.76190734	0.042453896	
hsa-miR-24-2-5p	-1.73242476	0.002758406	
hsa-miR-4798-5p	-1.69546888	0.007462502	
hsa-miR-4291	-1.68322689	0.012789277	
hsa-miR-296-3p	-1.67205411	1.69E-06	
hsa-miR-4659a-3p	-1.66419407	0.023765535	
hsa-miR-23a-5p	-1.66195447	0.003224432	
hsa-miR-553	-1.65737463	0.013992332	
hsa-miR-5007-5p	-1.65430042	0.018569183	
hsa-miR-4699-3p	-1.65003144	0.010576029	
hsa-miR-3614-5p	-1.63309543	0.000426157	
hsa-miR-3620-5p	-1.62318351	0.015294162	
hsa-miR-3655	-1.62021762	0.004032406	
hsa-miR-3944-3p	-1.61039341	0.021057484	
hsa-miR-196b-5p	-1.59411563	0.001397402	
hsa-miR-5010-5p	-1.56625312	0.048695072	
hsa-miR-6886-5p	-1.55078475	0.017066725	
hsa-miR-766-5p	-1.54468545	3.60E-05	
hsa-miR-4713-5p	-1.53334598	0.028937356	
hsa-miR-3129-5p	-1.52903678	0.03839091	
hsa-miR-4668-5p	-1.52839048	0.001907196	
hsa-miR-5690	-1.52030371	0.002626435	
hsa-miR-561-5p	-1.51169825	0.00072706	
hsa-miR-92b-5p	-1.491927	0.000414892	

hsa-miR-365a-5p	-1.4888019	0.000325291	
hsa-miR-550a-3-5p	-1.47329921	0.005139061	
hsa-miR-550a-5p	-1.47327071	0.005151507	
hsa-miR-550a-3-5p	-1.47326706	0.005153103	
hsa-miR-550a-5p	-1.47323656	0.005166467	
hsa-miR-371a-3p	-1.42004121	0.006776783	
hsa-miR-6504-5p	-1.41900867	0.046144981	
hsa-miR-1247-5p	-1.40480007	0.021814613	
hsa-miR-6501-5p	-1.40426938	0.018273102	
hsa-miR-155-5p	-1.40114591	0.012009835	
hsa-miR-548h-5p	-1.39566723	0.013883041	
hsa-miR-548h-5p	-1.39563651	0.014004885	
hsa-miR-548h-5p	-1.39563244	0.014021131	
hsa-miR-548h-5p	-1.39562865	0.014036235	
hsa-miR-548h-5p	-1.39561829	0.014077691	
hsa-miR-574-5p	-1.38723955	0.011503929	
hsa-miR-27a-5p	-1.38208548	0.000262	
hsa-miR-25-5p	-1.37273359	0.00045744	
hsa-miR-664b-3p	-1.37253417	0.00039987	
hsa-miR-342-5p	-1.3398054	0.00313965	
hsa-miR-212-5p	-1.32143682	0.040598216	
hsa-miR-193b-5p	-1.31823449	0.003530734	
hsa-miR-1255a	-1.26160096	0.003419297	
hsa-miR-610	-1.24485315	0.027139329	
hsa-miR-3682-5p	-1.24462745	0.01350916	
hsa-miR-4662a-5p	-1.20958734	0.015790395	
hsa-miR-1276	-1.20759169	0.030966335	
hsa-miR-4778-5p	-1.18429994	0.039524623	
hsa-miR-3613-5p	-1.17587048	0.030803522	
hsa-miR-625-5p	-1.16164021	0.004283777	
hsa-miR-342-3p	-1.15327545	0.005062273	
hsa-miR-653-5p	-1.14953639	0.008020461	
hsa-miR-676-3p	-1.13300048	0.001461551	
hsa-miR-3613-3p	-1.13006512	0.044458508	
hsa-miR-4662b	-1.12649099	0.000636016	
hsa-miR-548ab	-1.1255856	0.003833564	
hsa-miR-6882-5p	-1.10200671	0.005350842	
hsa-miR-4483	-1.10039552	0.0072592	
hsa-miR-219a-1-3p	-1.09935604	0.022469535	
hsa-miR-1260a	-1.08455686	0.002732771	
hsa-miR-1260b	-1.08183644	0.002858296	
hsa-miR-489-3p	-1.06209552	0.020894185	
hsa-miR-32-3p	-1.0619186	0.003469635	
hsa-miR-4446-3p	-1.05810062	0.009539585	
hsa-miR-1266-5p	-1.04937496	0.01394833	
hsa-miR-4640-5p	-1.04596187	0.018602296	
hsa-miR-4451	-1.01614105	0.025375645	

hsa-miR-877-5p	-1.00784841	0.003673406	
hsa-miR-653-3p	-0.99231331	0.022217282	
hsa-miR-200b-5p	-0.98829956	0.002291785	
hsa-miR-2276-3p	-0.9828769	0.027326027	
hsa-miR-6765-3p	-0.97259472	0.007869194	
hsa-miR-361-3p	-0.96808924	0.022042262	
hsa-miR-2277-3p	-0.96451394	0.04790759	
hsa-miR-4677-3p	-0.95657545	0.007253153	
hsa-miR-149-5p	-0.94820481	0.011531974	
hsa-miR-4772-5p	-0.94727231	0.042474026	
hsa-miR-21-3p	-0.93152984	0.005520017	
hsa-miR-625-3p	-0.9293842	0.006660582	
hsa-miR-556-5p	-0.92636623	0.034003149	
hsa-miR-3136-5p	-0.92428715	0.024982754	
hsa-let-7d-3p	-0.92199508	0.012184259	
hsa-miR-200a-5p	-0.92028385	0.001986385	
hsa-miR-4677-5p	-0.9118264	0.018682835	
hsa-miR-4714-3p	-0.90508653	0.024596792	
hsa-miR-760	-0.88534061	0.033546597	
hsa-miR-550a-3p	-0.87801623	0.040220076	
hsa-miR-550a-3p	-0.87781731	0.040999303	
hsa-miR-550a-3p	-0.87761213	0.041819016	
hsa-miR-491-5p	-0.86589983	0.028952288	
hsa-miR-744-5p	-0.86121834	0.003247995	
hsa-miR-197-3p	-0.84392294	0.008335137	
hsa-miR-556-3p	-0.82263591	0.03759322	
hsa-miR-1307-3p	-0.82242736	0.008049954	
hsa-miR-1275	-0.79880247	0.007909428	
hsa-miR-574-3p	-0.79268265	0.030687971	
hsa-miR-3157-5p	-0.78464475	0.042346622	
hsa-miR-941	-0.77635579	0.01130435	
hsa-miR-941	-0.77635541	0.011342305	
hsa-miR-941	-0.77635512	0.011370623	
hsa-miR-941	-0.77635476	0.01140735	
hsa-miR-941	-0.7763544	0.011443473	
hsa-miR-3187-3p	-0.77398378	0.032182578	
hsa-miR-4760-5p	-0.71625194	0.048575675	
hsa-miR-193a-5p	-0.71472042	0.027060505	
hsa-miR-664a-3p	-0.70555882	0.048741756	
hsa-miR-5699-3p	-0.68387155	0.015236217	
hsa-miR-421	-0.67067125	0.042278412	
hsa-miR-190b	-0.66788207	0.029789956	
hsa-miR-200c-3p	-0.64826566	0.037262207	
hsa-miR-652-3p	-0.63435676	0.03426398	
hsa-miR-423-5p	-0.60388234	0.037265768	
hsa-miR-183-5p	-0.59791835	0.043606468	

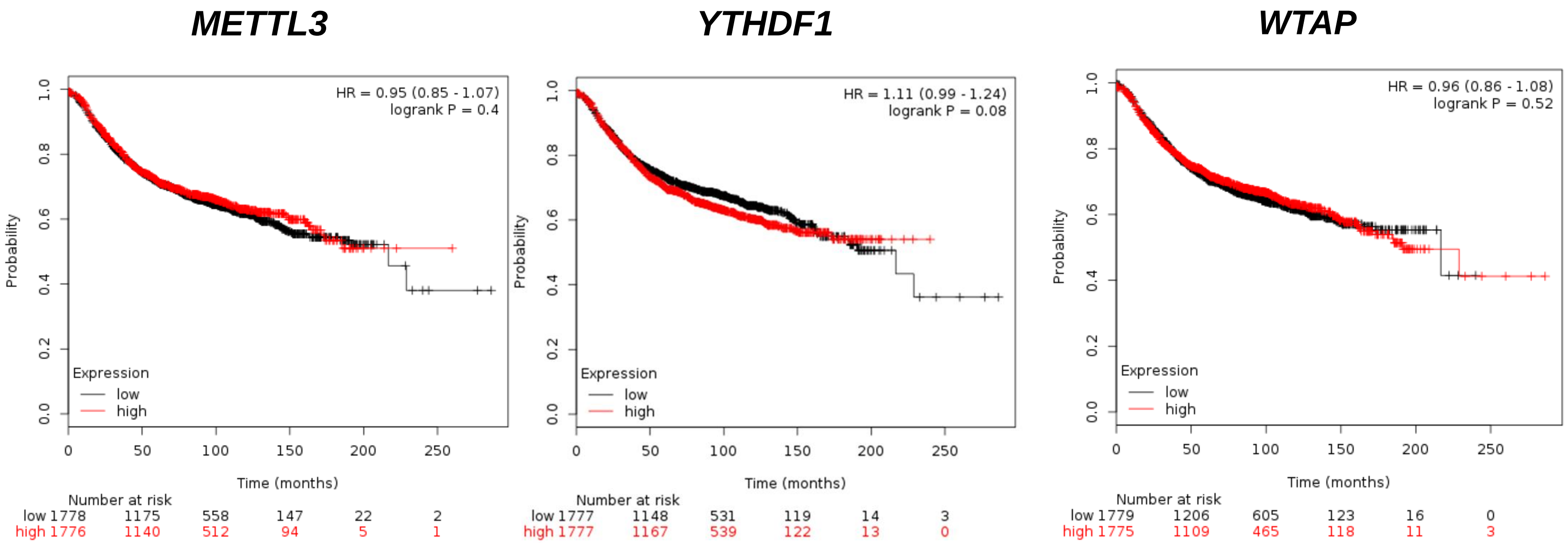

Supplementary Figure 1: Kaplan–Meier estimator plots of the probability of overall survival of breast cancer patients based on primary tumor expression of the indicated gene. Data were from 3,554 breast tumors and are not sorted for ER or HER2 status. Data are from http://kmplot.com.

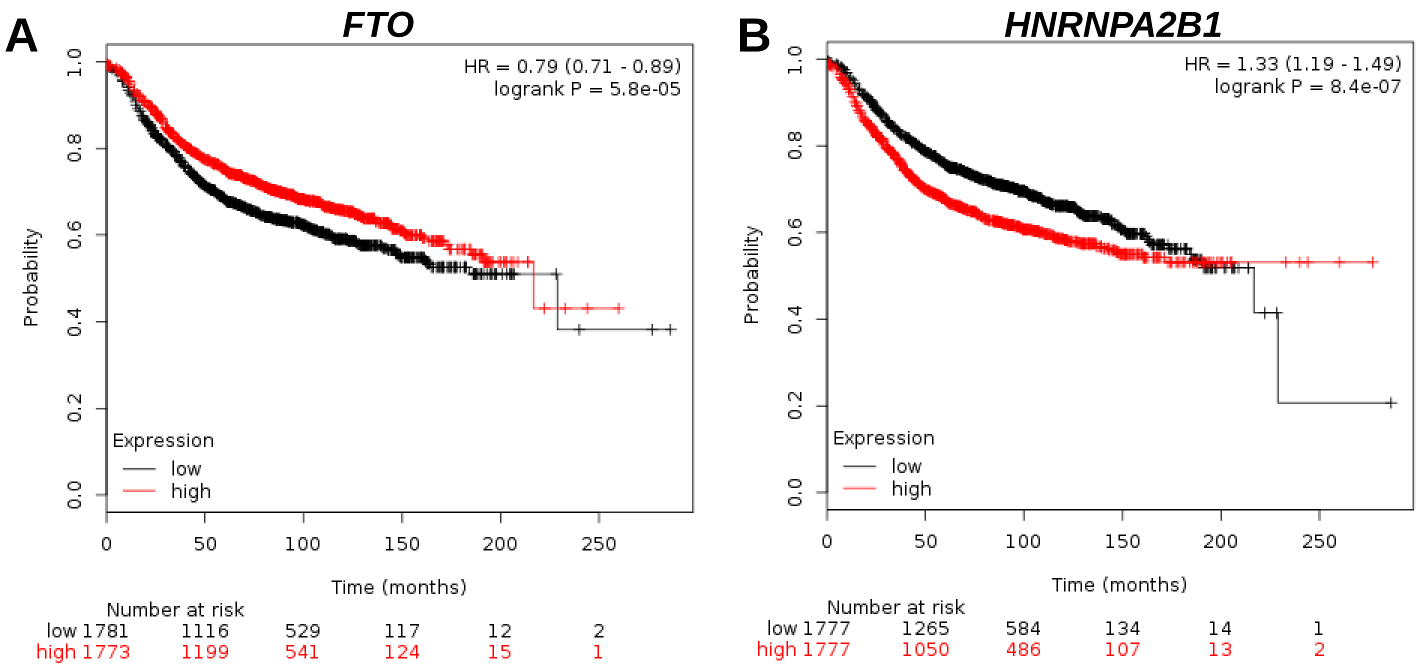

Supplementary Figure 2: Kaplan–Meier estimator plots of the probability of overall survival of breast cancer patients based on primary tumor expression of the indicated gene. Data from http://kmplot.com.

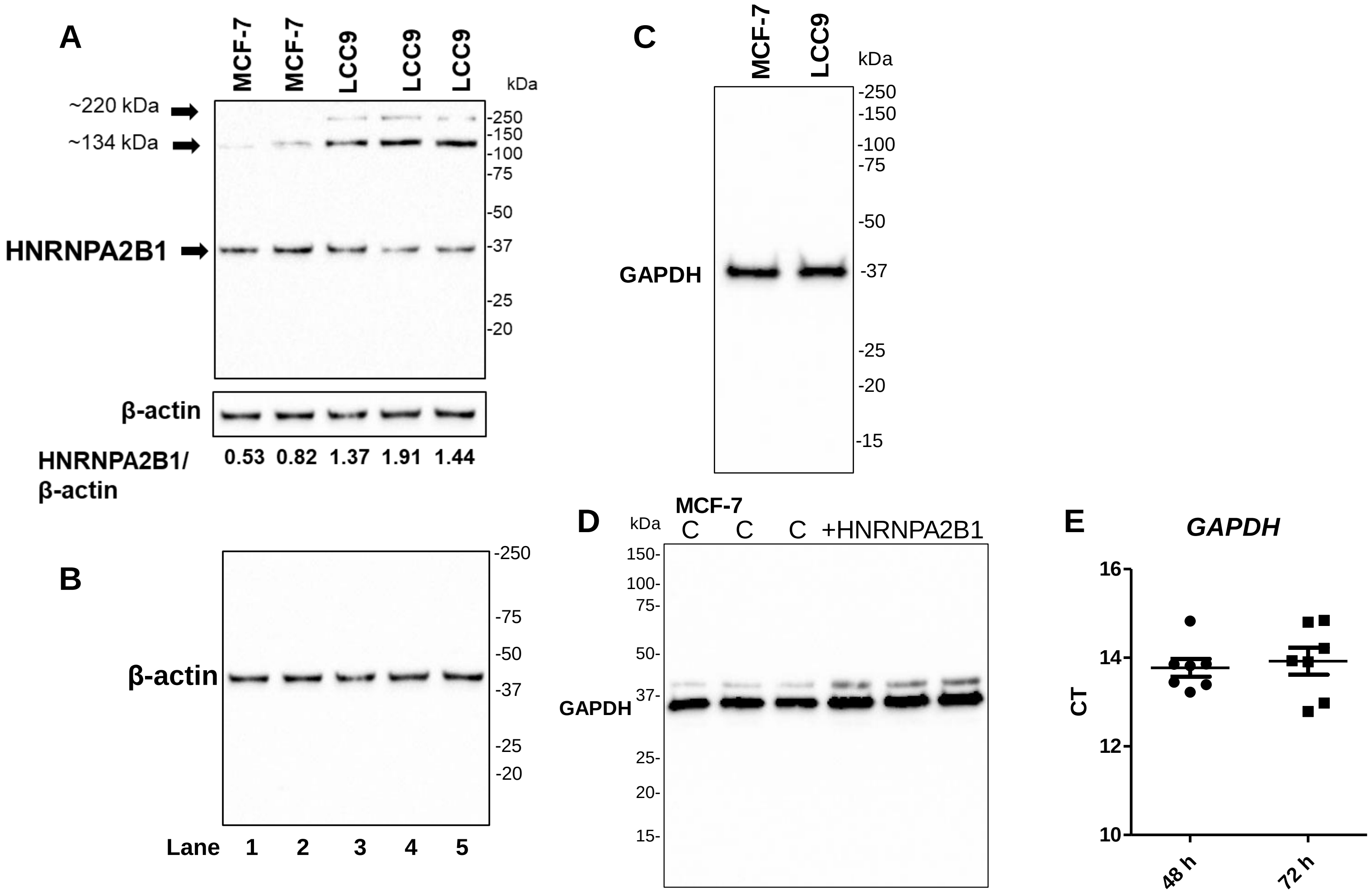

Supplementary Figure 3: HNRNPA2B1 protein in MCF-7 and LCC9 cells; Full length GAPDH blots for Fig 1; and qPCR of GAPHD in cells transfected with pcDNA3 control for 48 or 72 h. A) For this gel, 45 g of WCE were separated on a 10% SDS-PAGE gel and transferred to a PVDF membrane, probed with IBL NRNPA2B1 (catalog #18941), stripped and reprobed for β-actin. Values are HNRNPA2B1/ β-actin for the lanes shown. While HNRNPA2B1 is a 37 kDa protein it is known to form aggregates (Li *et al* Hum Mol Genet 2016). The estimated MW of the HNRNPA2B1 aggregates was calculated using UnScanIt. B) Full blot of β-actin from A is shown. C) Full blot of GAPDH for Figure 1C. D) Full blot of GAPDH for Figure 2B. E) qPCR for *GAPH* using TaqMan primers was run on 7 samples (each in quadruplicate) from MCF-7 cells transfected with pcDNA3 for 48 or 72 h. Values are the CT for GAPDH in each sample with the avg. ± stdev. plotted. There is no significant difference when analyzed by a two-tailed t-test in GraphPad Prism.

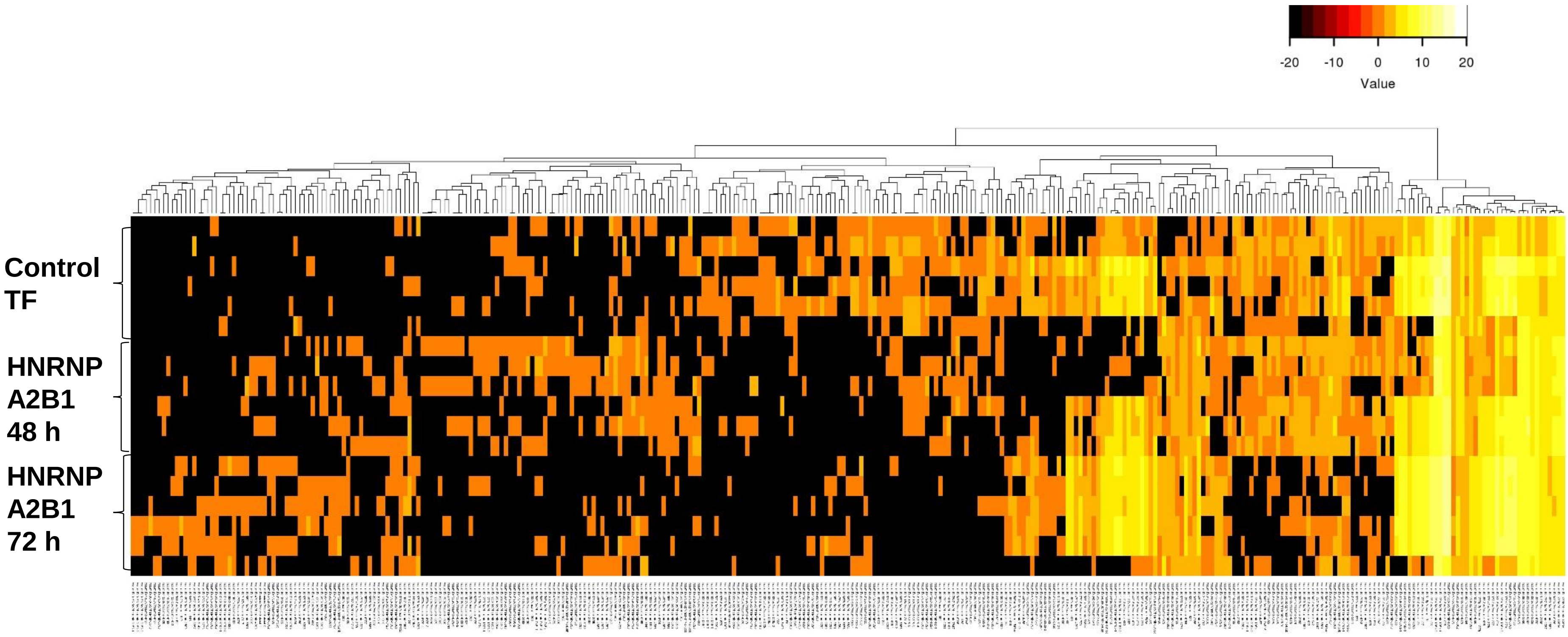

Supplementary Figure 4: Effect of HNRNPA2B1 transfection in MCF-7 cells for 48 or 72 h on miRNA expression. Heatmap represents the raw expression values with miRNAs clustered based on similar expression profiles. The scale is at the top right. Only miRNA transcripts that showed a log2 fold-change greater than 1 (or -1 for repressed miRNAs) were included. miRNAs down-regulated are black and upregulated yellow to white.

1. miRNAs in melanoma
2. Regulation of miRNAs in colorectal cancer
3. Role of miRNAs in cell proliferation in colorectal cancer
4. miRNA in prostate cancer
5. Role of epigenetic alterations in survival and migration of SCLC cells
6. Role of miRNAs in cell migration, survival and angiogenesis in colorectal cancer
7. Role of epigenetic alterations in proliferation and differentiation of SCLC cells
8. Stem cells: Hypothetical role of miRNAs in fibrosis development after myocardial infarction
9. PR action in breast cancer: stimulation of metastasis
10. EZH2 in prostate cancer

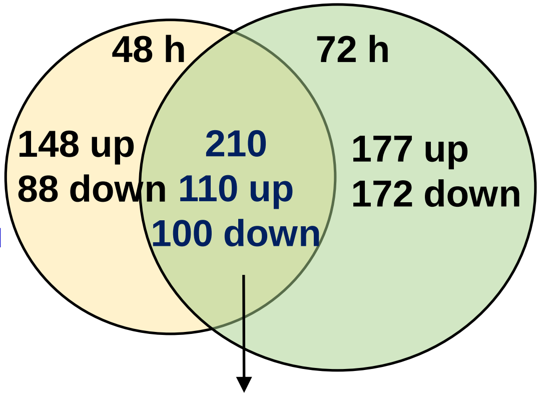

1. miRNAs in melanoma
2. miRNA in prostate cancer
3. Apoptotic pathways and resistance to apoptosis in lung cancer cells
4. Immune response IL-10 signaling pathway
5. Role of miRNAs in cell proliferation in colorectal cancer
6. Downregulation of MITF in melanoma
7. TGFbeta signaling via SMADs in breast cancer
8. Role of miRNAs in cell migration, survival and angiogenesis in colorectal cancer
9. Main pathways of Schwann cells transformation in neurofibromatosis type 1
10. Aberrant lipid trafficking and metabolism in age-related macular degeneration pathways

1. TGF-beta signaling via miRNA in breast cancer
2. Suppression of p53 signaling in multiple myeloma
3. miRNA in prostate cancer
4. miRNAs in melanoma
5. Role of miRNAs in cell proliferation in colorectal cancer
6. Development miRNA-dependent regulation of EMT
7. Role of miRNAs in cell migration, survival and angiogenesis in colorectal cancer
8. LKB1 signaling pathway in lung cancer cells
9. Role of epigenetic alterations in proliferation and differentiation of SCLC cells
10. Activation of Notch signaling in breast cancer

Supplementary Figure 5: Venn diagram depicting the number of different and common miRNAs identified as HNRNPA2B1 regulated in MCF-7 cells after 48 or 72 h of transfection. MetaCore Enrichment by Pathway Maps analysis of DE miRNAs (both up and downregulated) after 48 h and 72 h (both versus control) identified common (blue) and unique (black) pathways putatively regulated by the DE miRNAs.

miRNAs increased upon HNRNPA2B1 overexpression

GO processes

1. Cellular response to amino acid stimulus (miR- 222, 92b, 217)
2. Cellular response to estrogen stimulus (miR-574, 466)
3. Response to amino acid (miR- 222, 92b, 217)
4. Cellular response to acid chemical (miR- 222, 92b, 217)
5. Cellular response to chemical stimulus (miR- 222, 574 ,92b, 410, 217, 466)
6. Response to oxygen levels (miR- 222, 574, 92b)
7. Cellular response to oxygen-containing compound (miR- 222, 92b, 410, 217)
8. Response to estrogen (miR-574, 466)
9. Response to acid chemical l(miR- 222, 92b, 217)
10. Cellular response to organonitrogen compound (miR- 222, 92b, 217)

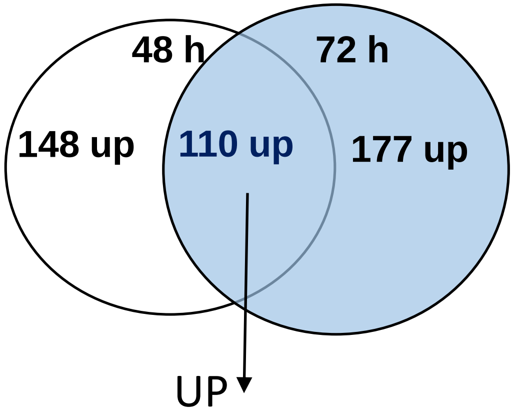

1. Cellular response to inorganic substance
2. Cellular response to chemical stimulus
3. Response to inorganic substance
4. Hematopoietic stem cell differentiation
5. Cellular response to amino acid stimulus
6. Response to chemical
7. Juxtaglomerular apparatus development (miR-125b)
8. Cellular response to oxygen-containing compound
9. Response to amino acid
10. Myelination (miR-195, 33, 30a, 138, 100)

1. Cellular response to inorganic substance (miR-130a, 20b, 106a)
2. Response to inorganic substance (miR-130a, 20b, 106a)
3. Type B pancreatic cell development (miR-541)
4. Cellular response to lipopolysaccharide (miR-181a)
5. Cellular response to molecule of bacterial origin (miR-181a)
6. Type B pancreatic cell differentiation (miR-541)
7. Cellular response to biotic stimulus (miR-181a)
8. Enteroendocrine cell differentiation (miR-541)
9. Hematopoietic stem cell differentiation (miR-130a)
10. Glandular epithelial cell development (miR-541)

Supplementary Figure 6: Venn diagram depicting the number of different and common miRNAs identified as upregulated after transient HNRNPA2B1 overexpression in MCF-7 cells after 48 or 72 h. MetaCore Enrichment by GO Processes analysis of DE miRNAs upregulated after 48 h and 72 h (both versus control) identified common to 48 h (blue), common to 72 h (red) and unique (black) pathways putatively regulated by the DE miRNAs.

miRNAs decreased upon HNRNPA2B1 overexpression

GO processes

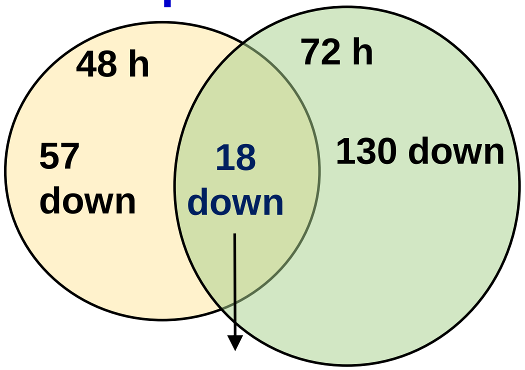

1. Pre-B-cell differentiation (miR-19b-1, 19a, 20a, 17)
2. Immature B cell differentiation (miR-19b-1, 19a, 20a, 17)
3. Cellular hyperosmotic salinity response (miR-29b, 100, 29b-1, 29b-2)
4. Cellular response to salt stress (miR-29b, 100, 29b-1, 29b-2)
5. Cellular response to chemical stimulus (let-7i, miR-138-2, 652, 29b, 100, 17, 29b-1, 29b-2, 145, 134, 101-1, 34c, 193, 29a, 138-1. 26a-1)
6. Cellular hyperosmotic response (miR-29b, 100, 29b-1, 29b-2)
7. Hyperosmotic salinity response (miR-29b, 100, 29b-1, 29b-2)
8. Spinal cord motor neuron differentiation (miR-19b-1, 19a, 20a, 17)
9. Ventricular septum morphogenesis (miR-19b-1, 19a, 20a, 17)
10. Hyperosmotic response (miR-29b, 100, 29b-1, 29b-2)

1. Cellular response to estrogen stimulus (miR-222, 221, 224, 486)
2. Response to amino acid (miR-222, 221, 224, 486)
3. Cellular response to acid chemical (miR-222, 221, 224, 486)
4. Response to acid chemical (miR-222, 221, 224, 486)
5. Cellular response organonitrogen compound (miR-222, 221, 224, 486)
6. Cellular response to nitrogen compound (miR-222, 221, 224, 486)
7. Response organonitrogen compound (miR-222, 221, 224, 486)
8. Cellular response to oxygen-containing compound (miR-222, 221, 224, 486)
9. WNT signaling pathway Cellular response organonitrogen compound (miR-222, 221, 224)
10. Cell-cell signaling by Wnt (miR-222, 221, 224)

1. mRNA cleavage (miR-196b)
2. Cellular response to estrogen stimulus (miR-206)
3. Regulation of B cell differentiation (miR-150)
4. RNA phosphodiester bond hydrolysis (miR-196b)
5. Response to estrogen (miR-206)
6. Cellular response to amino acid stimulus (miR-346)
7. Cellular response to chemical stimulus (miR-150, 206, 346)
8. Immunoglobulin production (miR-150)
9. Cellular response to oxygen-containing compound (miR-150, miR-346)
10. Production of molecular mediator of immune response (miR-150)

Supplementary Figure 7: Venn diagram depicting the number of different and common miRNAs identified as downregulated after transient HNRNPA2B1 overexpression in MCF-7 cells after 48 or 72 h. MetaCore Enrichment by GO processes of DE downregulated miRNAs after 48 h and 72 h (both versus control) identified as GO processes putatively regulated by the DE miRNAs.

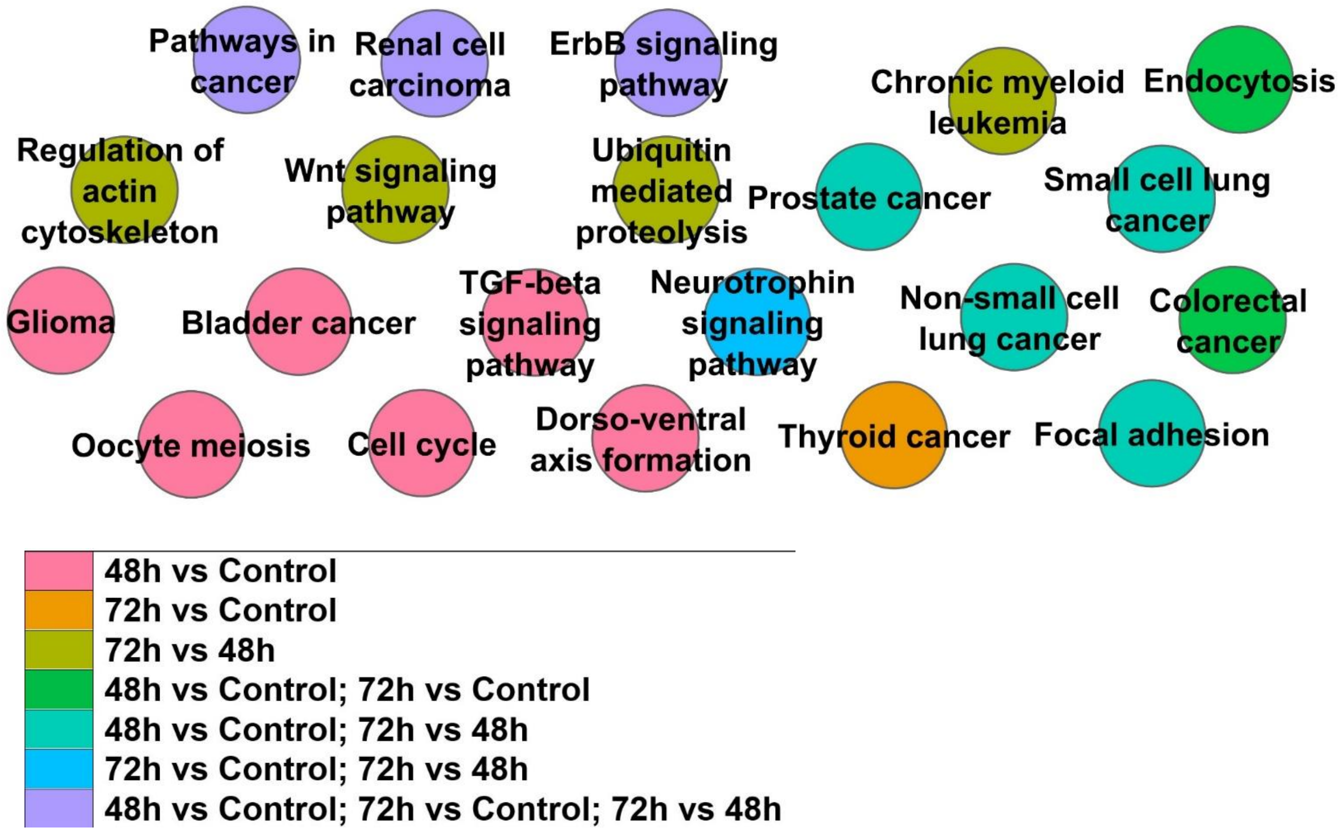

Supplementary Figure 8: Enriched KEGG for genes targeted by differentially expressed miRNAs at the indicated times.
